# mTOR kinase is a therapeutic target for respiratory syncytial virus and coronaviruses

**DOI:** 10.1038/s41598-021-03814-7

**Published:** 2021-12-24

**Authors:** HoangDinh Huynh, Ruth Levitz, Rong Huang, Jeffrey S. Kahn

**Affiliations:** 1 Department of Pediatrics, University of Texas Southwestern Medical Center, Dallas, TX 75390 USA; 2 Department of Research Administration Children’s Medical Center, Dallas, TX 75235 USA; 3 Department of Microbiology, University of Texas Southwestern Medical Center, Dallas, TX 75390 USA

**Keywords:** Viral pathogenesis, Viral host response

## Abstract

Therapeutic interventions targeting viral infections remain a significant challenge for both the medical and scientific communities. While specific antiviral agents have shown success as therapeutics, viral resistance inevitably develops, making many of these approaches ineffective. This inescapable obstacle warrants alternative approaches, such as the targeting of host cellular factors. Respiratory syncytial virus (RSV), the major respiratory pathogen of infants and children worldwide, causes respiratory tract infection ranging from mild upper respiratory tract symptoms to severe life-threatening lower respiratory tract disease. Despite the fact that the molecular biology of the virus, which was originally discovered in 1956, is well described, there is no vaccine or effective antiviral treatment against RSV infection. Here, we demonstrate that targeting host factors, specifically, mTOR signaling, reduces RSV protein production and generation of infectious progeny virus. Further, we show that this approach can be generalizable as inhibition of mTOR kinases reduces coronavirus gene expression, mRNA transcription and protein production. Overall, defining virus replication-dependent host functions may be an effective means to combat viral infections, particularly in the absence of antiviral drugs.

## Introduction

Respiratory syncytial virus (RSV) is the major respiratory pathogen of infants and children worldwide^1^. Globally, RSV accounts for ~ 200,000 deaths per year in children less than 5 years old, making RSV the 3rd most common cause of death secondary to pneumonia in this age group (after *Streptococcus pneumoniae* and *Haemophilus influenzae*)^2^. The clinical manifestations of RSV infection in young children range from mild upper respiratory tract symptoms to severe life-threatening lower respiratory tract disease. The epidemiology of RSV is complex and dynamic. RSV strains can be classified, based on serological and genetic methods, into 1 of 2 subgroups (A or B) and both subgroup A and B viruses circulate during each seasonal epidemic^3,4^. Strains in a community vary from year to year and strains identified in one location may be similar to or quite distinct from strains identified in different years in vastly different geographic locations.

RSV is a single-stranded, negative sense, non-segmented enveloped RNA virus^5^. The genome is ~ 15,000 bases and encodes at least 11 genes, some of which are encoded in overlapping reading frames; the intergenic distances are relatively short^6^. The genome encodes three virion glycoproteins: fusion (F), attachment (G), and short hydrophobic (SH)^7^. The F protein is an essential component of the virus and is responsible for viral attachment and the classic “syncytia” observed in cell culture^8^. The nucleocapsid (N), and matrix (M) genes encode structural proteins of the virion and tend to be highly conserved among isolates. RSV replicates entirely in the cytoplasm without involvement of the nucleus^9^ and can replicate in cells devoid of nuclei^10^.

The management of RSV has changed little since it was first isolated from symptomatic children in 1957^11^ (the virus was originally discovered in chimpanzees in 1956^12^). There is no effective vaccine to protect against RSV infection. Antiviral therapy is of limited effectiveness at best and is rarely used in the management of RSV infection^13^. The treatment of the RSV infected child is essentially supportive care (supplemental oxygen, hydration, invasive or non-invasive respiratory support, if necessary). A humanized monoclonal antibody (palivizumab) was approved by the FDA in 1998 to protect high risk infants against severe RSV disease as a prophylactic agent, however this product is not approved for use in the vast majority of otherwise healthy infants and does not protect against RSV infection. In addition, this product has no therapeutic effectiveness^14^.

At present, there are several promising antiviral drugs in development and several have advanced to clinical trials. These agents can be classified into four categories: immunoglobulins, nucleoside analogues, small interfering RNAs, and fusion inhibitors (small molecules)^15,16^. These drugs were developed to target virus-specific molecules and functions. Although these drugs are promising, one foreseeable obstacle is the development of viral resistance, as genomic mutations occur during viral replication (this is true for all RNA viruses)^17,18^. Therefore, alternative therapeutic approaches should be considered.

RSV utilizes specific host intracellular pathway(s) for viral replication and viral protein synthesis^18^ and these pathways are potential targets for antiviral therapy. Therefore, we hypothesized that interfering with specific host cellular function(s) required for RSV protein synthesis would mitigate or eliminate viral growth, regardless of viral mutations. There are many host cellular processes that involve protein synthesis and several of these pathways are potential targets for cancer therapeutics. One pathway is the mechanistic target of rapamycin (mTOR) signaling, known to regulate many fundamental cellular processes, including protein synthesis^19^. mTOR is a serine/threonine protein kinase that belongs to the phosphoinositide 3-kinase (PI3K)-related kinase family^19^. mTOR kinase interacts with several proteins to form two distinct complexes, known as mTOR complex 1 (mTORC1) and mTOR complex 2 (mTORC2). mTORC1 contains the regulatory subunit Raptor, which is sensitive to rapamycin, while mTORC2, which contains Rictor, is rapamycin-insensitive. Aside from protein synthesis, mTOR signaling plays a key role in many fundamental cellular processes, including energy metabolism, lipogenesis, autophagy, lysosome biogenesis, cytoskeletal organization, cell growth and survival^19^. To evaluate the role of mTOR complexes in the RSV replication cycle, we used various clinical isolates of RSV previously characterized^20–22^.

To address whether this approach, in which mTOR-associated pathways are specifically targeted, could be exploited to inhibit protein synthesis or generation of infectious particles of viruses other than RSV, we chose to use our system to examine the effect of mTOR kinase inhibition on coronaviruses given the current COVID-19 pandemic. Endemic common human coronaviruses (HCoVs), such as 229E, NL-63, OC43, and HKU-1 are responsible for mild respiratory illnesses like the common cold^23^ though these viruses can cause severe or life-threatening disease^24^. In the last 2 decades, 3 highly pathogenic human coronaviruses have emerged: severe acute respiratory syndrome coronavirus 1 (SARS-CoV-1) (2002)^25^, Middle East respiratory syndrome coronavirus (MERS-CoV) (2012)^26^, and the severe acute respiratory syndrome coronavirus 2 (SARS-CoV-2) (2019), now known to cause current pandemic disease COVID-19^27^. HCoV 229E and NL-63 belong to genus alpha-coronavirus, whereas HCoV OC43, HKU-1, SARS-CoV-1, MERS-CoV, and SARS-CoV-2 belong to beta-coronavirus genus. There is currently a great need for effective strategies to treat HCoV infections. Here we show that targeting mTOR, as an antiviral approach, is generalizable to at least one human coronavirus and may be an effective strategy against highly pathogenic coronaviruses such as SARS-CoV-1, MERS-CoV and SARS-CoV-2.

## Results

### Pharmacologic inhibition of mTORC1 increases viral protein synthesis and generation of infectious progeny virus

**Figure 1:**
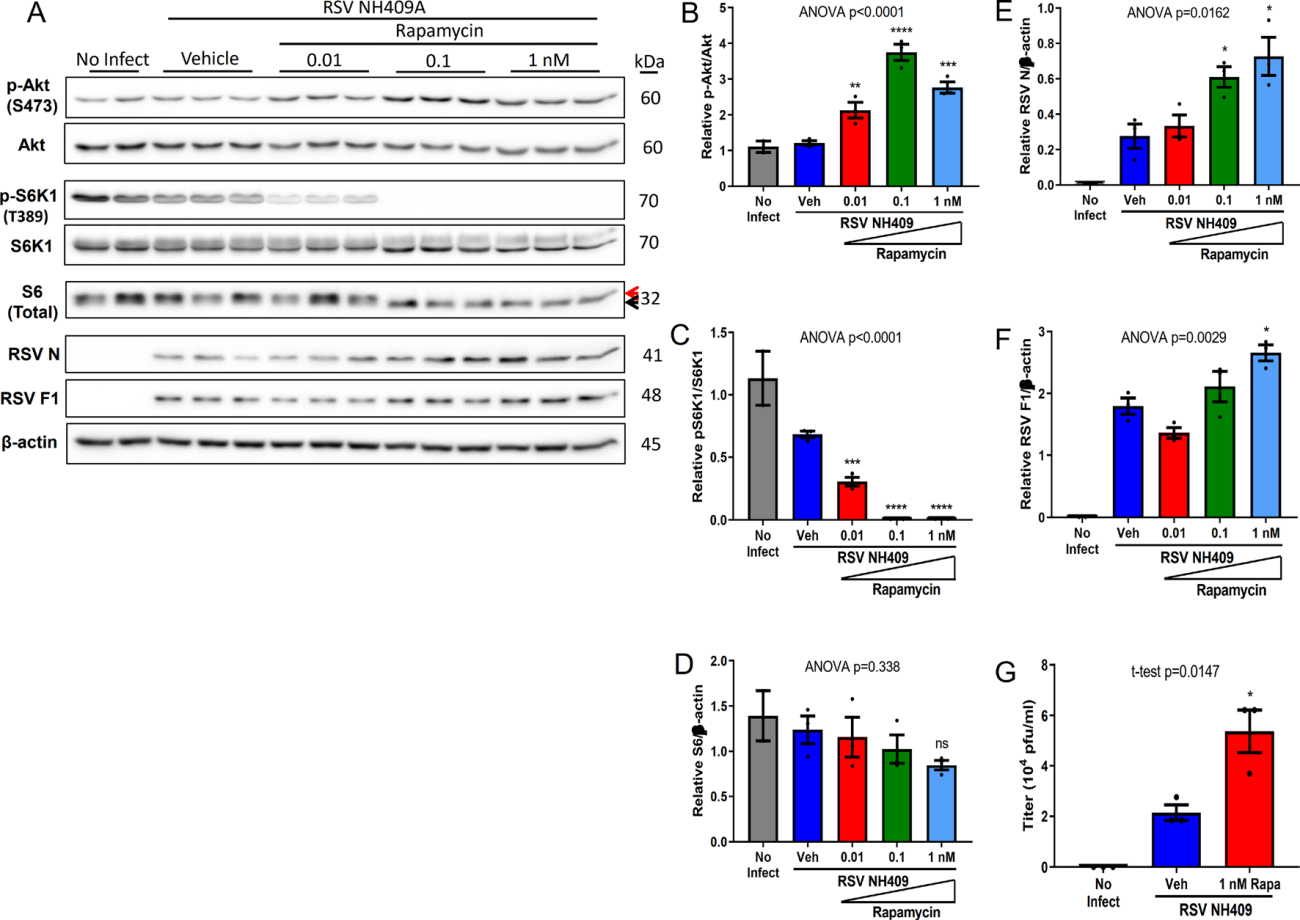
Rapamycin enhances viral protein expression and generation of progeny virus. A549 cells were infected with clinical isolate NH409A and subsequently treated with varying concentrations of rapamycin. Protein analyses and production of progeny virus was measured at 24 h post infection (m.o.i = 0.2). Asterisk (*) or non-significant (ns) is compared to vehicle (Veh) control. (**A**) Western blot of cellular and viral proteins. The 2 forms of S6 are designated by the red and black arrows. (**B**–**F**) Quantification of cellular and viral proteins displayed in (**A**) (n = 3). (**B**) phospho-Akt/Akt; (**C**) phospho-S6K1/S6K1; (**D**) total ribosomal protein S6/β-actin; (**E**) RSV nucleoprotein N/β-actin; (**F**) RSV fusion protein F1/β-actin. (**G**) Quantification of virus progeny in the absence and presence of rapamycin (n = 3). Error bars, SEM; *, *p* < 0.05; **, *p* < 0.01; ***, *p* < 0.001; ****, *p* < 0.0001; *n.s.* non-significant.

We have previously characterized RSV clinical isolates in terms of severity of illness^22^ and induction of the innate immune system^21^. Among the subgroup A and B strains selected for this study, there was a varying degree of viral protein synthesis during the viral replication cycle (Supplementary Fig. 1A–C). From these, we arbitrarily selected NH409A, a subgroup A strain, to evaluate whether mTORC1 has any significant role in RSV protein synthesis or generation of infectious particles. A549 cells were infected with RSV strain NH409A for 1.5 hours^20,22^. Infected A549 cells were then treated at varying doses with rapamycin (sirolimus), an mTORC1-specific inhibitor^28–30^ for 22.5 h. Western blotting and plaque titration assays were performed at 24 h post-infection. We chose this time point as our previous studies have shown that the induction of the cellular innate immune response occurs within this time frame in RSV-infected cells^21^. At low dose (0.1 nM), rapamycin completely inhibited S6K1 phosphorylation, a read-out for mTORC1 activity and this correlated with the activation (phosphorylation) of Akt, a well-known downstream target for mTORC2 (Fig. 1A–C). Consistent with the reduced level of S6K1 phosphorylation, rapamycin treatment predictably decreased phosphorylation of ribosomal protein S6 (upper band, Fig. 1A, D). Inhibition of mTORC1 by rapamycin resulted a statistically significant induction of viral nucleocapsid N and fusion F1 proteins (Fig. 1E,F). Functionally, elevated viral protein levels also correlated with statistically significant increase in the production of virus progeny (Fig. 1G). Further, induction of viral proteins and the generation of progeny virus was observed at high doses of rapamycin (10–100 nM) (Supplementary Fig. 2A–E), though the maximal effect appears to be in the range of 1–10 nM. These observations were replicated with NH1101A, a subgroup A3 RSV isolate (Supplementary Fig. 3A–D), and NH1125B, a B subgroup RSV isolate (Supplementary Fig. 4A–I) indicating that the effect of rapamycin is generalizable across RSV strains. These initial observations warranted further mechanistic studies of how inhibition of mTORC1 signaling induced RSV protein synthesis and generation of progeny virus.

### Akt inhibitor enhances viral protein synthesis and generation of infectious progeny virus

**Figure 2:**
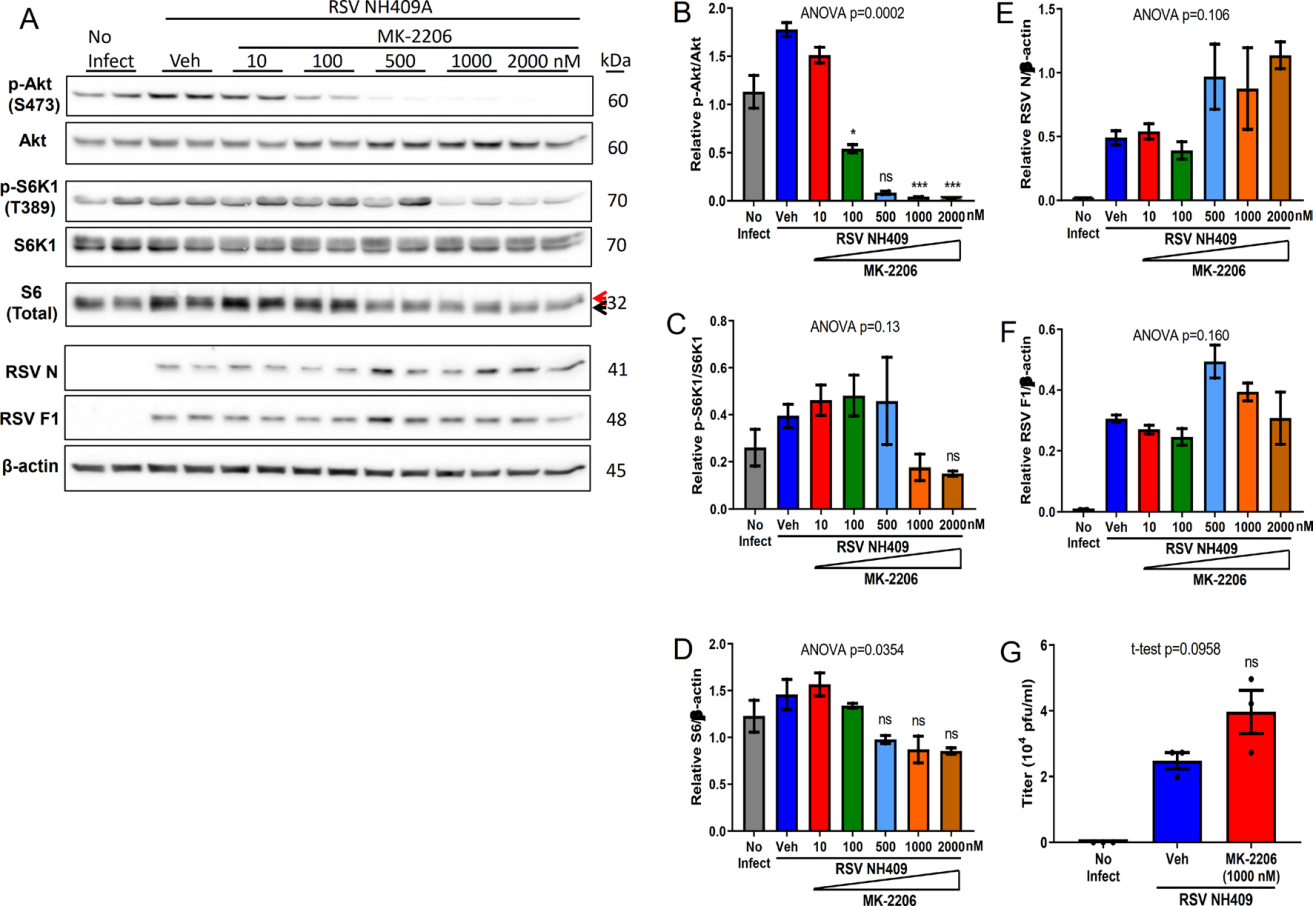
Akt inhibitor enhances viral proteins and the generation of infectious progeny virus. A549 cells were infected with clinical isolate NH409A and subsequently treated with varying concentrations of MK-2206, an Akt inhibitor. Protein analyses and the generation of infectious progeny virus was measured at 24 h post infection (m.o.i = 0.2). Asterisk (*) or non-significant (ns) is compared to vehicle (Veh) control. (**A**) Western blot of cellular and viral proteins. The 2 forms of S6 are designated by the red and black arrows. (**B**–**F**) Quantification of cellular and viral proteins displayed in A (n = 2). (**B**) phospho-Akt/Akt; (**C**) phospho-S6K1/S6K1; (**D**) total ribosomal protein S6/β-actin; (**E**) RSV nucleoprotein N/β-actin; (**F**) RSV fusion protein F1/β-actin. (**G**) Quantification of virus progeny in the absence and presence of MK-2206 (n = 3). Error bars, SEM; *, *p* < 0.05; **, *p* < 0.01; ***, *p* < 0.001; ****, *p* < 0.0001; *n.s.* non-significant.

The inhibition of mTORC1 with rapamycin correlated with the predictable activation (phosphorylation) of Akt (Fig. 1A,B), due to the loss of negative feedback loops^31,32^. Akt, an upstream activator of mTORC1, is known to promote cell survival, proliferation, and growth^19,33,34^. Activation of Akt is also known to be involved in many cancers^35^. Our data suggested that the increased activation of Akt correlated with the induction of viral protein synthesis and viral generation of infectious virus. To determine whether Akt has any impact on RSV protein synthesis and production of infectious particles, infected A549 cells were treated with MK-2206, a known pan-Akt inhibitor^36^. Treatment with MK-2206 abolished Akt phosphorylation at 1 µM, which correlated with reduction of S6K1 activation, and total S6 (Fig. 2A–D). Inactivation of Akt correlated with modest enhancement of viral protein synthesis and the production of virus progeny (Fig. 2E–G), consistent with the findings following rapamycin treatment (Fig. 1). Our observations suggested a potential similar impact of other class of Akt inhibitors on the generation of infectious RSV progeny virus.

### mTORC1 and mTORC2 provide redundant activities for RSV generation of infectious virus

**Figure 3:**
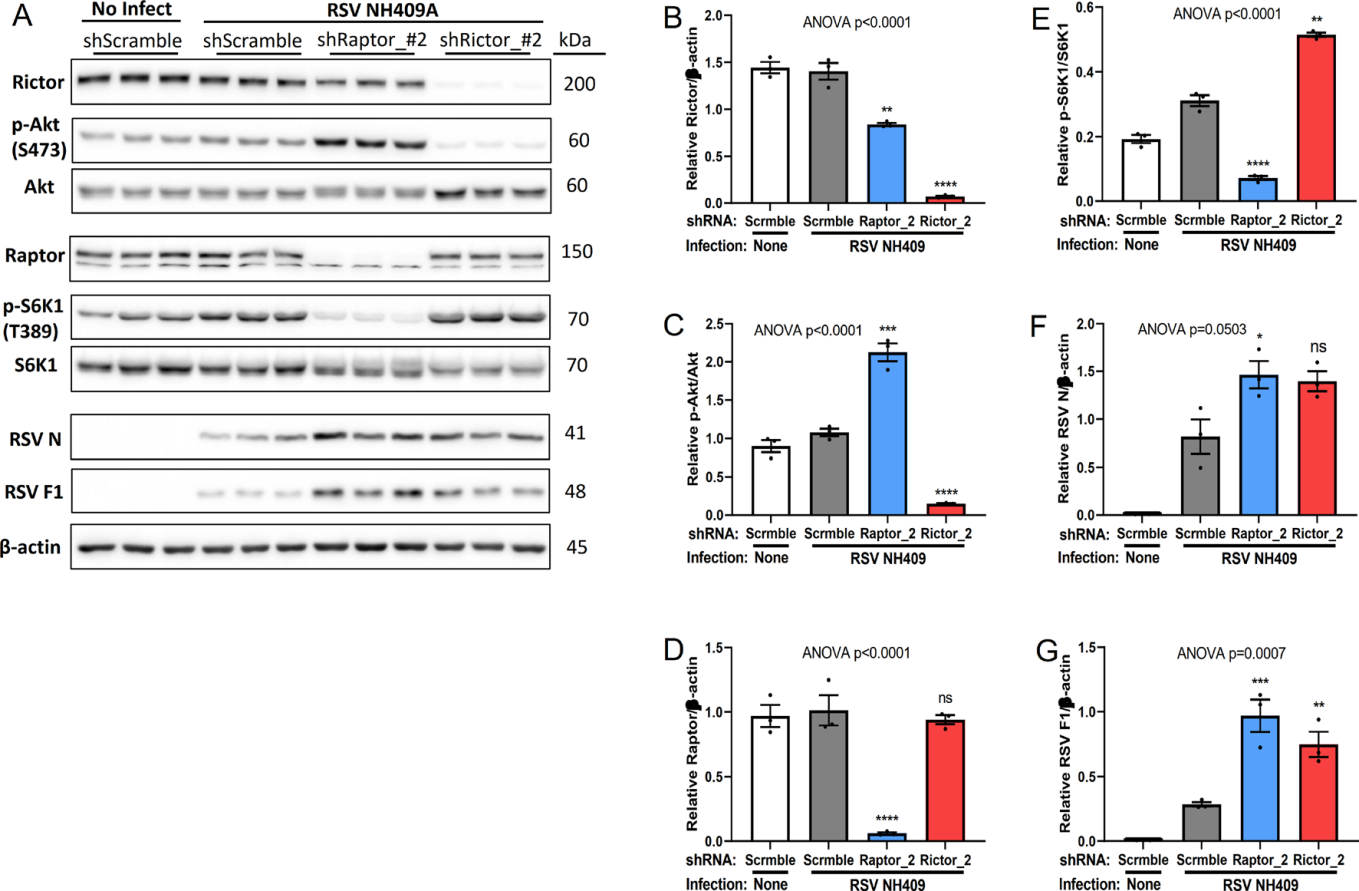
Genetic knockdown of Raptor or Rictor increases RSV protein synthesis. Lenti-viral short-hairpin RNA (shRNA) were used to knockdown expression of either Raptor or Rictor in A549 cells. shRaptor- or shRictor-A549 cells were infected with RSV NH409A. Cellular and viral protein expression was measures at 24 h post infection (m.o.i = 0.2). Asterisk (*) or non-significant (ns) is compared to Scramble/RSV control. (**A**) Western blot analysis of cellular and protein expression. (**B**–**G**) Quantification of protein synthesis displayed in panel A (n = 3). (**B**) Rictor/β-actin; (**C**) phospho-Akt/Akt; (**D**) Raptor/β-actin; (**E**) phospho-S6K1/S6K1; (**F**) RSV nucleoprotein N/β-actin; (**G**) RSV fusion protein F1/β-actin. Error bars, SEM; *, *p* < 0.05; **, *p* < 0.01; ***, *p* < 0.001; ****, *p* < 0.0001; *n.s.* non-significant.

Treatment with an Akt inhibitor MK-2206 (downstream of mTORC2) enhanced the production of infectious progeny virus, suggesting mTORC2 may be involved in viral replication. mTORC1 contains subunit Raptor, while mTORC2 contains Rictor^19^. Due to lack of proven mTORC2-specific inhibitors, we used a genetic approach to define the role of mTORC2 in RSV protein synthesis and the generation of infectious progeny virus. Raptor or Rictor was genetically knocked down by stable lenti-viral short-hairpin RNA (shRNA) (Fig. 3A & Supplementary Fig. 5)^37^. The knockdown of Raptor significantly reduced S6K1 phosphorylation, while elevating Akt phosphorylation (Fig. 3B–E), which is consistent with rapamycin treatment (Fig. 1). In similar fashion, the knockdown of Rictor significantly reduced Akt phosphorylation, while elevating S6K1 phosphorylation. Knockdown of either Raptor or Rictor correlated with increased viral protein synthesis (Fig. 3F,G). These observations suggest mTORC1 and mTORC2 have a redundant role for RSV replication as far as the generation of infectious virus.

### Knockdown of both Raptor and Rictor abolishes viral protein synthesis

**Figure 4:**
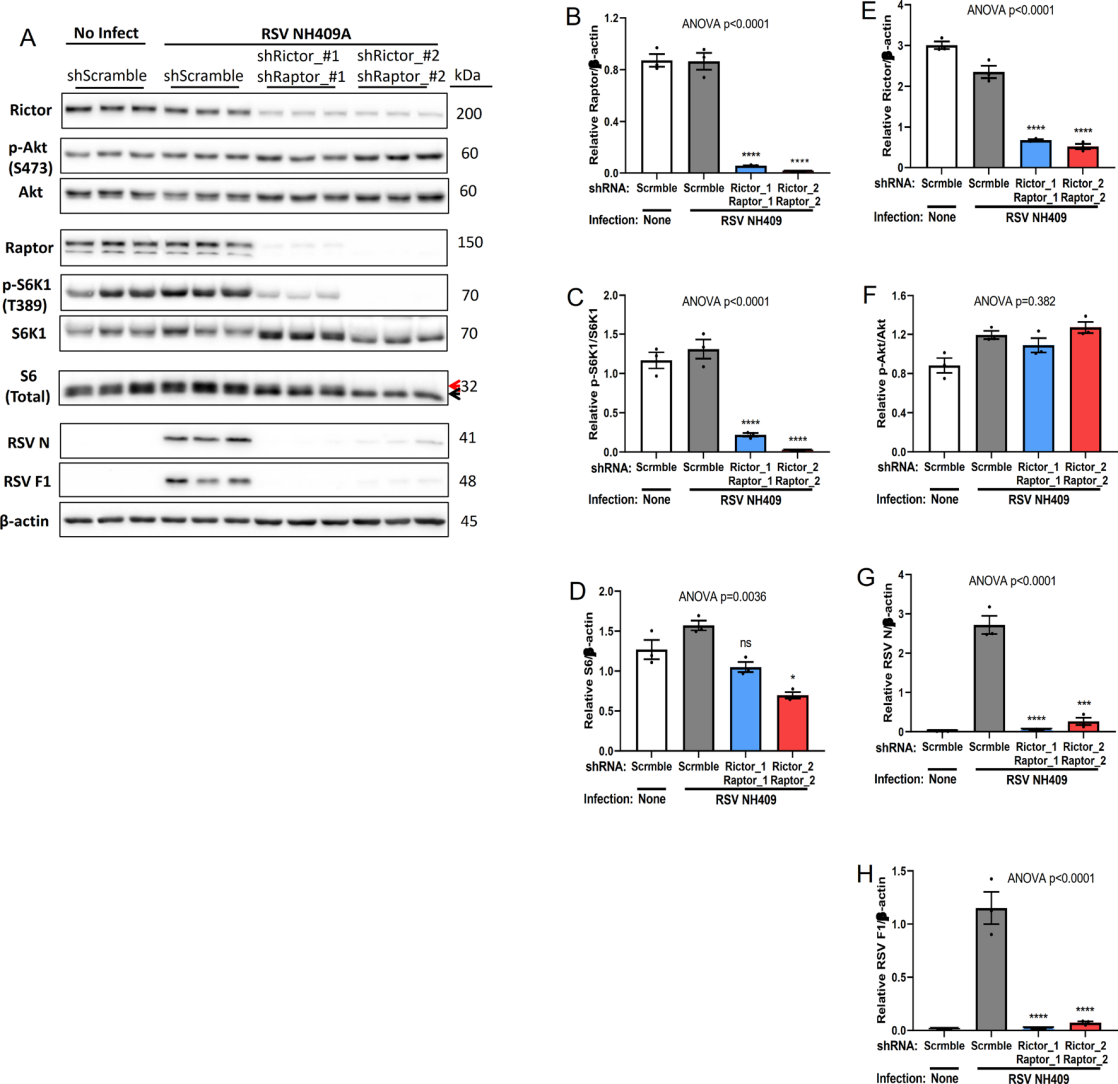
Simultaneously knockdown both Raptor and Rictor block viral protein synthesis. Lenti-viral short-hairpin RNAs (shRNA) were used to knockdown expression of both Raptor and Rictor in A549 cells. Double shRaptor/shRictor-A549 cells were infected with RSV NH409A. Cellular and viral protein expression was measured at 24 h post infection (m.o.i = 0.2). Asterisk (*) or non-significant (ns) is compared to Scramble/RSV control. (**A**) Western blot analysis of cellular and protein expression. (**B**–**H**) Quantification of protein synthesis displayed in panel A (n = 3). (**B**) Raptor/β-actin; (**C**) phospho-S6K1/S6K1; (**D**) total ribosomal protein S6/β-actin; (**E**) Rictor/β-actin; (**F**) phospho-Akt/Akt; (**G**) RSV nucleoprotein N/β-actin; (**H**) RSV fusion protein F1/β-actin. Error bars, SEM; *, *p* < 0.05; **, *p* < 0.01; ***, *p* < 0.001; ****, *p* < 0.0001; *n.s.* non-significant.

Given the redundancy of complex 1 and 2 activities during the RSV replication cycle, one would predict that knocking down both Raptor and Rictor simultaneously would impede viral protein synthesis (Fig. 4A). Knockdown of Raptor in double shRictor/shRaptor cells abolished S6K1 activation, and reduced S6 phosphorylation (Fig. 4B–D), whereas, knockdown of Rictor in double shRictor/shRaptor cells does not have substantial impact on Akt phosphorylation (Fig. 4E,F), possibly due to a more complete knock down of Raptor than Rictor, which may resulted feed-back activation of Akt. However, knocking down both Rictor and Raptor within same cells abolished viral protein synthesis (Fig. 4G,H).

### Knockdown of mTOR kinase blocks viral protein synthesis

**Figure 5:**
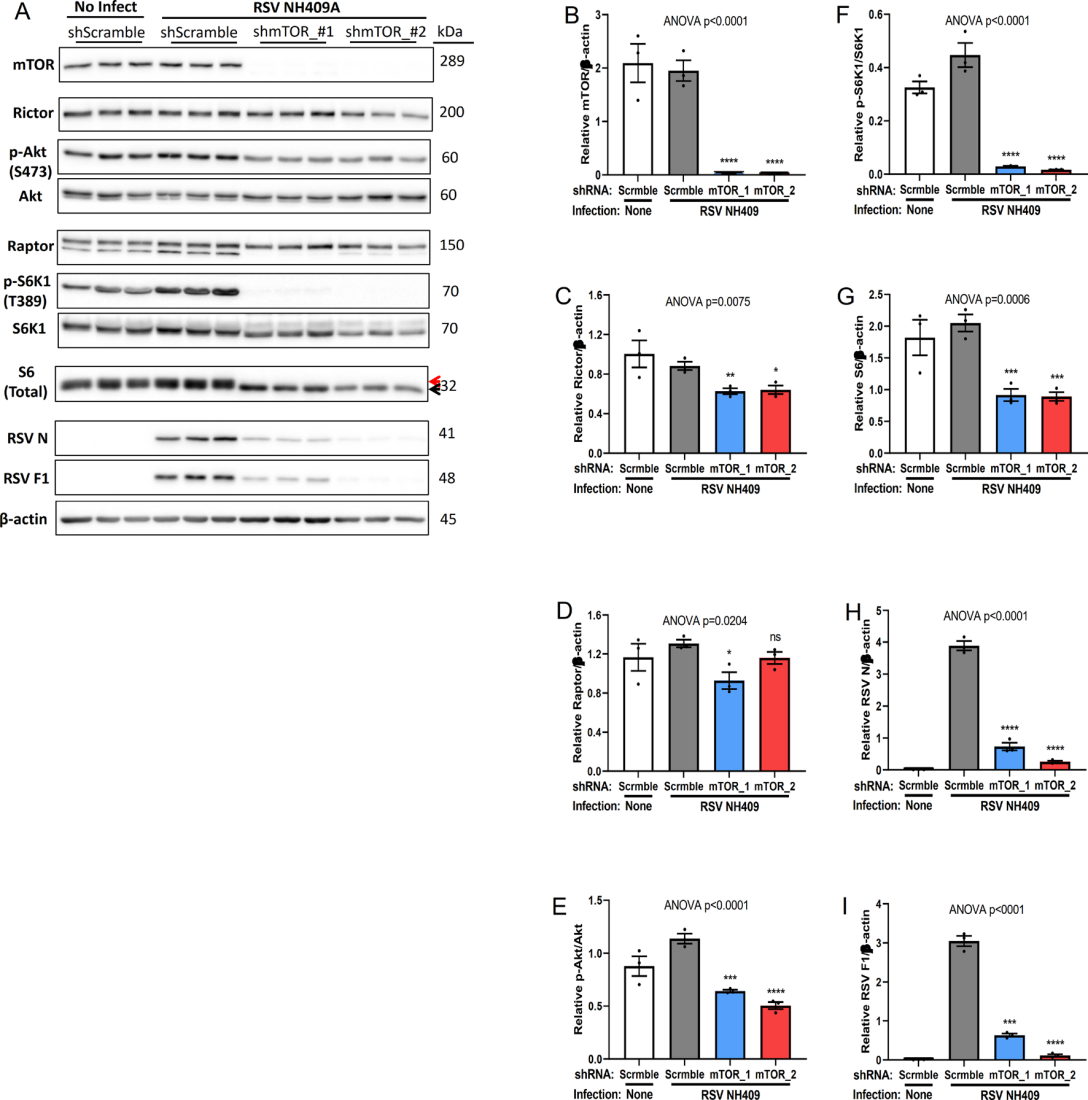
Genetic knockdown of mTOR kinase blocks viral protein synthesis. Lenti-viral short-hairpin RNAs (shRNA) were used to knockdown expression of mTOR in A549 cells. shmTOR-A549 cells were infected with RSV NH409A. Cellular and viral protein expression was measures at 24 h post infection (m.o.i = 0.2). Asterisk (*) or non-significant (ns) is compared to Scramble/RSV control. (**A**) Western blot analysis of cellular and protein expression. (**B**–**I**) Quantification of protein synthesis displayed in panel A (n = 3). (**B**) mTOR/β-actin; (**C**) Rictor/β-actin; (**D**) Raptor/β-actin; (**E**) phospho-Akt/Akt; (**F**) phospho-S6K1/S6K1; (**G**) total ribosomal protein S6/β-actin; (**H**) RSV nucleoprotein N/β-actin; (**I**) RSV fusion protein F1/β-actin. Error bars, SEM; *, *p* < 0.05; **, *p* < 0.01; ***, *p* < 0.001; ****, *p* < 0.0001; *n.s.* non-significant.

mTORC1 is well-known to regulate cell growth and metabolism, while mTORC2 controls proliferation and survival^19^. Although they have different biological functions, these two distinct complexes shared the same mTOR kinase. To eliminate the possibility of differentially reduced levels of Raptor and Rictor in the gene knockdown experiment and a possible associated feed-back activation (Fig. 4), mTOR was genetically knocked down by stable lenti-viral short-hairpin RNA (shRNA) (Fig. 5A,B)^37^. Predictably, the knockdown of mTOR had minimal, though significant effect on Rictor and Raptor levels (Fig. 5C,D), while there was significantly reduced Akt and S6K1 activation, and S6 phosphorylation (Fig. 5E–G). Reduction of mTOR protein nearly abolished viral protein synthesis (Fig. 5H,I), replicating the results seen in double Rictor/Raptor knockdown (Fig. 4).Collectively, our data demonstrated the redundancy impact of each mTOR complex for the generation of infectious progeny virus and the necessity of mTOR protein for viral protein expression.

### Pharmacological interference of the function of mTOR complexes reduces the generation of RSV progeny virus

**Figure 6:**
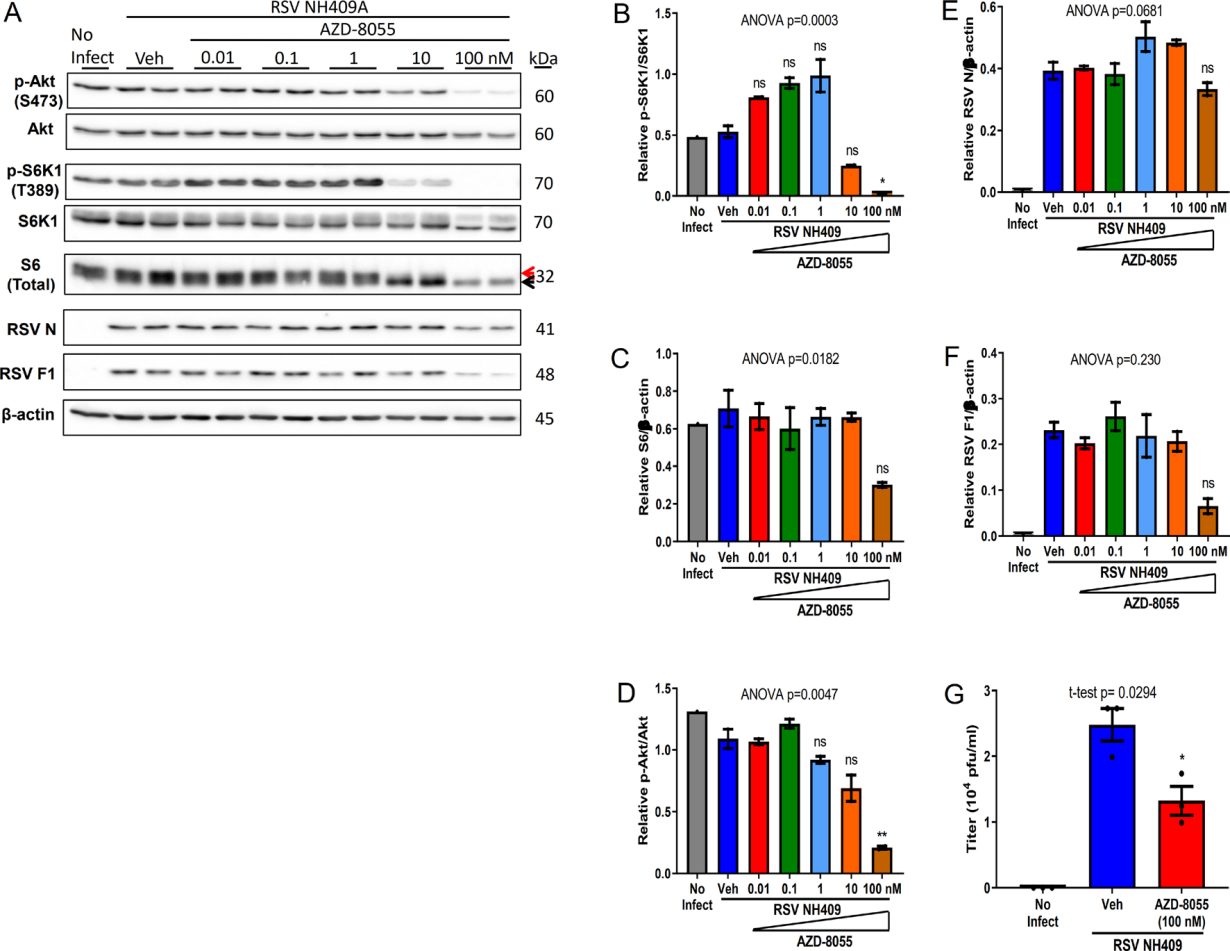
Inhibition of both mTORC1 and mTORC2 by AZD-8055 reduces the generation of infectious progeny virus. A549 cells were infected with clinical isolate NH409A and subsequently treated with varying concentrations of AZD-8055, an inhibitor of both mTORC1 and mTORC2. Protein analyses and the generation of infectious progeny virus was measured at 24 h post infection (m.o.i = 0.2). Asterisk (*) or non-significant (ns) is compared to vehicle (Veh) control. (**A**) Western blot of cellular and viral proteins. The 2 forms of S6 are designated by the red and black arrows. (**B**–**F**) Quantification of cellular and viral proteins displayed in A (n = 2). (**B**) phospho-S6K1/S6K1; (**C**) total ribosomal protein S6/β-actin; (**D**) phospho-Akt/Akt; (**E**) RSV nucleoprotein N/β-actin; (**F**) RSV fusion protein F1/β-actin. (**G**) Quantification of virus progeny in the absence and presence of AZD-8055 (n = 3). Error bars, SEM; *, *p* < 0.05; **, *p* < 0.01; ***, *p* < 0.001; ****, *p* < 0.0001; *n.s.* non-significant.

Although mTORC1 is the focal point of mTOR research, mTORC2 is emerging as an important complex in many cancers^38,39^. While there have been drugs developed to specifically target either mTORC1 alone or both mTORC1 and mTORC2 complexes, drugs specific for mTORC2 have not yet been developed or validated for clinical use. To confirm the observations of mTOR knockdown (Fig. 5) and potential therapeutic use, infected A549 cells were treated with dual complexes inhibitor AZD-8055, an ATP-competitive inhibitor of mTOR^40^. Consistent with rapamycin, AZD-8055 treatment reduced phosphorylation of S6K1 and S6 (Fig. 6A–C). In contrast to rapamycin however, AZD-8055 also reduced phosphorylation of Akt (Fig. 6D). Provocatively, at 100 nM, AZD-8055 significantly reduced the generation of infectious progeny virus (Fig. 6E–G). Similar observations could be seen with Torin-1, another ATP-competitive inhibitor of mTOR^41^ (Supplementary Fig. 6). Our data suggesting mTOR kinase is a novel therapeutic target for RSV infection.

### mTOR inhibitors block human coronavirus OC43 transcription and protein synthesis

**Figure 7:**
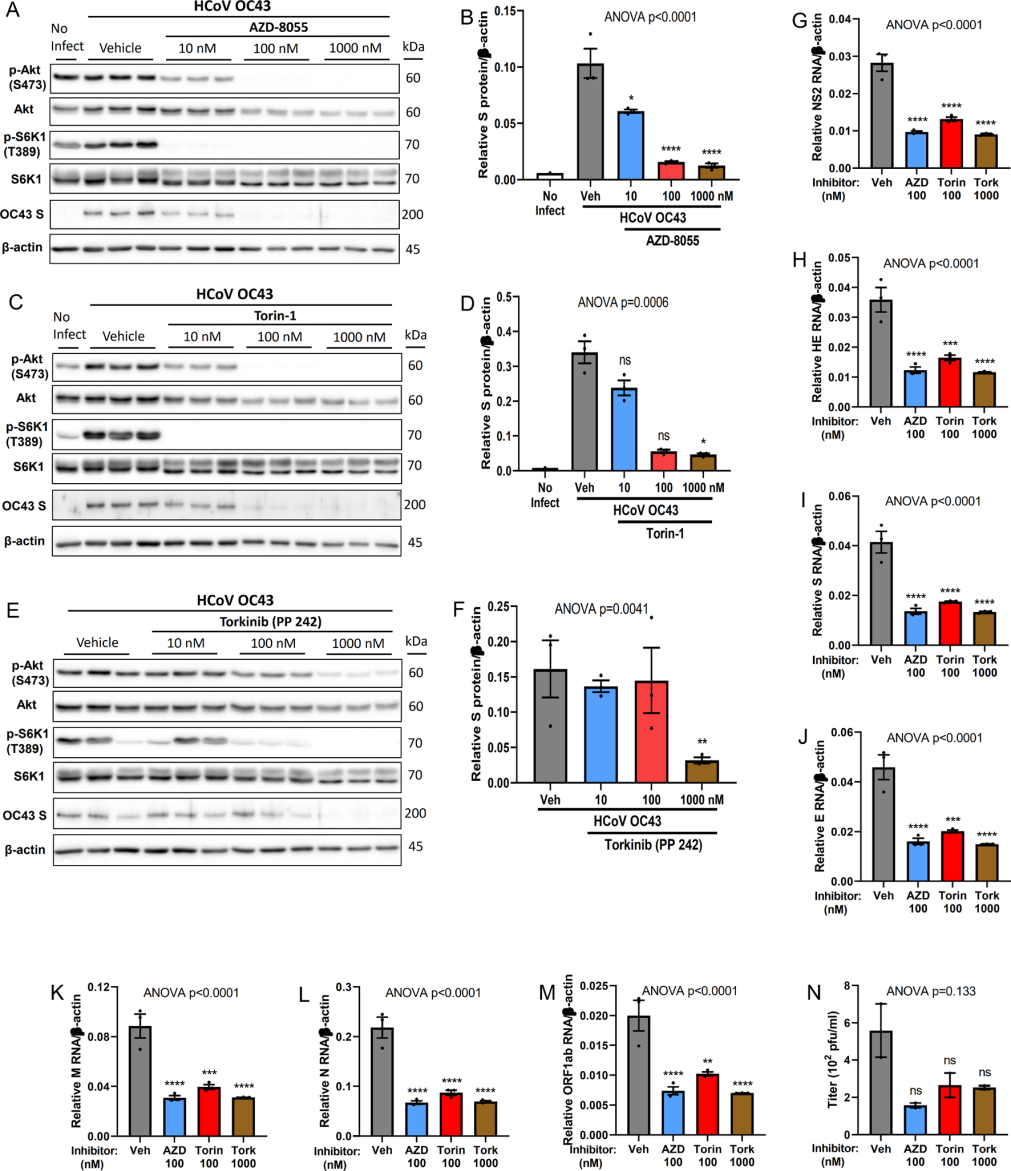
mTOR inhibitors nearly abolish human coronavirus OC43 transcription, protein synthesis and generation of infectious progeny virus. A549 cells were infected with human coronavirus (HCoV) OC43 for 1.5 h and subsequently treated with different mTOR inhibitors, AZD-8055, Torin-1, or Torkinib, at varying concentrations. Protein (m.o.i = 1), and viral RNA (m.o.i = 0.3) analyses were measured at 24 h post infection. Asterisk (*) or non-significant (ns) is compared to vehicle (Veh) control. (**A**) Western blot of cellular and viral proteins at presence of AZD-8055. (**B**) Quantification of viral protein spike S displayed in A (n = 3). **(C**) Western blot of cellular and viral proteins at presence of Torin-1. (**D**) Quantification of viral protein spike S displayed in C (n = 3). **(E**) Western blot of cellular and viral proteins at presence of Torkinib. (**F**) Quantification of viral protein spike S displayed in E (n = 3). (**G**–**M**) Quantification of viral RNA expression at presence of indicated concentration of mTOR inhibitors (n = 3). (**G**) non-structural protein 2 NS2; (**H**) hemagglutinin-esterase HE; (**I**) spike surface glycoprotein S; (**J**) envelope protein E; (**K**) membrane protein M; (**L**) nucleocapsid protein N; (**M**) replicase polyprotein ORF1ab. (**N**) TCID_50_ quantification of virus progeny at presence of indicated concentration of mTOR inhibitors (n = 2). Error bars, SEM; *, *p* < 0.05; **, *p* < 0.01; ***, *p* < 0.001; ****, *p* < 0.0001; *n.s.* non-significant.

To test whether inhibiting the function of mTOR complexes would have any negative impact on the processes involved in the viral replication cycle of beta-coronavirus, OC43-infected A549 cells were treated with various mTOR inhibitors (Fig. 7). At 100 nM, both AZD-8055 (Fig. 7A,B) and Torin-1 (Fig. 7C,D) nearly abolished HCoV OC43 spike S protein synthesis. Similar observations could be seen at 1 µM of Torkinib (PP 242), an ATP-competitive mTOR inhibitor (Fig. 7E,F)^42,43^. Inhibition of both Akt and S6K1 activation (phosphorylated) correlated with the near abolishment of S protein synthesis. The near complete inhibition of viral protein S correlated with a reduction in RNA expression, which was consistent across many viral genes (Fig. 7G–M, and Supplementary Fig. 7A–G). Generation of infectious progeny virus was also diminished (Fig. 7N, and Supplementary Fig. 8A,B). Together, our data showed that beta-coronavirus viral replication functions, like RSV, were inhibited by mTOR kinase inhibitors, suggesting a potential treatment for SARS-CoV-1, MERS-CoV, and SARS-CoV-2.

## Discussion

Our studies further uncovered the biological importance of host/pathogen interactions during RSV infection, specifically viral protein synthesis and the generation of infectious progeny virus. Here, our data demonstrated that limiting either mTORC1 or mTORC2 signaling enhanced viral protein production and the generation of infectious progeny virus, while inhibiting both complexes impeded RSV protein synthesis, thereby establishing the requirement of mTOR signaling for productive RSV infection. Additionally, our studies highlighted the challenge of RSV infection in patients receiving chemotherapy or immunosuppressive agents (e.g. transplant recipients) such as mTOR inhibitors as we have shown that these commonly used chemotherapeutic agents enhance RSV protein expression and the generation of infectious progeny virus in cell culture. Our observations are consistent with a previous study that showed that rapamycin increased RSV RNA in infected dendritic cells^44^, demonstrating that this phenomenon occurs in primary cells and supports the notion that the use of standard cell lines, as used in our studies, can result in insights into pathogenesis. Moreover, a recent study showed that inhibition of mTORC1 by rapamycin enhanced autophagy and was associated with an increase of RSV replication in HEp-2 cells^45^, supporting our findings.

Rapamycin binds FKBP12 protein to form an mTORC1-specific inhibitor^28–30^. Rapamycin-FKBP12 complexes do not bind or inhibit mTORC2; however, prolonged rapamycin treatment does abrogate mTORC2 signaling, likely due to the inability of rapamycin-bound mTOR to incorporate into new mTORC2 complexes^46,47^. Consistent with this notion, high concentration of rapamycin treatment diminished or attenuated Akt phosphorylation (Supplementary Fig. 2). Our data also showed significant induction of viral proteins and the production of infectious progeny virus at high doses of rapamycin (10–100 nM), which is equivalent to 9.14–91.4 ng/ml. The target blood concentration of sirolimus, the FDA approved version of rapamycin, is 10–24 ng/ml^48–50^; therefore, individuals treated with sirolimus for immunosuppression (e.g. solid organ transplant recipients) or other indications may be at significant risk if they were to contract RSV infection.

Our data showed rapamycin increased the generation of infectious progeny virus, correlated with elevated Akt phosphorylation (Fig. 1) and was confirmed by genetic knockdown of Raptor (Fig. 3 & Supplementary Fig. 5), an indication of active mTORC2 signaling. Unfortunately, there are no validated drugs available to inhibit mTORC2 specifically. It is well known that mTORC2 is upstream of mTORC1, and mTORC1 negatively regulates autophagy^19^. By inhibiting both complexes at once via genetic knockdown of mTOR, one would expect to enhance autophagy^51^. If that were the case, then viral protein expression or production of progeny virus should be increased. In contrast to our expectation, inhibiting both complexes with genetic knockdown of both Rictor and Raptor (Fig. 4) or mTOR (Fig. 5), confirmed by mTOR inhibitor AZD-8055 (Fig. 6) decreased the generation of progeny virus. These observations are also in line with literature that autophagy is well known for its ability to limit replication of intracellular pathogens^52^.

The inhibition of mTORC1 with rapamycin induced activation of mTORC2 (Akt phosphorylation) via the loss of negative feedback loops (Fig. 1 & Supplementary Fig. 2)^31,32^. In line with those observations, genetic knockdown of Raptor (mTORC1) increased Akt phosphorylation (Fig. 3 & Supplementary Fig. 5). Although mTORC1 is known to be a downstream target of mTORC2 (Fig. 7G), one would expected that inhibition of mTORC2 function would diminish mTORC1 (S6K1 phosphorylation) signaling. However, knockdown of Rictor (mTORC2) resulted in the increase of S6K1 phosphorylation. Previous studies have shown that mTORC2 appears to be necessary for Akt activity toward some but not all substrates^37,53–56^, suggesting the hyper-activation of S6K1 is possibly due to an unknown feedback mechanism. Knockdown of either Raptor or Rictor resulted in increased viral proteins, suggesting the mTOR complexes are redundant for RSV replication. To our knowledge, this is the first study to report this observation.

The first mTORC2 substrate identified was PKCα^57,58^. Recently, more mTORC2 targets were identified, including PKCδ, PKCζ, PKCγ, and PKCε^59–61^. More importantly, mTORC2 is well known for phosphorylation and activation of Akt^37^. Akt is known to promote cell survival, proliferation, and growth, and is involved in many cancers^19,35^. Treatment with pan-Akt inhibitor MK-2206 modestly enhanced the generation of progeny virus (Fig. 2). Inhibition of Akt resulted slightly decreased of S6K1 phosphorylation (less active mTORC1), which possibly induced activation of mTORC2 via the loss of negative feedback loops. Our observations with MK-2206 have implications for other classes of Akt inhibitors, specifically for those individuals receiving related compounds for chemotherapy or immunosuppression who may also encounter RSV.

In light of the current SARS-CoV-2 pandemic, we turned to explore the effects of targeting cellular functions on the replication cycle of human coronaviruses, specifically viral transcription, protein synthesis and generation of progeny virus. Effective prevention of COVID-19 requires multifaceted efforts, including vaccinations and antiviral drugs. As of this writing, there are at least 6 vaccines that are in use worldwide. These vaccines target the spike glycoprotein as neutralizing antibodies against this viral protein can prevent infection. However, it is now clear that mutations in the spike protein have emerged that alter its antigenic structure, potentially limiting the specificity of the vaccine-induced immunity^62,63^. Further, there is concern that the new variants identified in the United Kingdom, South Africa, Brazil, and elsewhere have acquired the necessary mutations to make the vaccine-induced neutralizing antibodies less effective^64^. There are a number of studies that sought to determine whether any preexisting antiviral drugs have sufficient effectiveness for treating COVID-19 patients^65,66^. On May 1, 2020, remdesivir, adenosine nucleotide triphosphate analog, was authorized by U. S. Food and Drug Administration for compassionate use in the United States^67^. Due to the fact that SARS-CoV-2 is prone to mutation, one foreseeable obstacle for antiviral therapy is the development of viral resistance, as viral genomic mutations occur during viral replication^17,18^. Indeed, mutations in the RNA replicase of mouse hepatitis virus, a coronavirus, caused partial resistance to remdesivir^68^. Therefore, alternative therapeutic approaches should be considered. Once inside the host, coronaviruses utilize the host machinery for their replication. Here, we show that targeting mTOR kinase with various small molecule inhibitors is a means to inhibit or diminish viral protein synthesis or replication as we have learned from our RSV experiments. In parallel to our findings with RSV, treatment with mTOR inhibitors abolished or diminished HCoV OC43 spike protein synthesis and the generation of infectious progeny virus, providing a proof-of-concept for alternative therapeutics.

**Figure 8:**
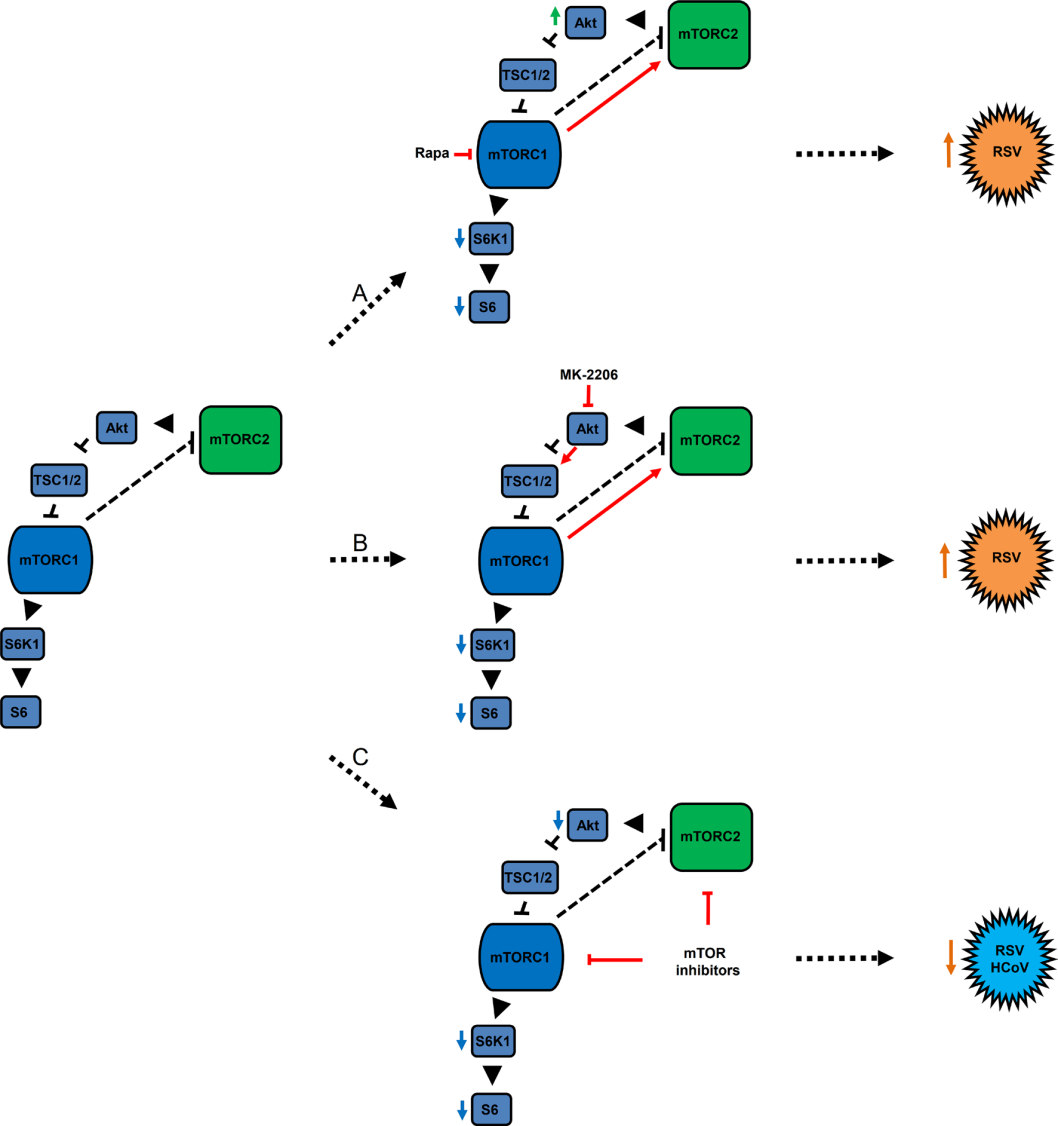
Schematic inhibition of RSV and HCoV with mTOR inhibitors. Simple illustration is adopted from a previous review^19^. The left portion of the figure delineates the known pathways and interactions between mTORC1 and mTOTC2. (**A**) Rapamycin (Rapa), a mTORC1-specific inhibitor, increases RSV viral protein synthesis and production of viral progenies. Specific inhibition of mTORC1 by Rapamycin deactivates (de-phosphorylation) of S6K1 and S6 (as denoted by the downward blue arrows). This results in negative feed-back activation (phosphorylation) of mTORC2 (red arrow) and an increase in the phosphorylation of Akt (denoted by the upward green arrow). Data supporting this model is presented in Fig. 1. (**B**) Akt inhibitor MK-2206 increases RSV viral proteins and progenies. Inhibition of Akt phosphorylation activates TSC1/2 (red arrow), which deactivates mTORC1 signaling (blue arrows). This decrease of mTORC1 signaling presumably activated mTORC2 (red arrow) in line with the observed activity of rapamycin. Data supporting this model is presented in Fig. 2. (**C**) mTOR inhibitors block essential functions of HCoV OC43 and RSV replication. Inhibition of both mTORC1 and mTORC2 results in decreased phosphorylation of Akt, S6K1 and S6 (blue arrows) ultimately leading to a reduction of protein synthesis and production of progeny virus of RSV (AZD-8055, see Fig. 6) and reduction of viral gene transcription and protein synthesis of human coronavirus OC43 (AZD-8055 and Torin-1, see Fig. 7). Key: ⊢  = inhibition (de-phosphorylation), ► = activation (phosphorylation).

In summary (Fig. 8), the significance and novelty of our current study include the following: (1) dynamic utilization of mTOR signaling by RSV for its protein synthesis and generation of infectious progeny virus, established by pharmacologic and genetic models; (2) redundancy of mTOR complexes during the RSV replication cycle, a novel finding; (3) potential impact of mTOR and Akt inhibitors on RSV protein synthesis and generation of infectious progeny virus and the danger to cancer patients and others who are receiving these drugs should they become infected with RSV or any of the beta-coronaviruses and; (4) a proof-of-concept for potential therapeutic treatment for SARS-CoV-1, MERS-CoV and SARS-CoV-2. In all, the targeting of host factors may be a useful therapeutic strategy for other RNA viruses (such as the parainfluenza viruses) as well as existing or emerging human coronaviruses.

## Experimental procedures

### Virus and cells

RSV clinical isolates were obtained from RSV-infected individuals, as described previously, in New Haven, CT^22^ and Dallas, TX^20^. Collection and use of clinical isolates followed all institutional requirements and guidelines, were consistent with policies and regulations for the use of patient derived materials and approved by the respective Institutional Review Boards. The need to obtain informed consent was waived by the Institutional Review Board (IRB) of Yale University and the University of Texas Southwestern Medical Center. Isolates were plaque-purified and concentrated by HEp-2 cells. Plaque titration and working stocks prepared by 0.5% methylcellulose overlays of infected A549 cells for 3 days before being stained as described previously^20^. Viral stocks were prepared with a low inoculum with multiplicity of infection (m.o.i) of 0.01 to minimize the production of defective interfering (DI) particles. Further, the number of passages of virus in cell culture was limited to prevent the potential viral adaption to cell culture. For all experiments, a m.o.i of 0.2 was used unless otherwise stated. All specimens from which virus were obtained were submitted as part of routine care. Only left-over material was used for viral propagation. Collection of specimens from the Clinical Virology Laboratory at Yale-New Haven Hospital was approved by the Yale University Human Investigations Committee. The RSV isolates from Dallas, Texas were propagated from a de-identified clinical specimen obtained from the Clinical Microbiology Laboratory at Children's Medical Center, Dallas. Collection and use of clinical isolates followed all institutional requirements and guidelines and was consistent with policies and regulations for the use of patient derived materials. Human coronavirus (HCoV) OC43 (ATCC VR-1558), and human HCT-8 colon cells (ATCC CCL-244) were obtained from American Type Culture Collection (Manassas, VA).

A549 (CCL-185) cells were obtained from ATCC and cultivated in F-12 Kaighn’s modification media with 10% fetal bovine serum. Viral titers were determined using a plaque assay^20^. Plaques were detected with an immunohistochemical staining technique with a primary anti-RSV Fusion protein antibody (palivizumab, MedImmune, Gaithersberg, MD) and a secondary HRP conjugated anti-human antibody (Jackson ImmunoResearch Laboratories, Inc., West grove, PA). HCoV OC43 viral titer was determined with HCT-8 cells by median tissue culture infectious dose (TCID_50_), according to ATCC protocol and briefly described elsewhere^69^.

### Chemicals, shRNA plasmids, and antibodies

Rapamycin (LC Laboratories); MK-2206 HCl (Cayman Chemical); AZD-8055, Torin-1, and Torkinib (PP 242) (Medchemexpress) were dissolved in DMSO as manufacturer instructions. Equal volume of DMSO served as vehicle (veh).

Scramble shRNA (1864), Raptor_1 shRNA (1857), Raptor_2 shRNA (1858), Rictor_1 shRNA (1853), Rictor_2 shRNA (1854), mTOR_1 shRNA (1855), mTOR_2 shRNA (1856)^37^ were packaged by lentiviral plasmids pMD2.G (12259) psPAX2 (12260), gifted to Addgene by Didier Trono. All of the above plasmids were purchase from Addgene for research purposes. Briefly, shRNA plasmid together with pMD2.G and psPAX2 were transfected into 293 T cells. Media supernatants which contained viruses were collected after 72 h of transfection, and used to infect A549 cells. Infected-A549 cells were screened with puromycin (1.5 µg/mL) after 3 days of infection. Western blots were performed to evaluate protein expression. All western blots/images are pre-cut.

The following antibodies were used: primary rabbit Phospho-Akt (S473), Akt, phospho-S6K1 (T389), S6K1, S6, mTOR, β-actin antibodies (Cell Signal Technology); rabbit Rictor and Raptor antibodies (Bethyl Laboratories); mouse RSV N antibody (Novus Biologicals); human RSV F1 (palivizumab) antibody (MedImmune); rabbit coronavirus spike antibody (Invitrogen PA581777) were detected with secondary goat anti-rabbit HRP antibody (Thermo Fisher Scientific), horse anti-mouse HRP antibody (Cell Signal Technology), or rabbit anti-human HRP antibody (Jackson ImmunoResearch Laboratories).

### RSV and HCoV OC43 infection and protein detection

Overall, 2 × 10^5^ cells per well of a 12-well plate of A549 cells were infected, m.o.i. of 0.2, with sucrose-purified clinical isolates of RSV. After 90 min of infection, the inoculum was removed, the cells were washed with serum free media, and fresh F-12 Kaighn's modification media containing 5% FBS with or without indicated drugs was added to the infected monolayers. The cells were directly lysed with laemmli sample buffer 24 h post-infection. Western blotting was performed with SDS-PAGE gels. Chemiluminescence signals were captured by GE ImageQuant LAS-4000. Relative protein quantifications were analyzed by ImageJ^70^. A549 cells were infected with HCoV OC43 at m.o.i. of 1, and western blotting was performed with SDS-PAGE gels 24 h post-infection. All western blots/images are pre-cut.

### RNA extraction, reverse transcription and quantitative PCR

A549 cells were infected with HCoV OC43 at m.o.i. of 0.3. Total RNAs were extracted at 24 h post-infection by TRIzol reagent (Invitrogen). First-strand of total cDNAs were synthesized with random primers by high capacity cDNA reverse transcription kit (Applied Biosystems). Quantitative PCR (qRT-PCR) was performed with PowerUp SYBR green (Applied Biosystems) and CFX96 thermal cycler (Bio-rad Laboratories).

### Statistical analyses

The biological data was log-transformed to obtain normal distribution. Shapiro–Wilk test was used to test normality. Brown-Forsythe test was used to test equal variances among different groups. When the log-transformed data do not pass the normality test, (1) Wilcoxon Rank Sum test was used to test difference in median between two groups; (2) Kruskal–Wallis test was performed to test the difference in median among 3 or more groups. Dunn’s method was used to adjust p value for multiple comparisons between test groups and control group (Vehicle or Scramble). When the log-transformed data pass the normality test, (1) two-sample t-test was used to test difference in mean between two groups; (2a) standard one-way ANOVA was performed to test difference in mean among 3 or more groups if data also pass equal variance test; (2b) Welch’s one-way ANOVA was used to test difference in mean among 3 or more groups if data do not pass equal variance test. Dunnett method was used to adjust p value for multiple comparisons between test groups and control group (Vehicle or Scrmble). The adjusted p values were reported for multiple comparisons. The data were displayed in original scale (without transformation) in figures. Biological data for “No infection” were presented in the figures, but they were not included for statistical tests. All statistical analyses were performed by Graphpad Prism 9.2.0 software. The significance levels were designated as * for *p* < 0.05, ** for *p* < 0.01, *** for *p* < 0.001, **** for *p* < 0.0001 and n.s. for nonsignificant (*p* > 0.05).

## Supplementary Information

Supplementary Figures.
