# Supplementary material for: mTOR kinase is a therapeutic target for respiratory syncytial virus and coronaviruses
Source: Sci Rep. 2021 Dec 24;11:24442. doi: 10.1038/s41598-021-03814-7 (PMC8709853; doi:10.1038/s41598-021-03814-7)

Supplementary Figure 1 **Huynh et al**

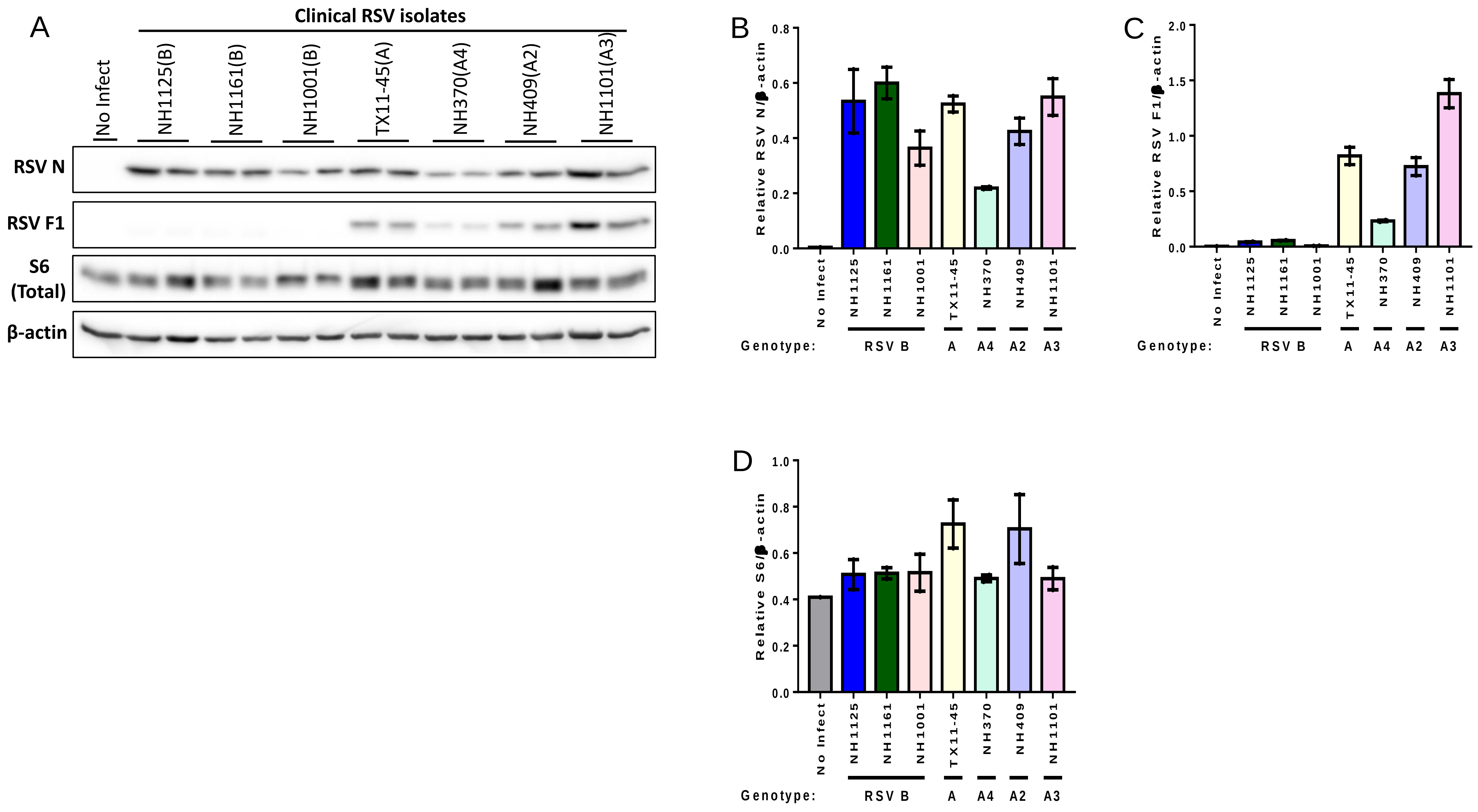

Fig. S1. Viral protein expression analyses of various clinical RSV isolates.

A549 cells were infected with m.o.i of 1 of various clinical isolates.
(**A**) Western blot analyses of cellular and viral proteins at 24 hours post-infection.
(**B-D**) Quantification of proteins displayed in A (n = 2).
(**B**) RSV nucleoprotein N/β-actin; (**C**) RSV fusion protein F1/β-actin; (**D**) total ribosomal protein S6/β-actin.

Supplementary Figure 2

Huynh et al

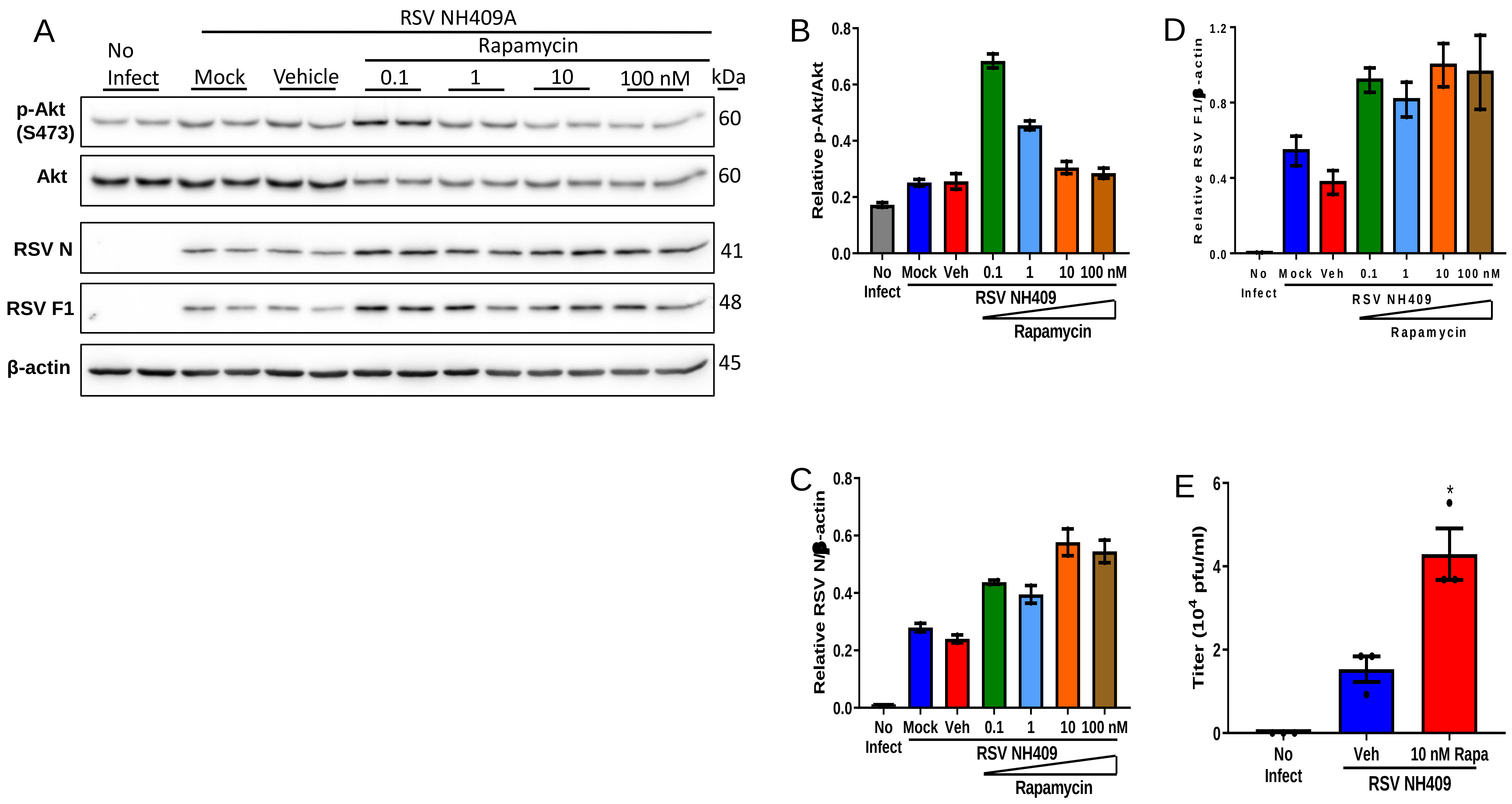

Fig. S2. Additional analyses of rapamycin treatment and RSV NH409A replication.

A549 cells were infected with clinical isolate NH409A and subsequently treated with varying concentrations of rapamycin. Protein analyses and viral replication was measured at 24 hours post infection (m.o.i = 0.2)

(**A**) Western blot of cellular and viral proteins.

(**B-D**) Quantification of proteins displayed in A (n = 2).

(**B**) phospho-Akt/Akt; (**C**) RSV nucleoprotein N/β-actin; (**D**) RSV fusion protein F1/β-actin.

(**E**) Quantification of progeny virus in the absence and presence of 10 nM rapamycin (n = 3).

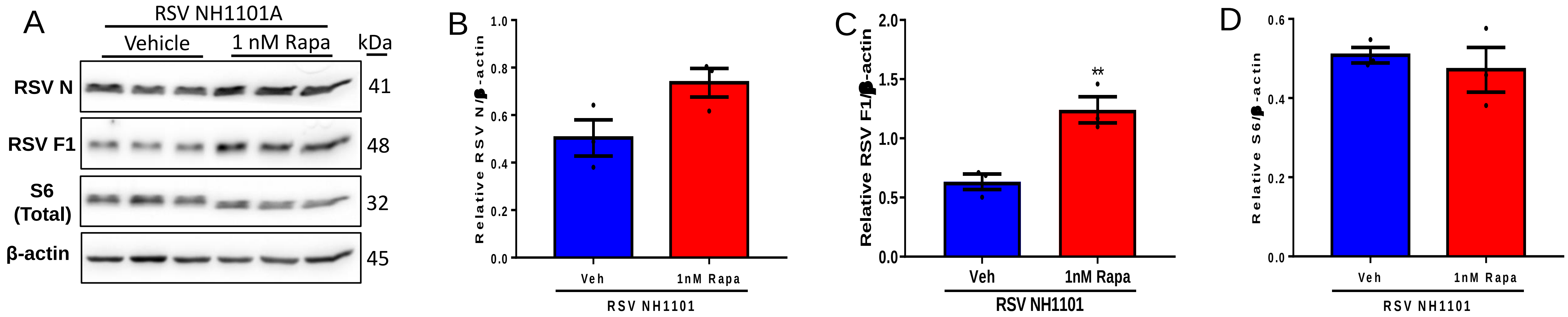

Fig. S3. Rapamycin enhances viral protein expression of clinical strain NH1101, an isolate of the A3 subgroup of RSV.

A549 cells were infected with clinical isolate NH1101A and subsequently treated with 1 nM rapamycin. Protein analyses at 24 hours post infection (m.o.i = 1).
(**A**) Western blot of cellular and viral proteins.
(**B-D**) Quantification of proteins displayed in A (n = 3).
(**B**) RSV nucleoprotein N/β-actin; (**C**) RSV fusion protein F1/β-actin; (**D**) total ribosomal protein S6/β-actin.

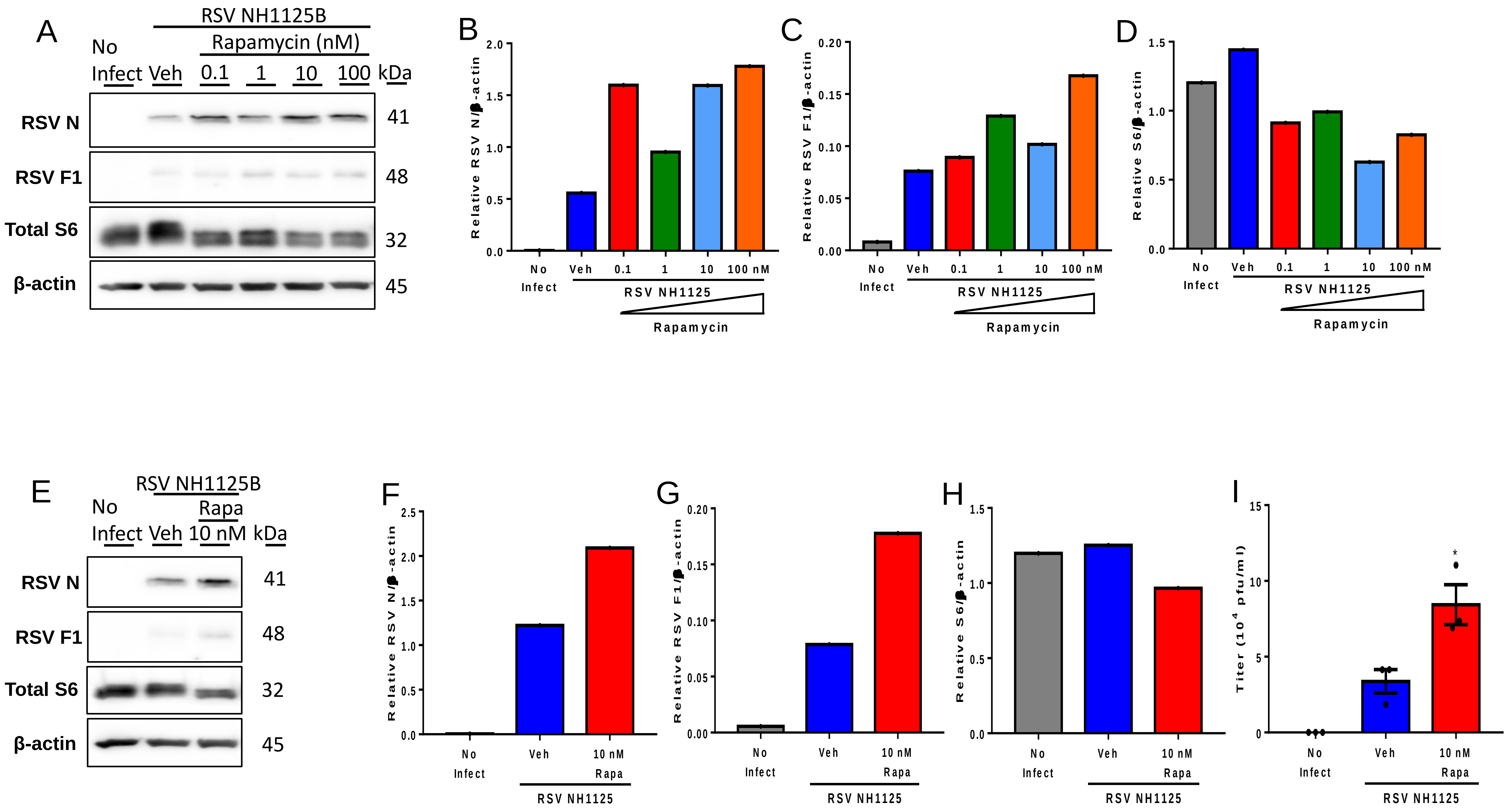

Fig. S4. Rapamycin enhances viral protein expression and viral replication of clinical strain NH1125, an isolate of the B subgroup of RSV.

A549 cells were infected with clinical isolate NH1125B and subsequently treated with varying concentrations of rapamycin. Protein analyses and viral replication was measured at 24 hours post infection (m.o.i = 0.2).

(**A**) Western blot of cellular and viral proteins.

(**B-D**) Quantification of proteins displayed in A.

(**B**) RSV nucleoprotein N/β-actin; (**C**) RSV fusion protein F1/β-actin; (**D**) total ribosomal protein S6/β-actin.

(**E**) Western blot of cellular and viral proteins at 10 nM rapamycin.

(**F-H**) Quantification of proteins displayed in E.

(**F**) RSV nucleoprotein N/β-actin; (**G**) RSV fusion protein F1/β-actin; (**H**) total ribosomal protein S6/β-actin.

(**I**) Quantification of viral replication in the presence of 10 nM rapamycin (n = 3).

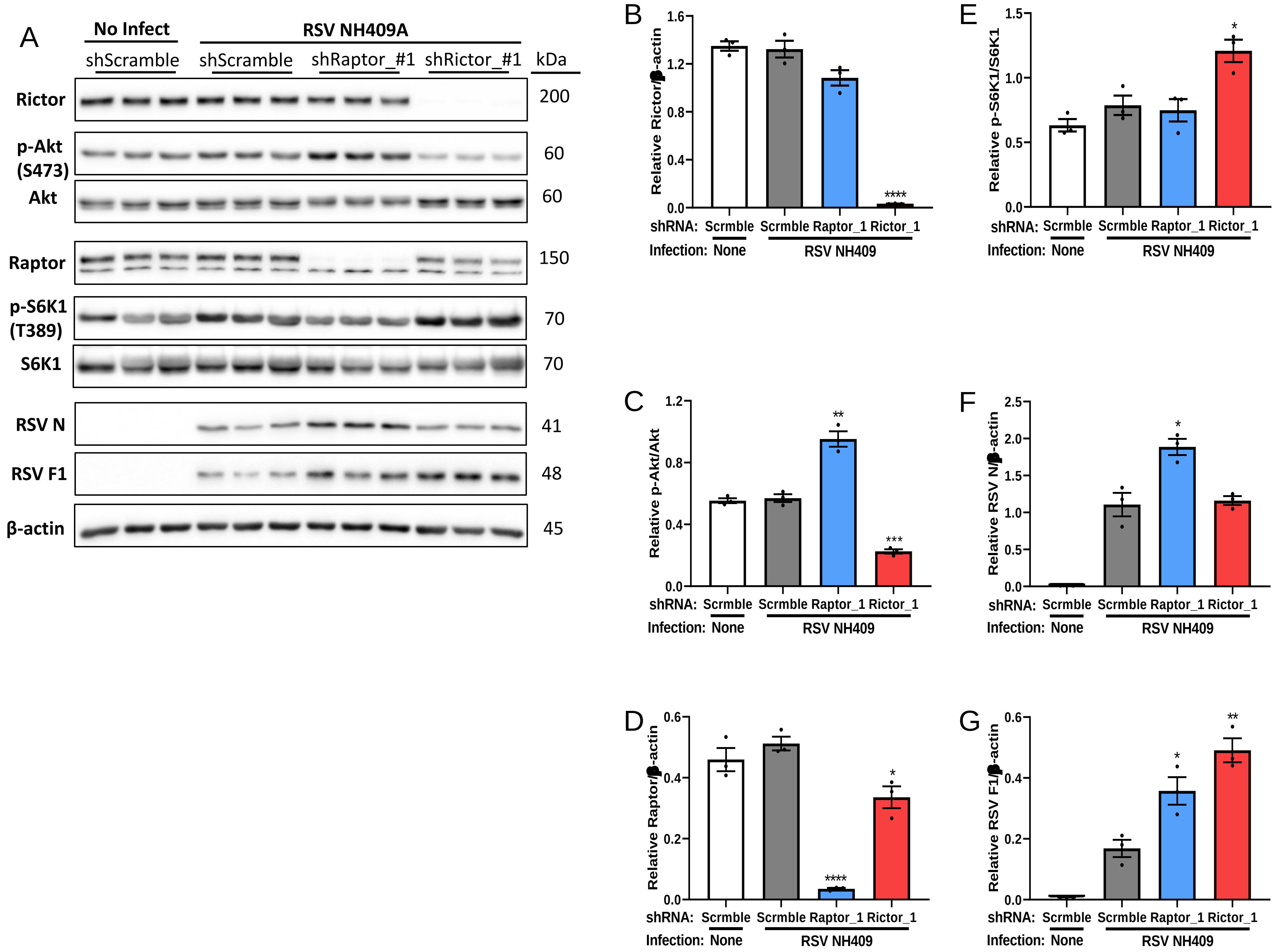

Fig. S5. Additional analyses of genetic knockdown of Raptor and Rictor on RSV NH409A protein synthesis.

Lenti-viral short-hairpin RNA (shRNA) were used to knock down expression of either Raptor or Rictor in A549 cells. shRaptor- or shRictor-A549 cells were infected with RSV NH409A. Cellular and viral protein expression was measures at 24 hour post infection (m.o.i = 0.2).

(**A**) Western blot of cellular and viral proteins.

(**B-G**) Quantification of protein displayed in A (n = 3).

(**B**) Rictor/β-actin; (**C**) phospho-Akt/Akt; (**D**) Raptor/β-actin; (**E**) phospho-S6K1/S6K1; (**F**) RSV nucleoprotein N/β-actin; (**G**) RSV fusion protein F1/β-actin.

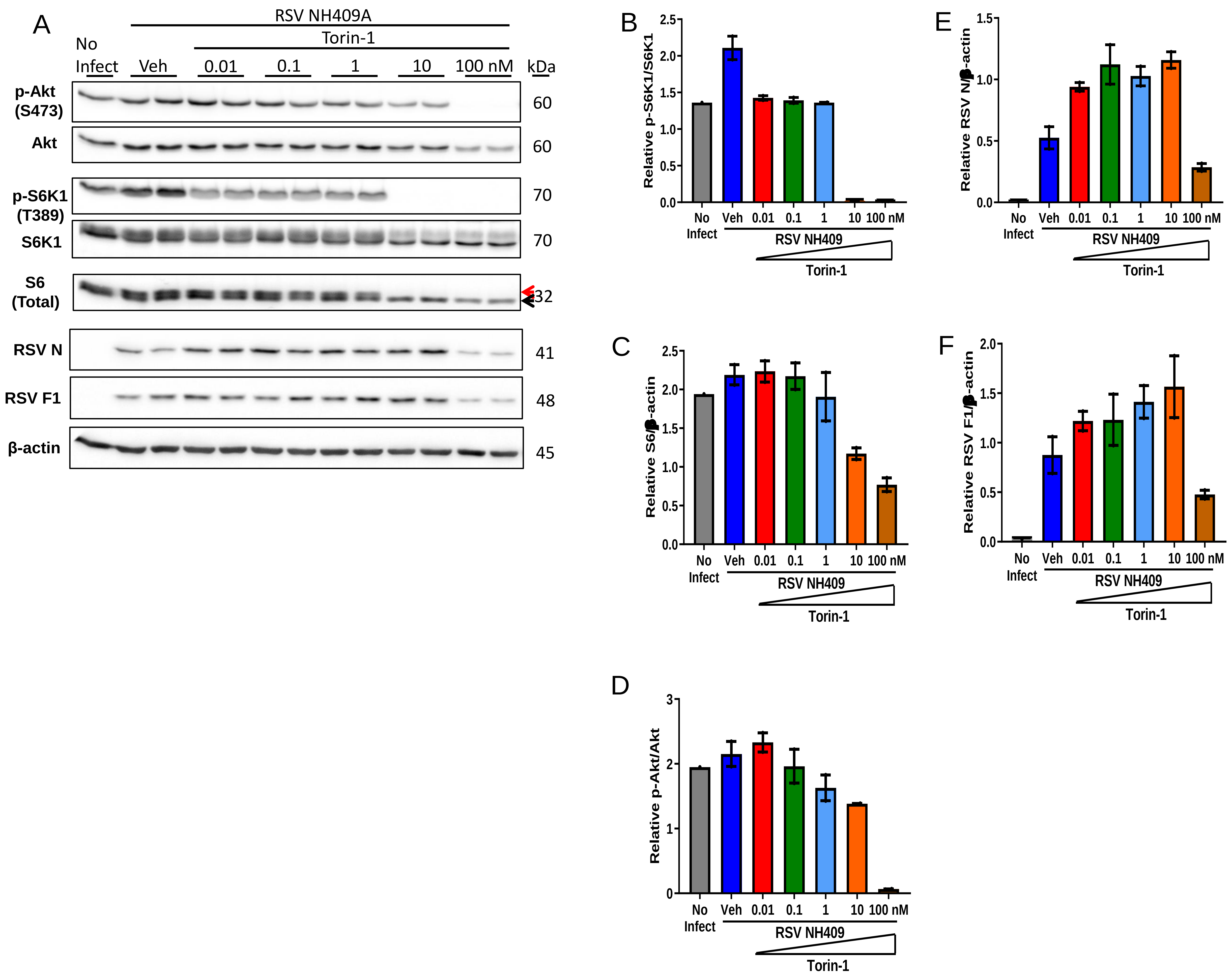

Fig. S6. Torin-1 Reduces viral protein expression through Inhibition of mTORC1 and mTORC2.

A549 cells were infected with RSV NH409A and subsequently treated with varying concentrations of Torin-1. Protein analyses and viral replication was measured at 24 hours post infection (m.o.i = 0.2)

(**A**) Western blot of cellular and viral proteins.

(**B-F**) Quantification of protein displayed in A (n = 2).

(**B**) phospho-S6K1/S6K1; (**C**) total ribosomal protein S6/β-actin ; (**D**) phospho-Akt/Akt; (**E**) RSV nucleoprotein N/β-actin; (**F**) RSV fusion protein F1/β-actin.

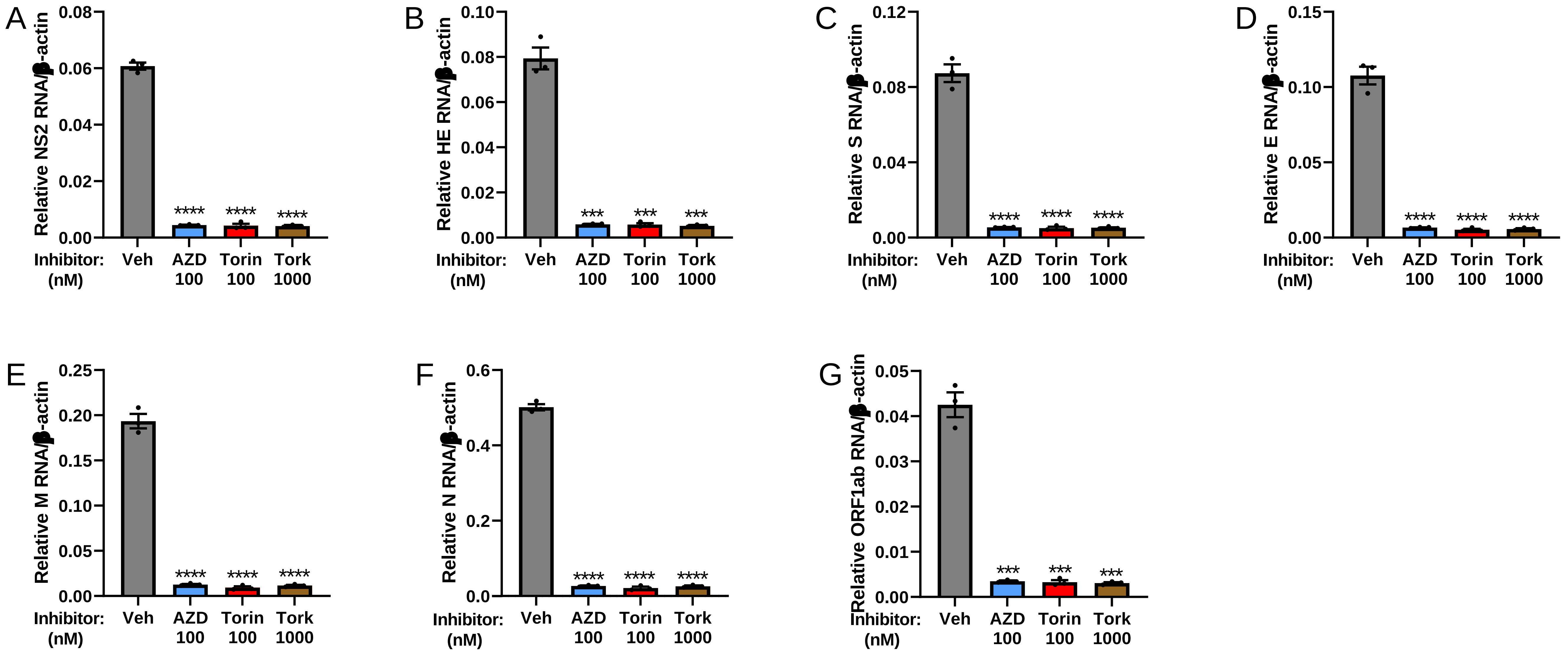

Fig. S7. mTOR inhibitors block HcoV OC43 viral RNA expression.

A549 cells were pre-treated for 2 hours with indicated concentration of AZD-8055, Torin-1, or Torkinib, and subsequently infected with HcoV OC43 for 1.5 hours. mTOR inhibitors were continuously presence in media. Viral RNA analyses was measured at 24 hours post infection (m.o.i = 0.3).

(**A-G**) Quantitative (qRT-PCR) PCR of viral RNA expression at presence of indicated concentration of mTOR inhibitors (n = 3).

(**A**) non-structural protein 2 NS2; (**B**) hemagglutinin-esterase HE; (**C**) spike surface glycoprotein S; (**D**) envelope protein E; (**E**) membrane protein M; (**F**) nucleocapsid protein N; (**G**) replicase polyprotein ORF1ab.

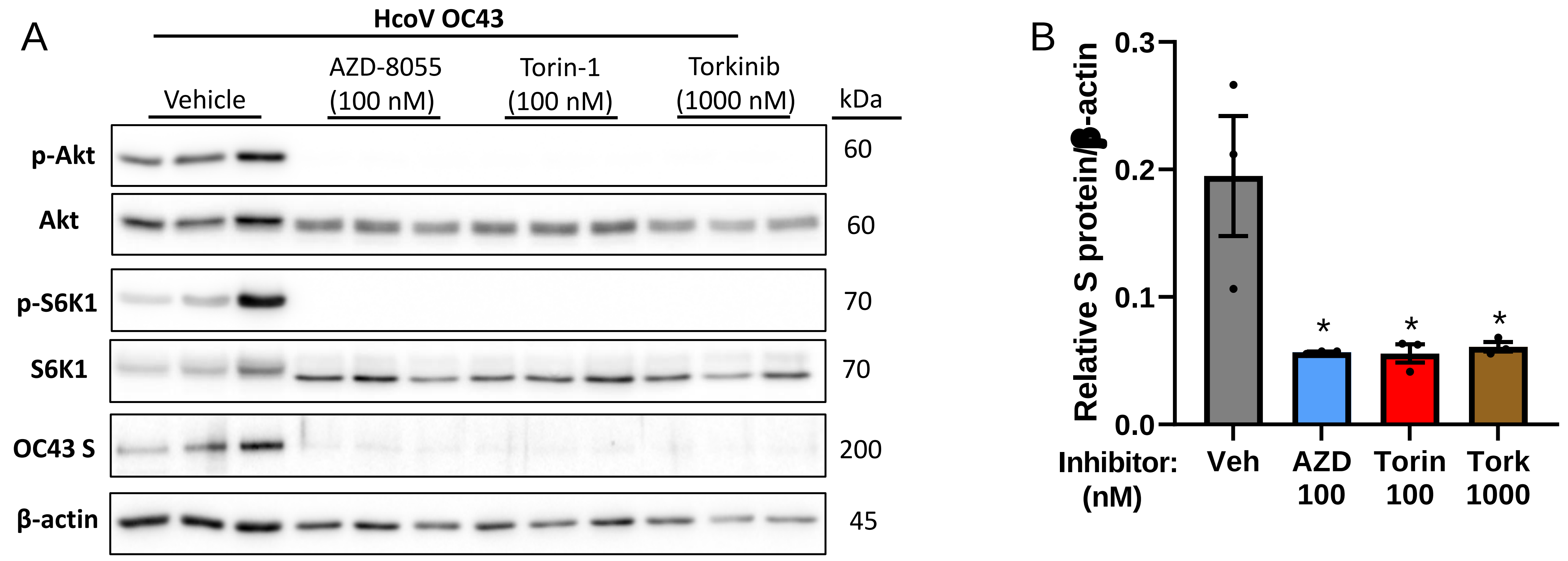

Fig. S8. mTOR inhibitors block HcoV OC43 viral protein spike S synthesis.

A549 cells were infected with HcoV OC43 for 1.5 hours and subsequently treated with indicated concentration of mTOR inhibitors. Protein analyses was measured at 24 hours post infection (m.o.i = 1).

(**A**) western blot of cellular and viral proteins at presence of indicated concentration of mTOR inhibitors; (**B**) quantification of viral protein spike S displayed in panel A (n = 3).

Raw Images of Western Blot for Figure 1A

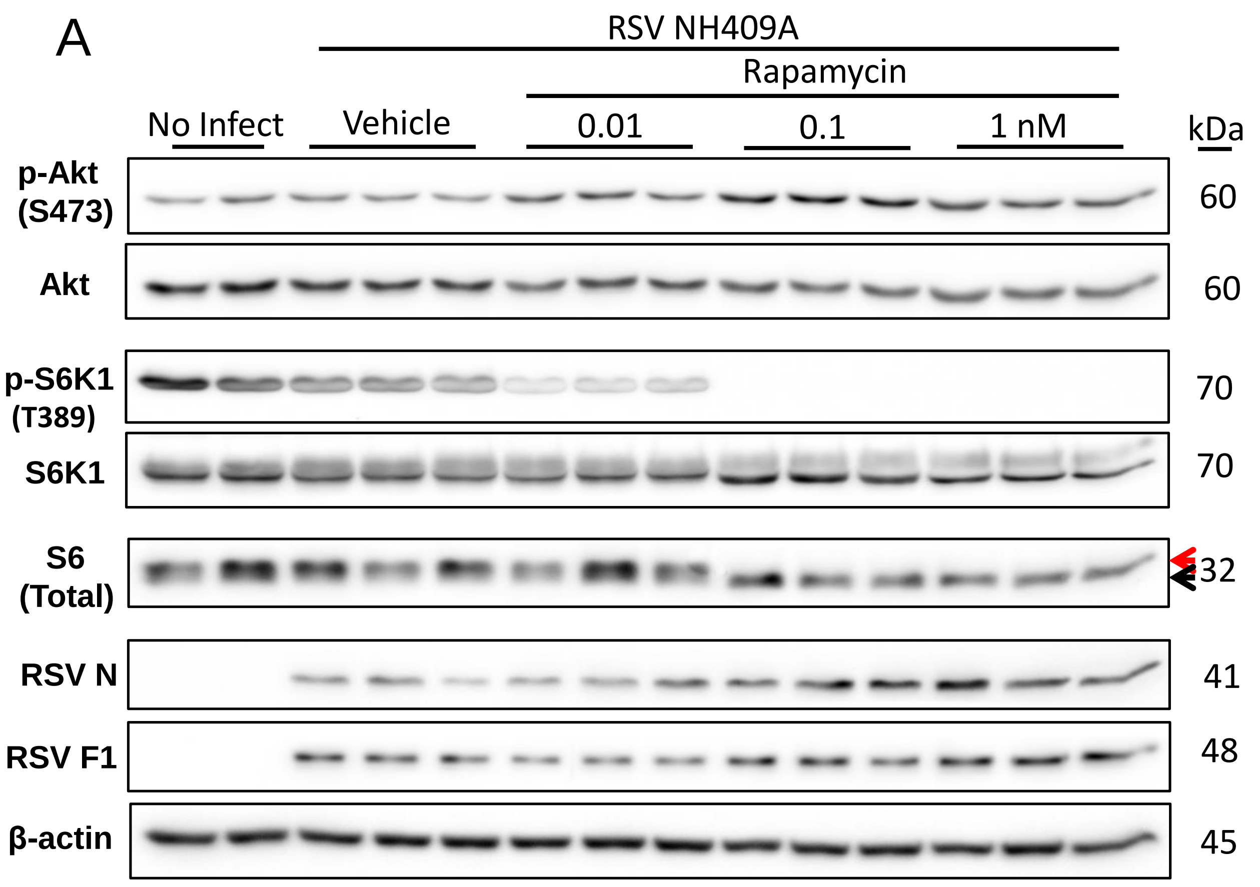

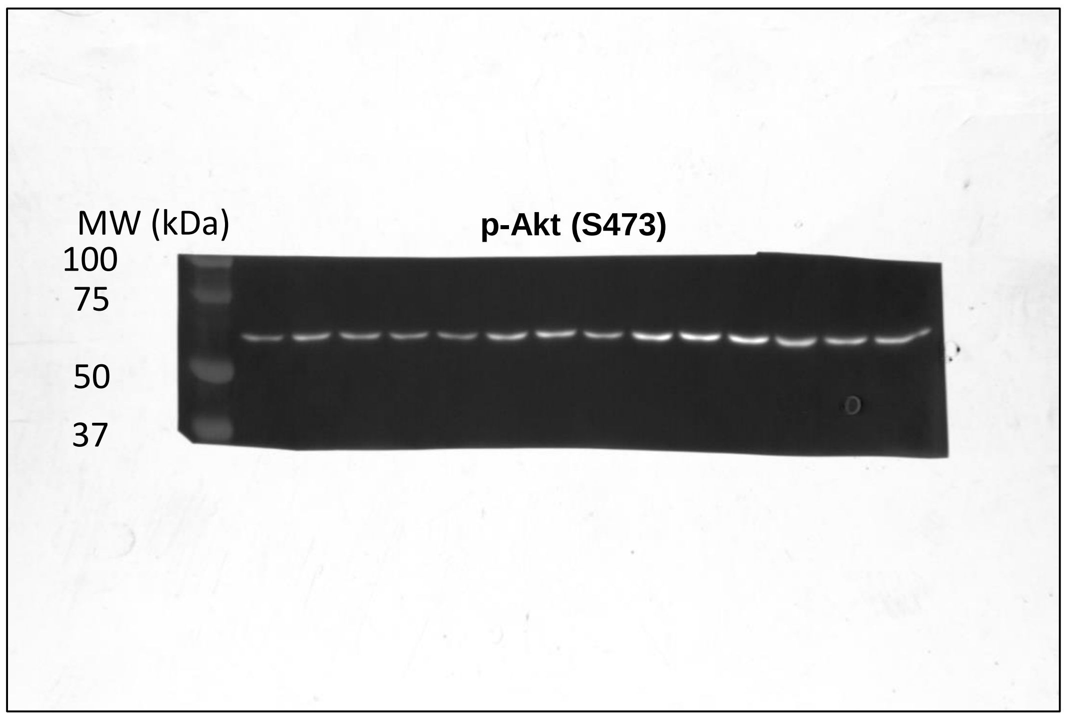

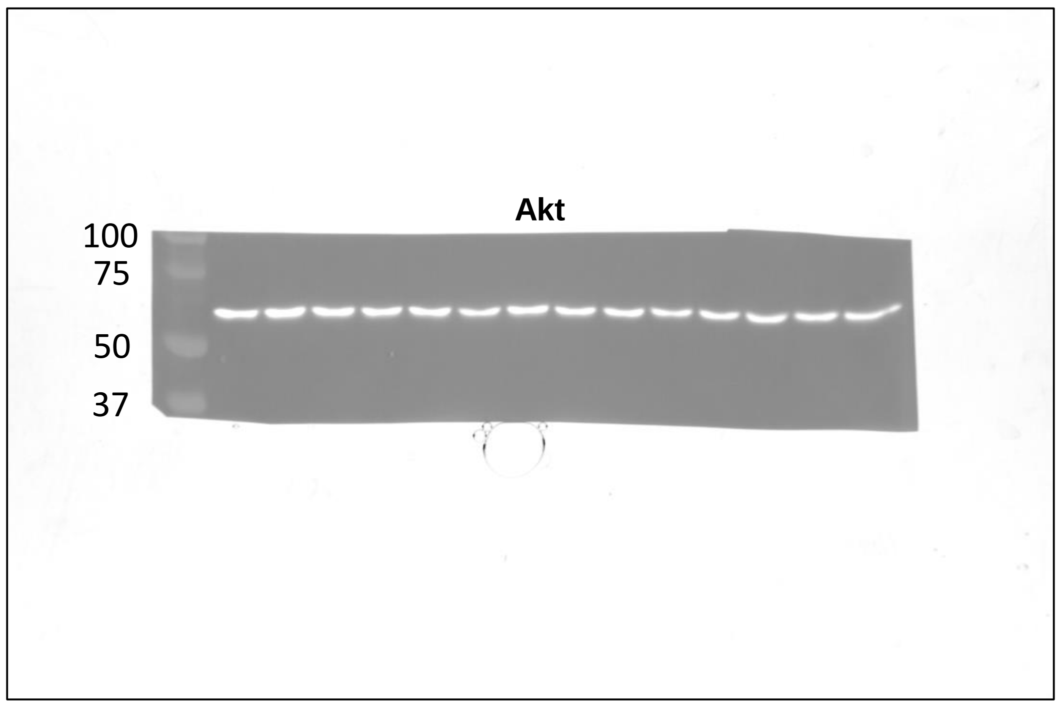

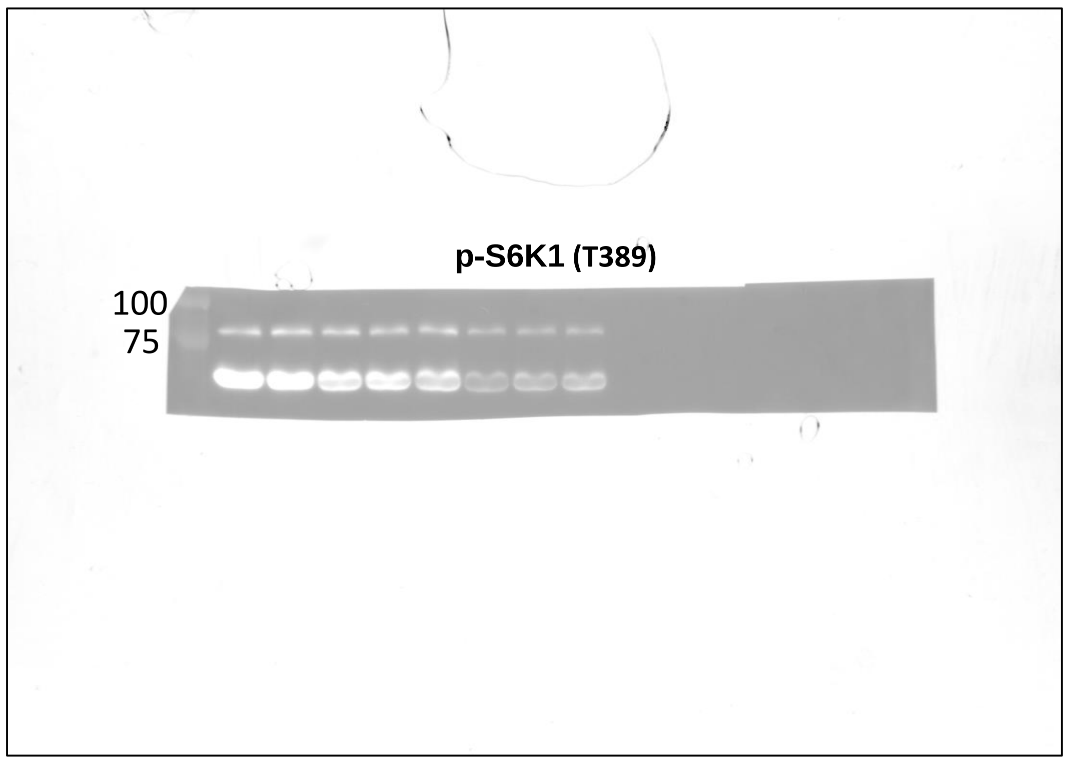

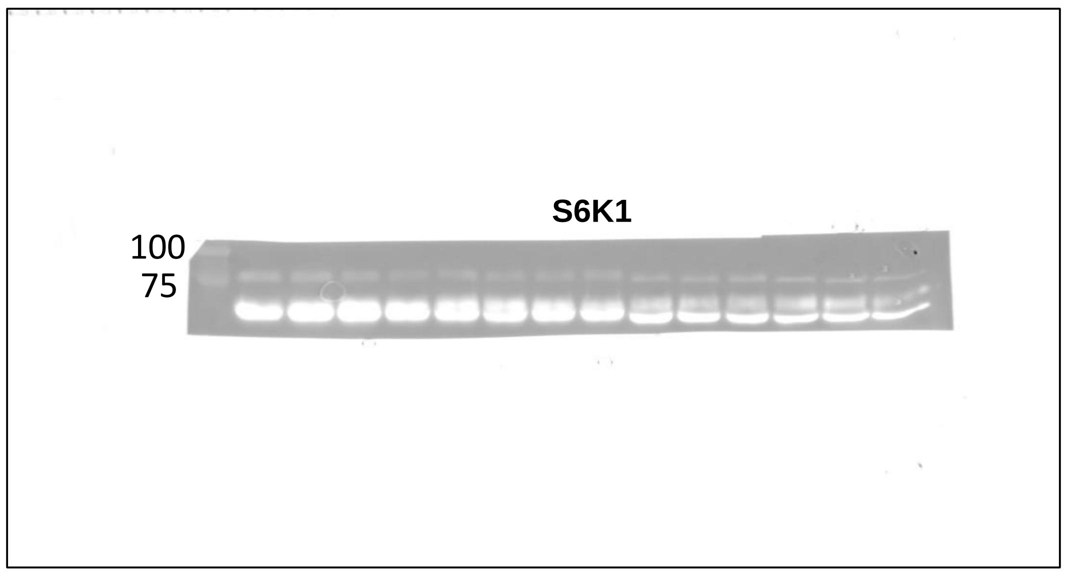

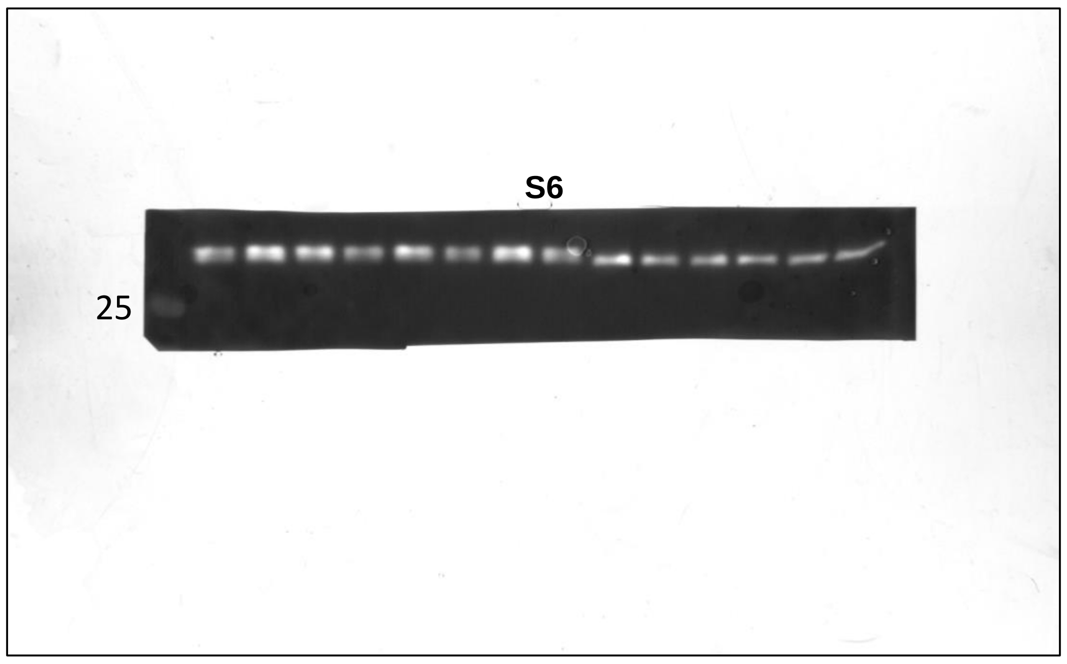

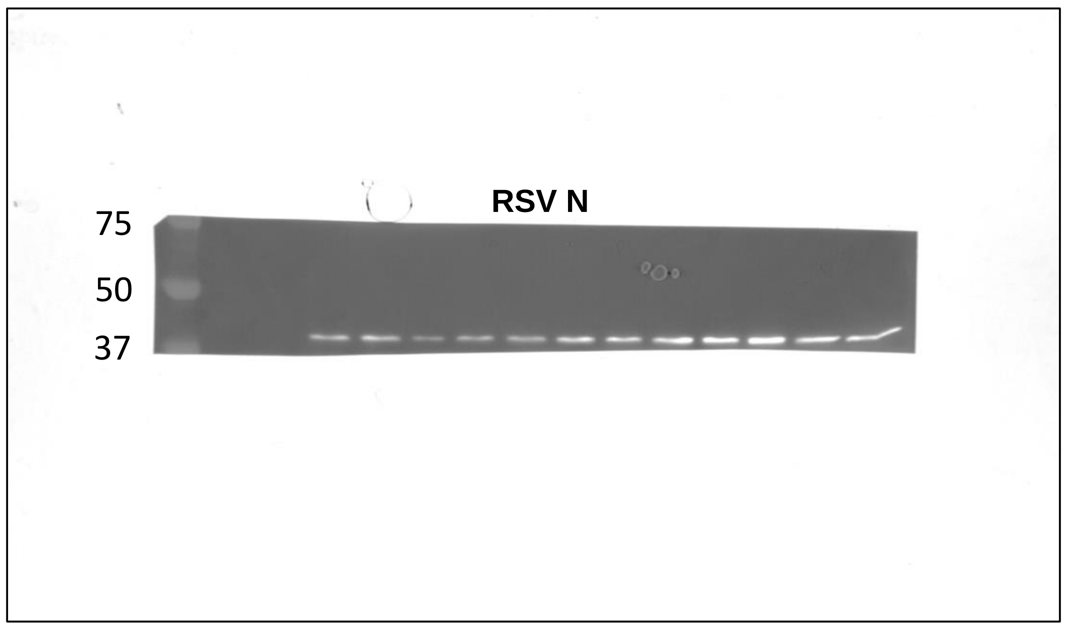

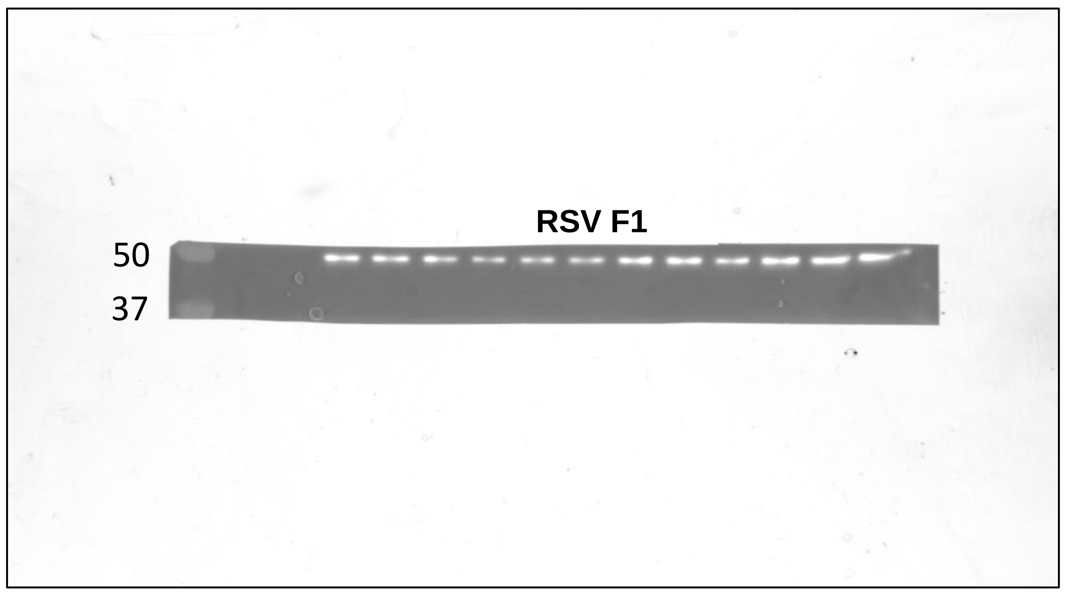

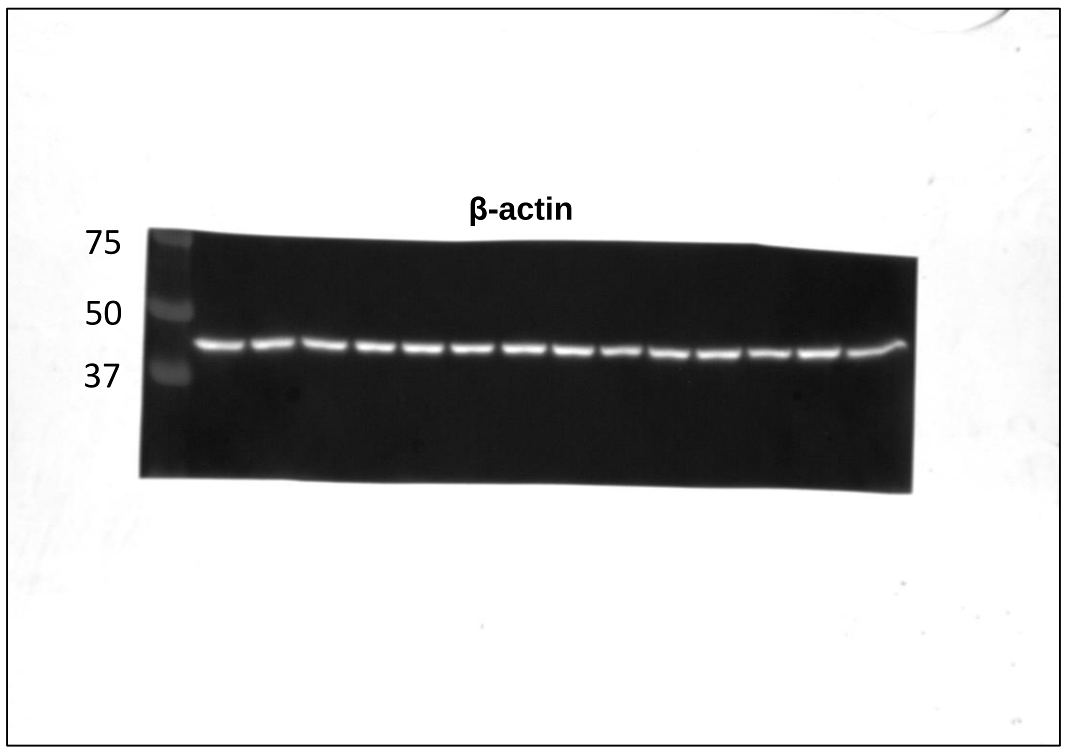

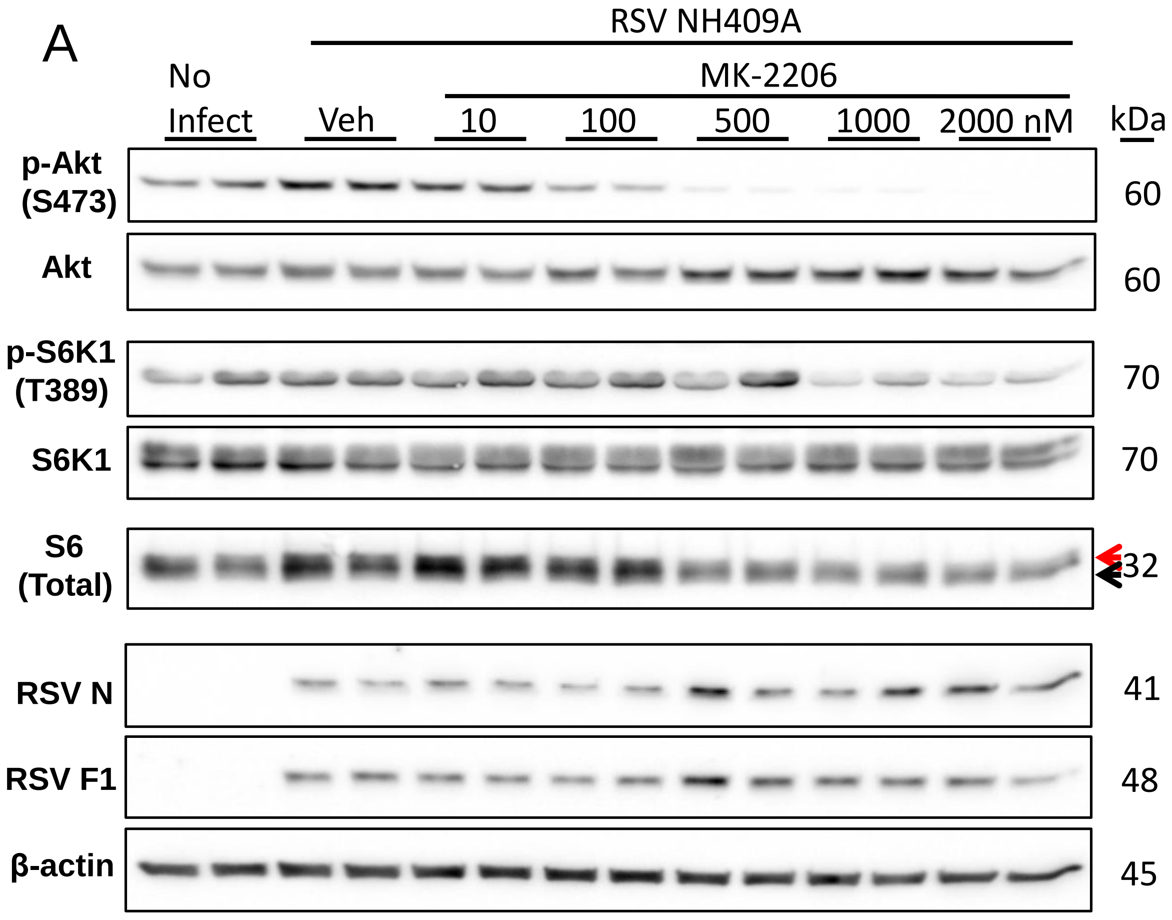
A
RSV NH409A
MK-2206
No Infect
Veh
10
100
500
1000
2000 nM
kDa
p-Akt (S473)
60
Akt
60
p-S6K1 (T389)
70
S6K1
70
S6 (Total)
32
RSV N
41
RSV F1
48
β-actin
45

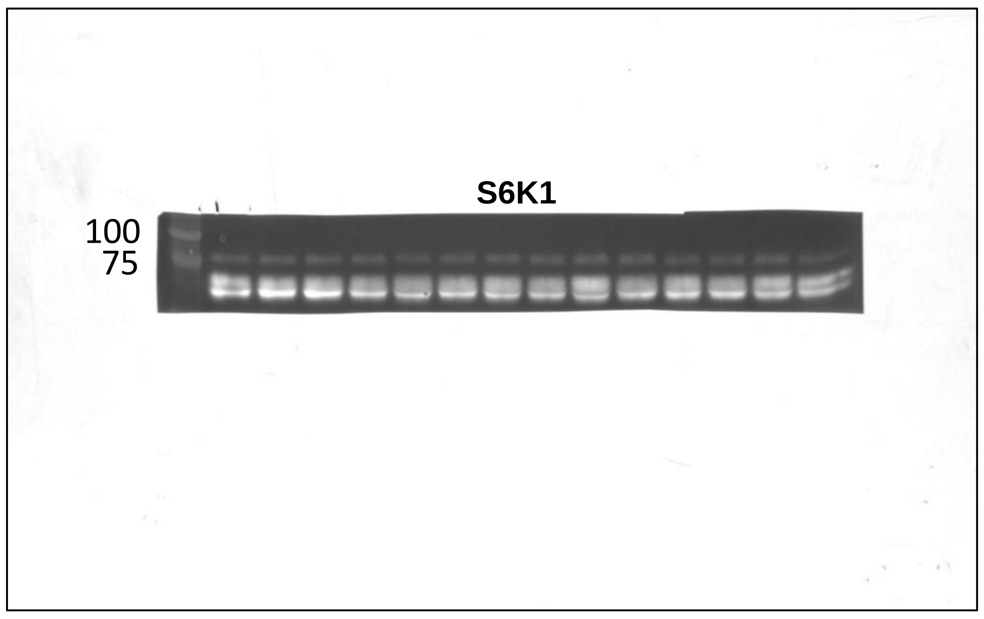
S6K1
100
75

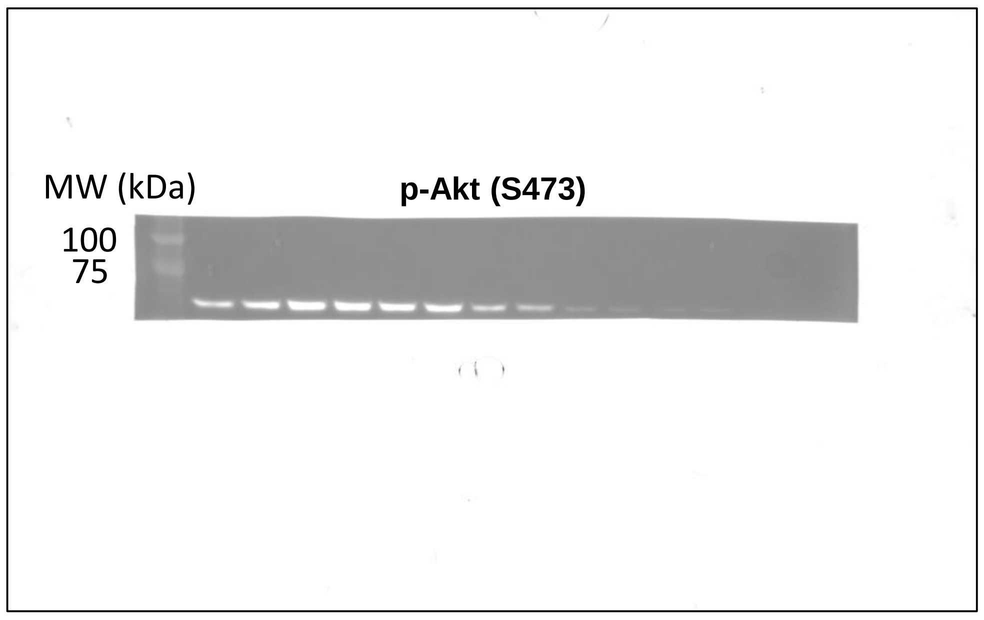
MW (kDa)
p-Akt (S473)
100
75

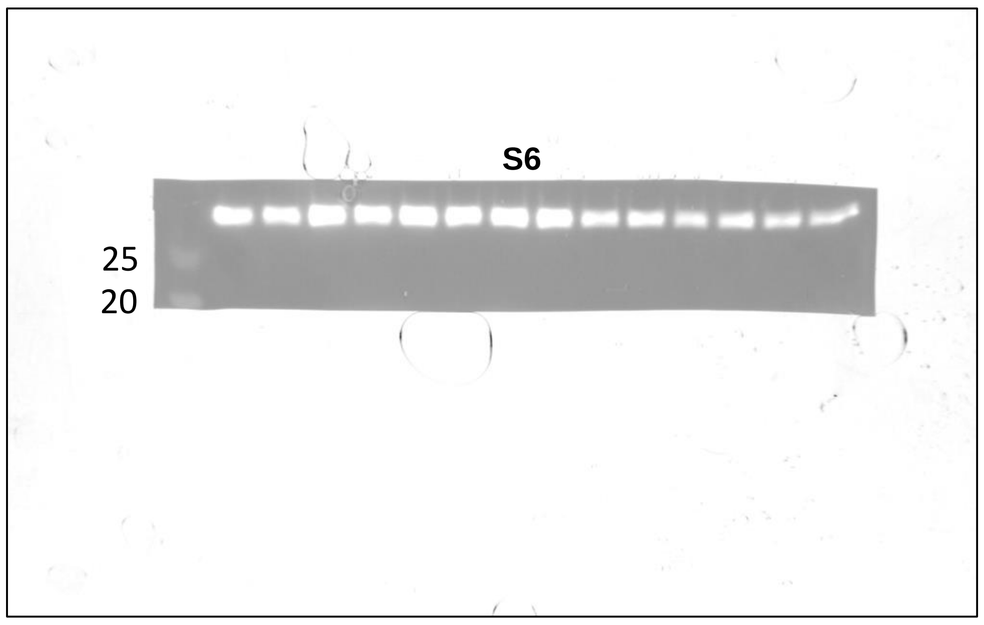
S6
25
20

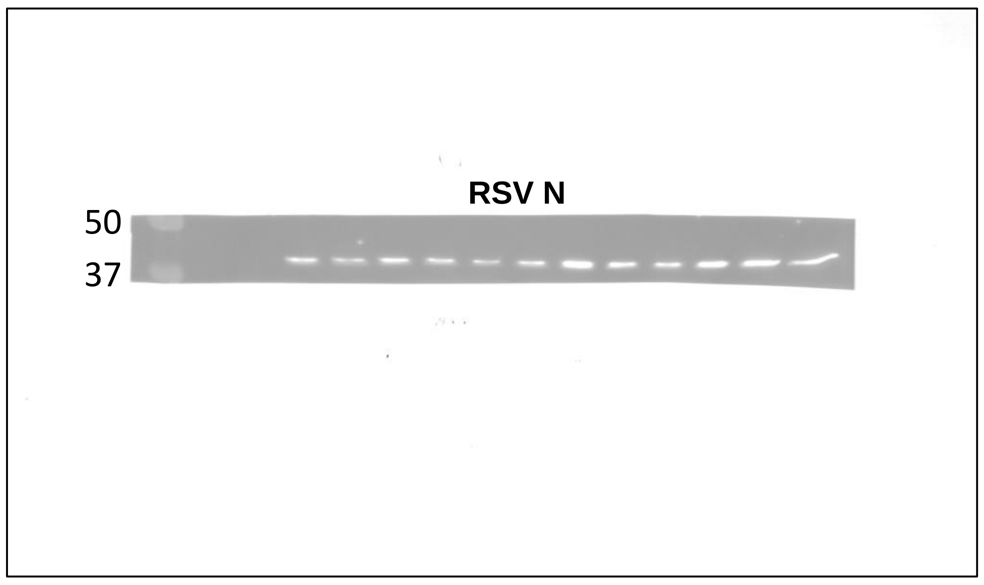
RSV N
50
37

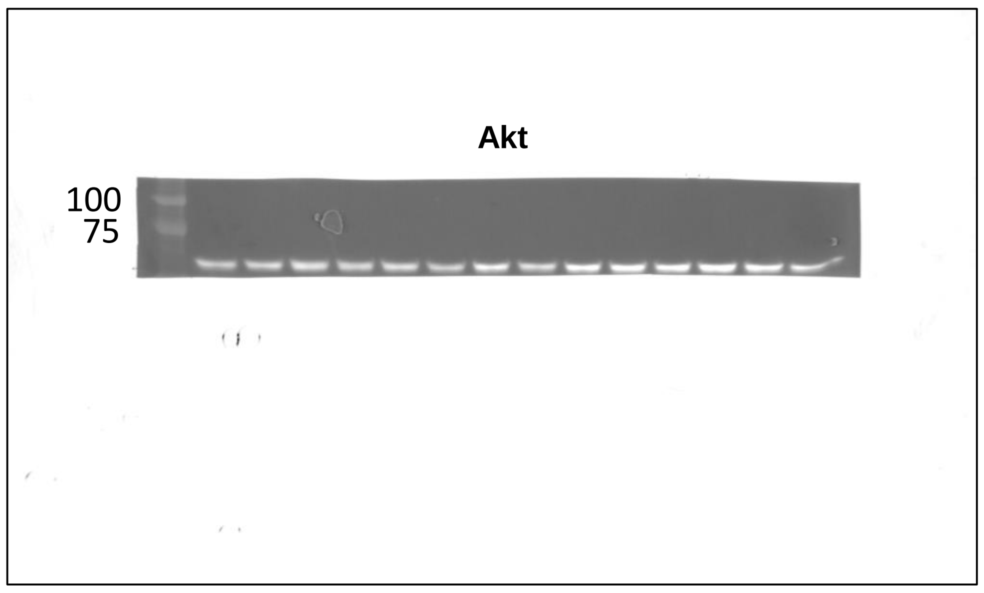
Akt
100
75

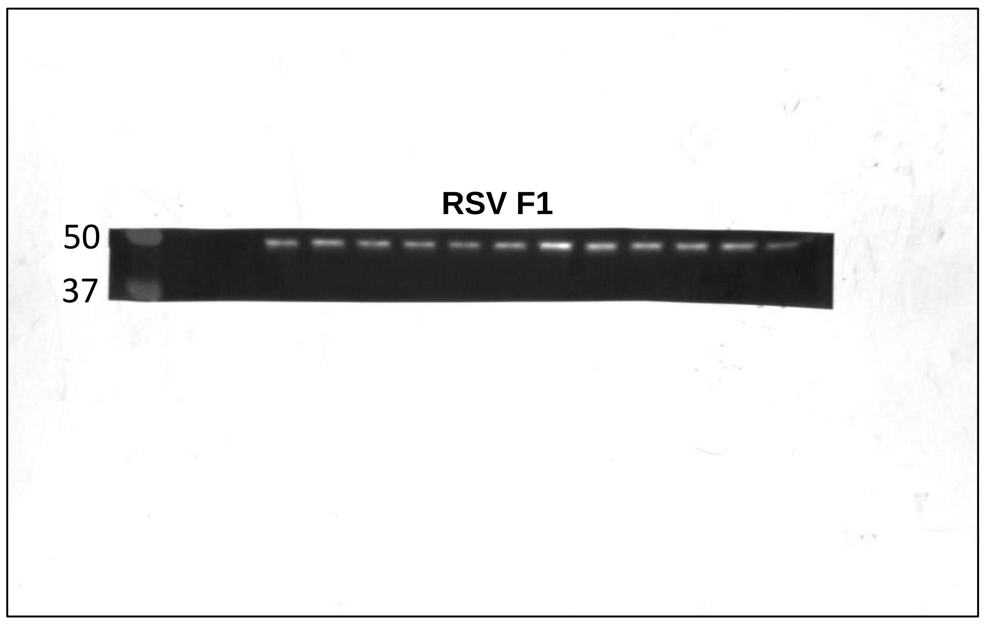
RSV F1
50
37

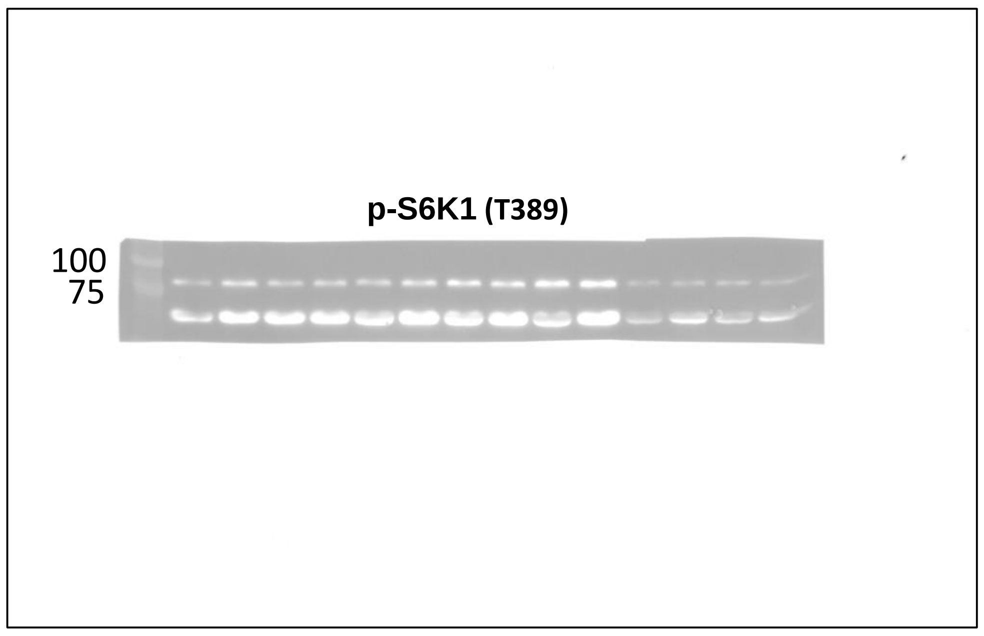
p-S6K1 (T389)
100
75

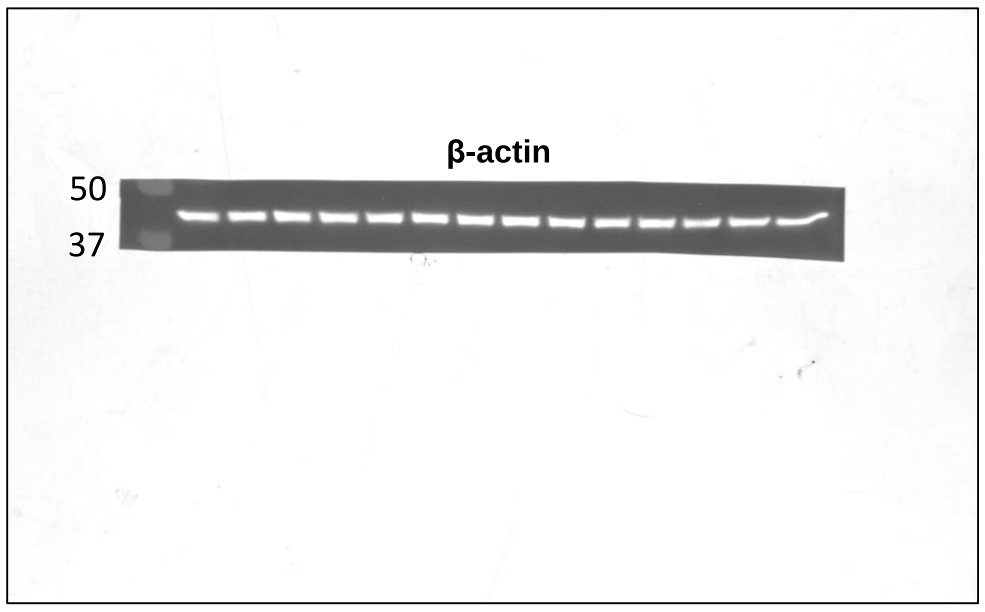
β-actin
50
37

Raw Images of Western Blot for Figure 3A

Huynh et al

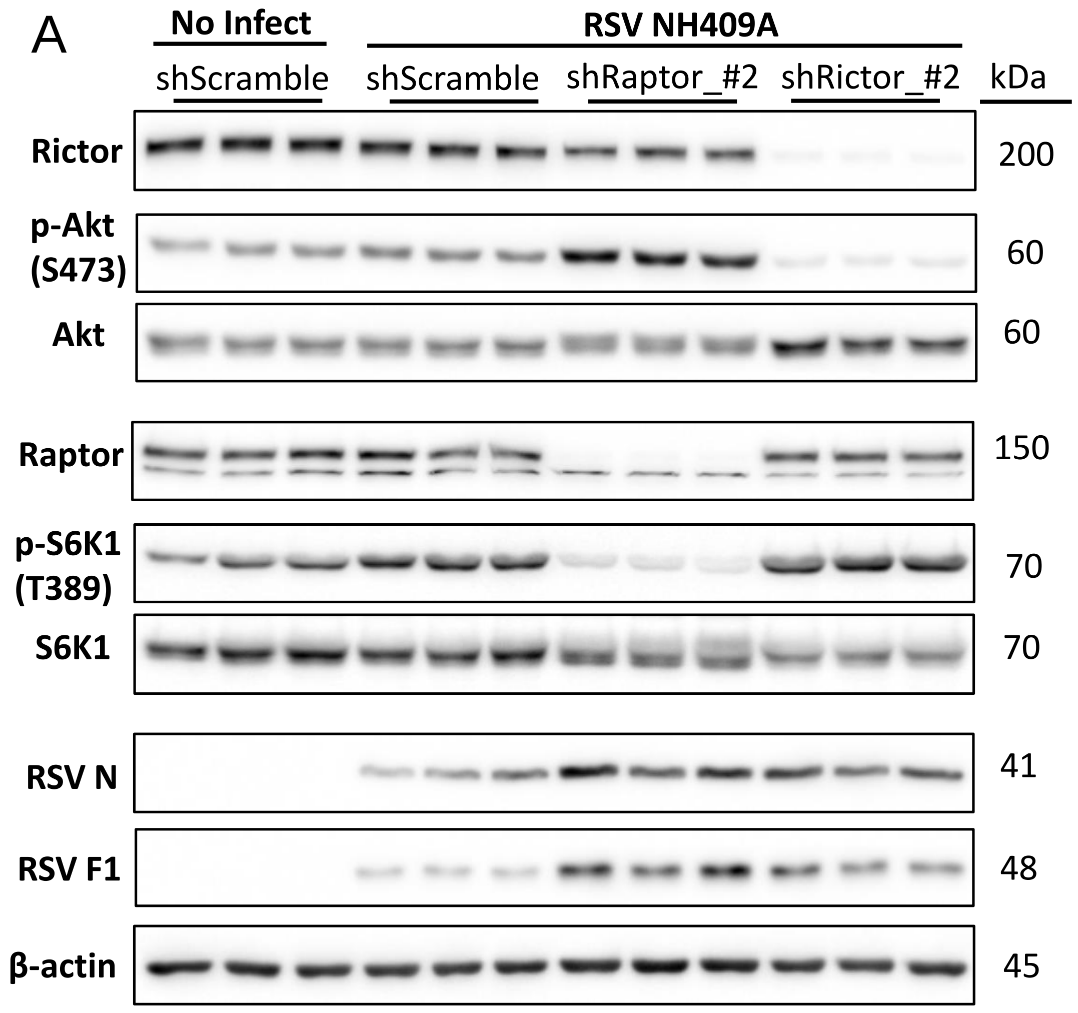

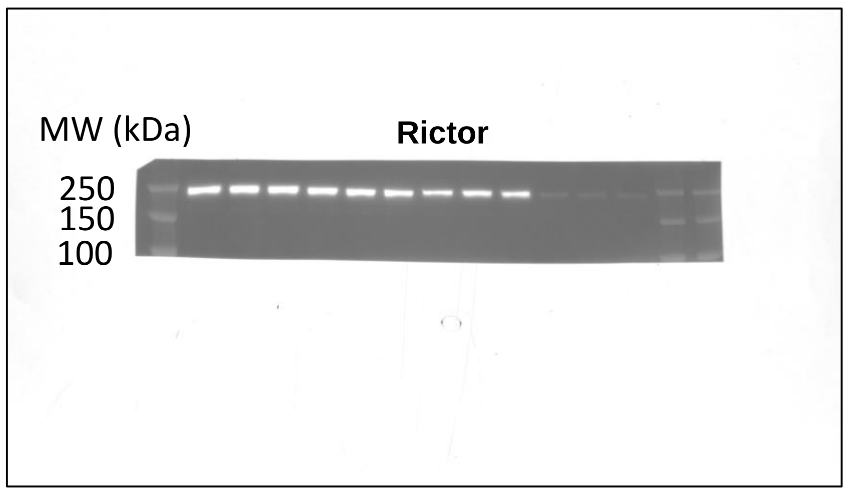

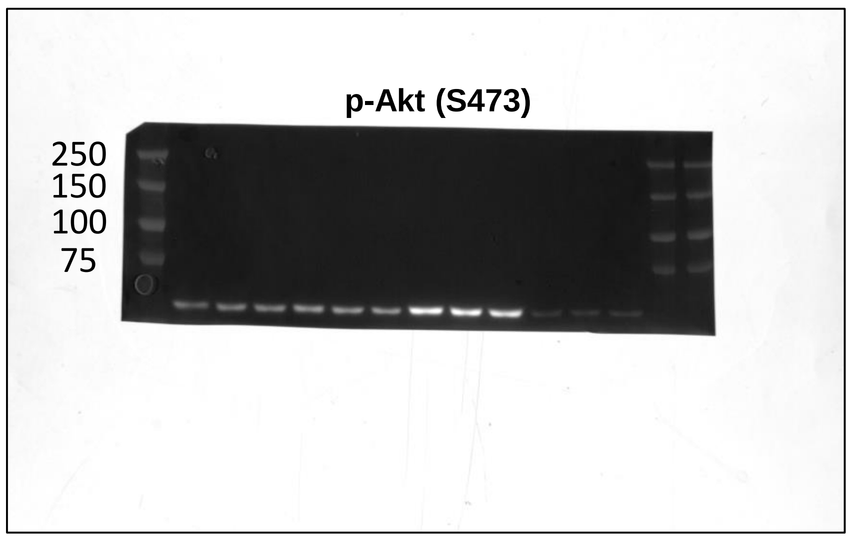

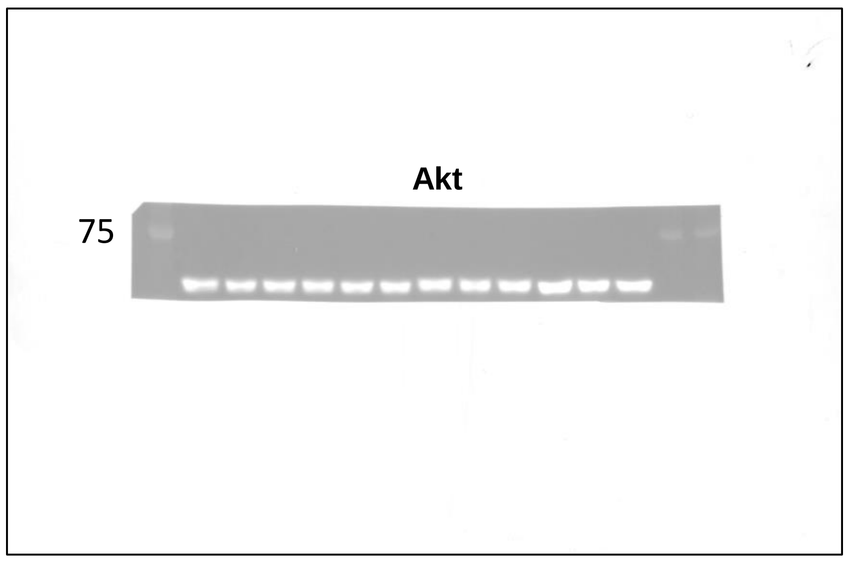

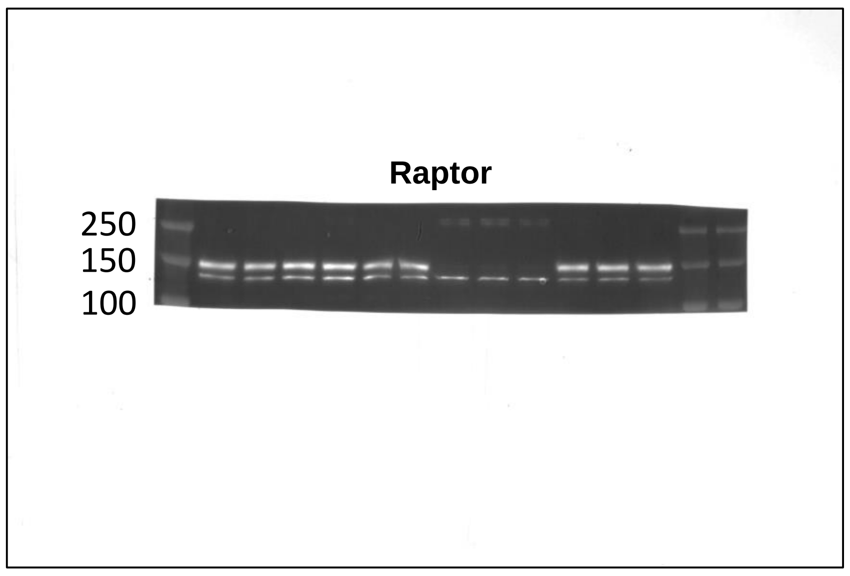

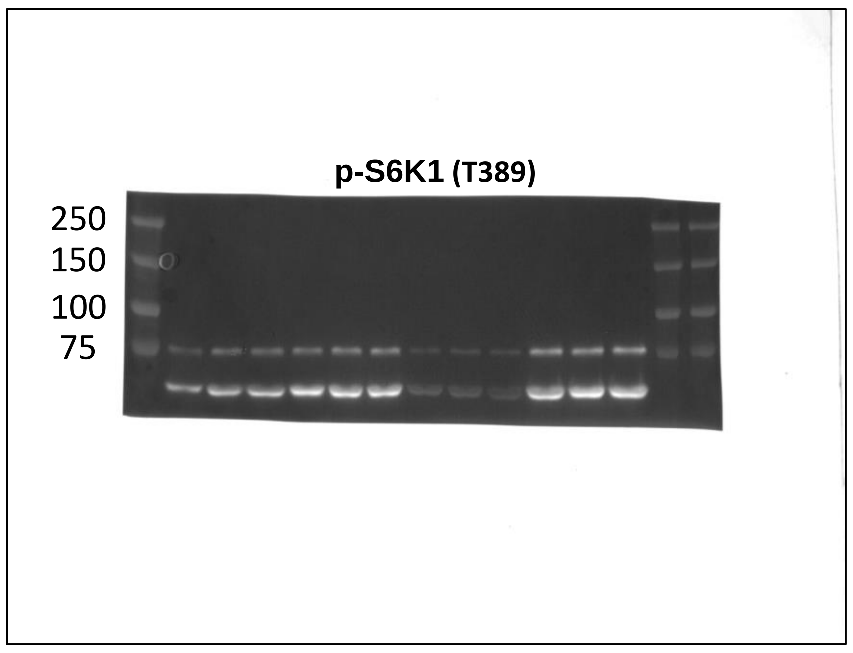

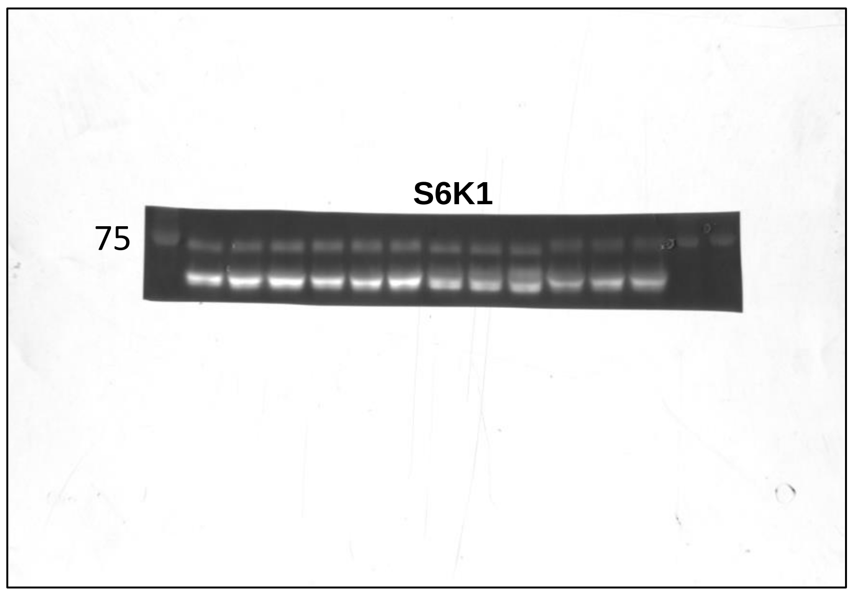

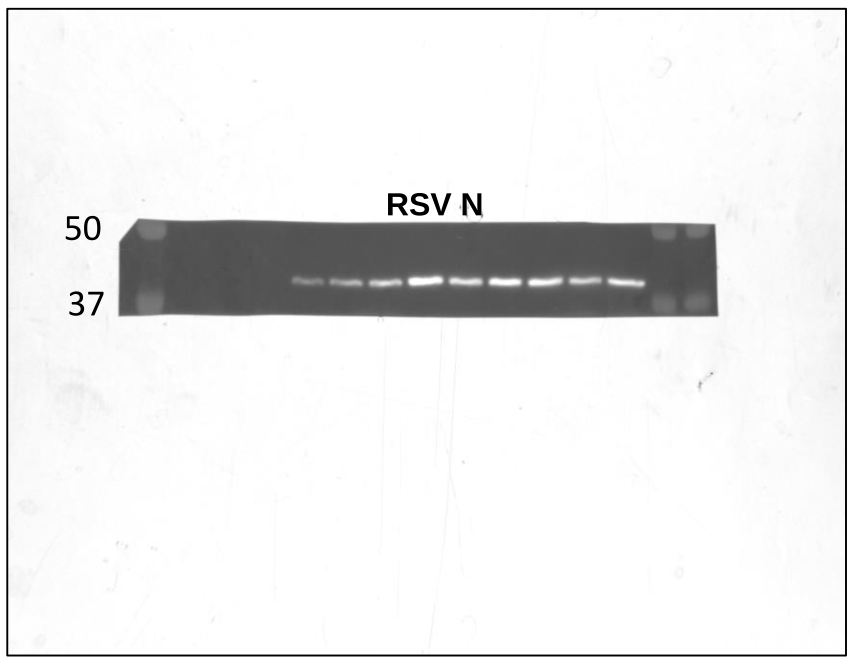

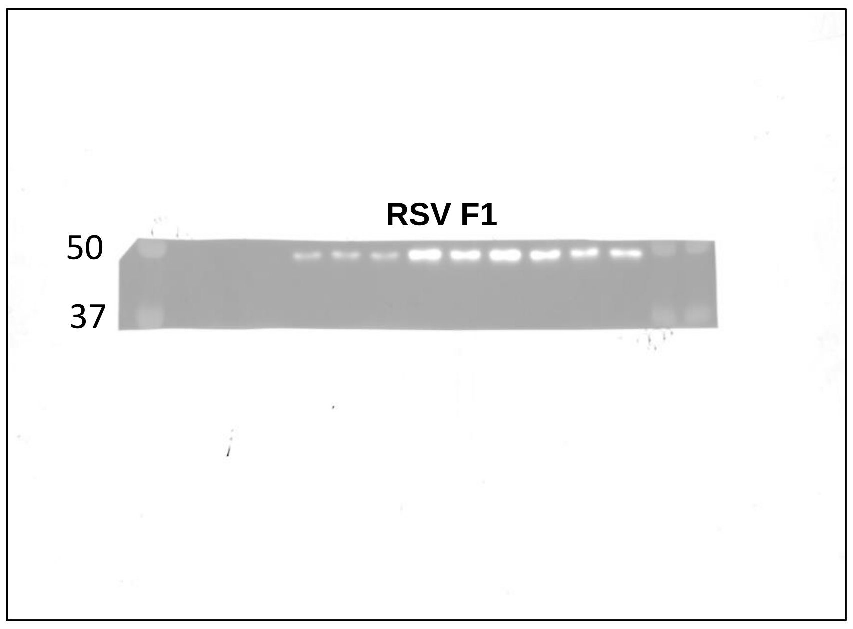

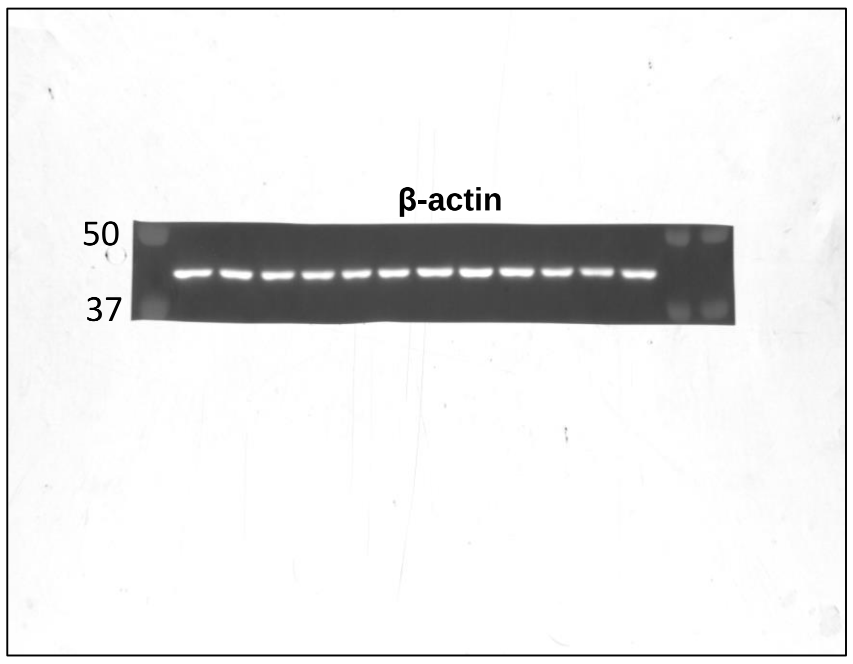

Raw Images of Western Blot for Figure 4A

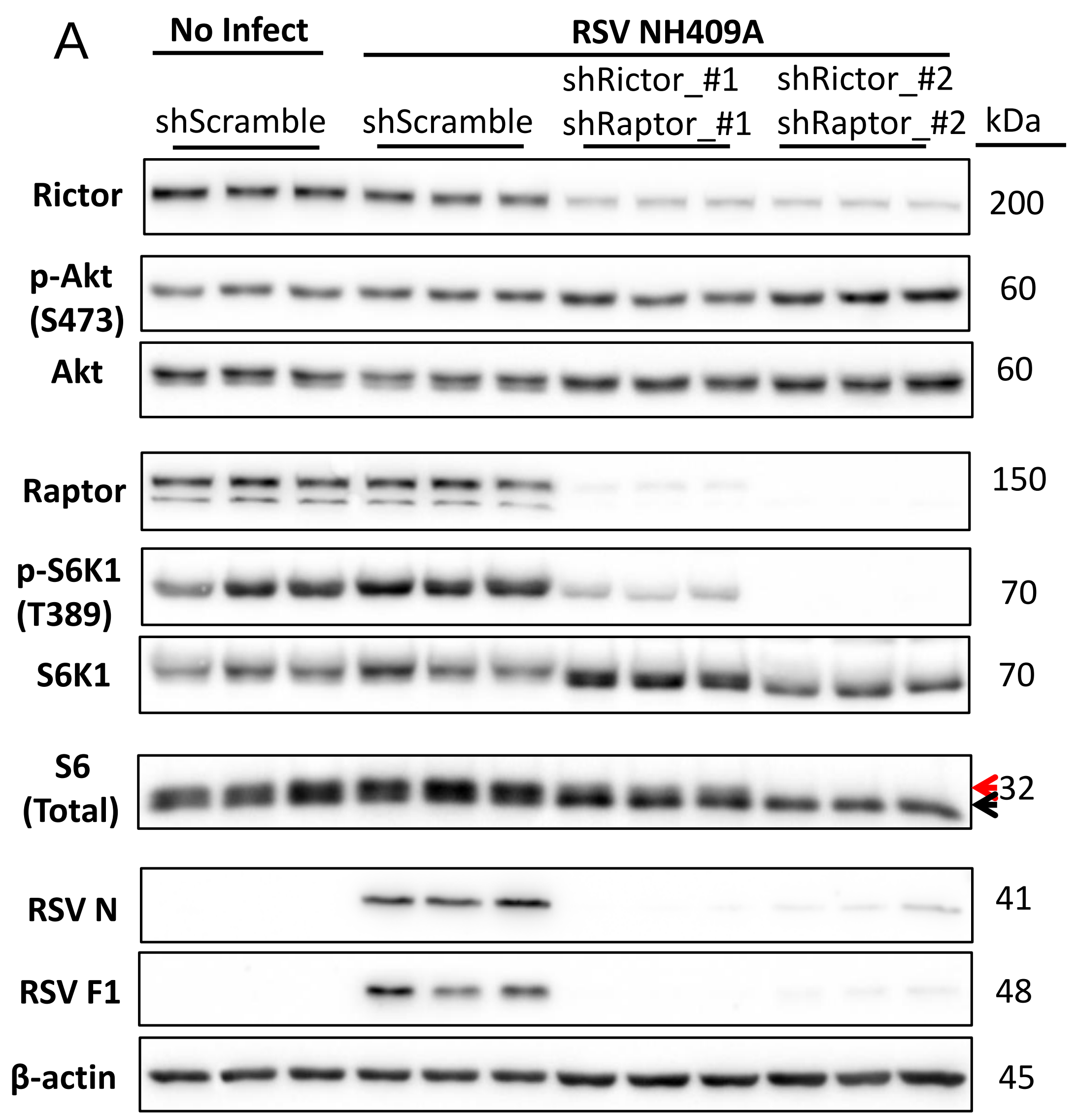

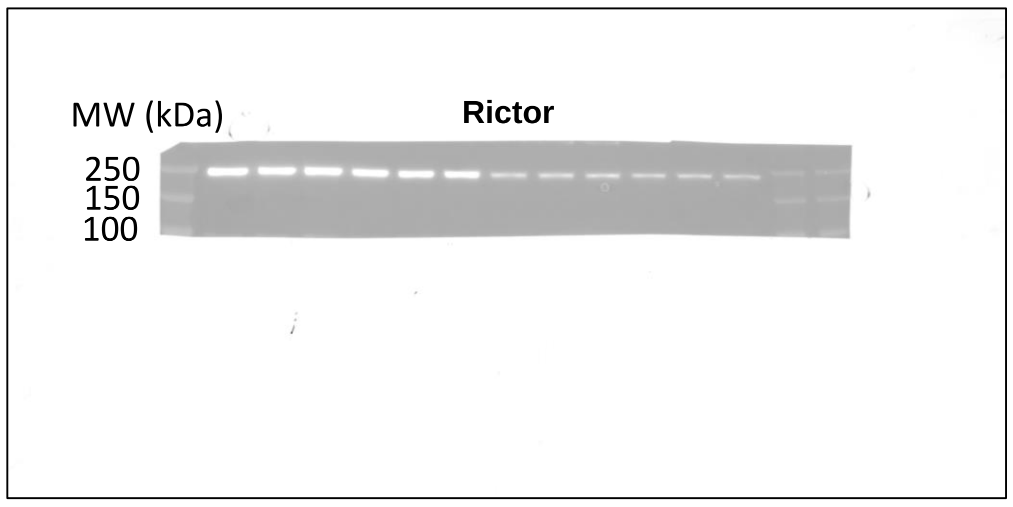

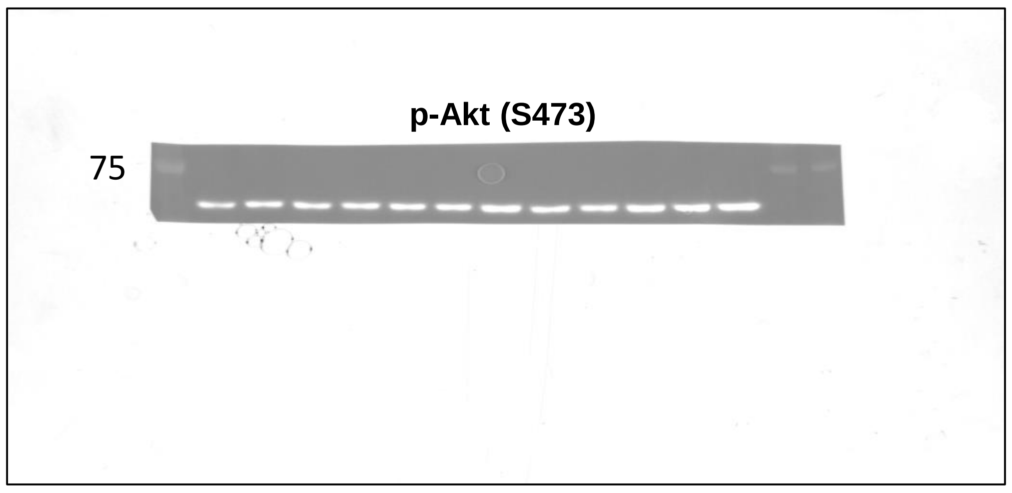

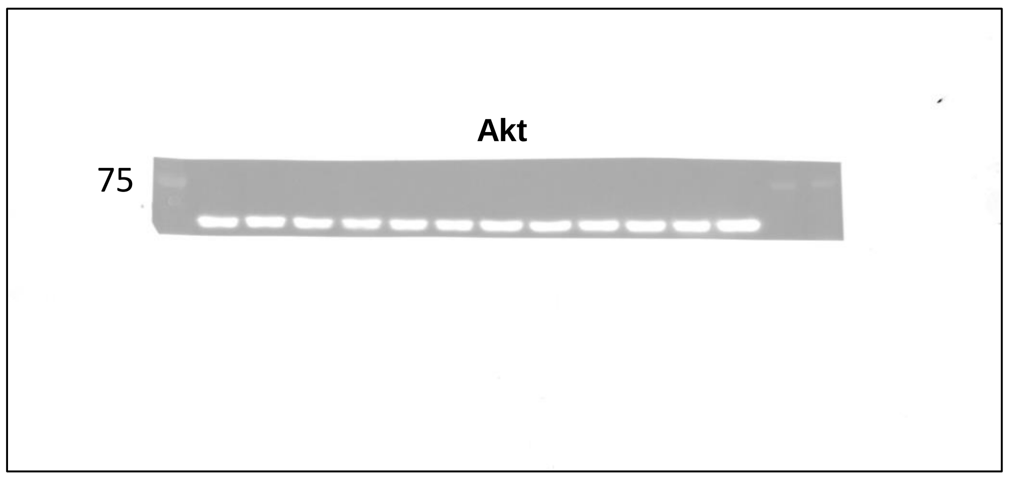

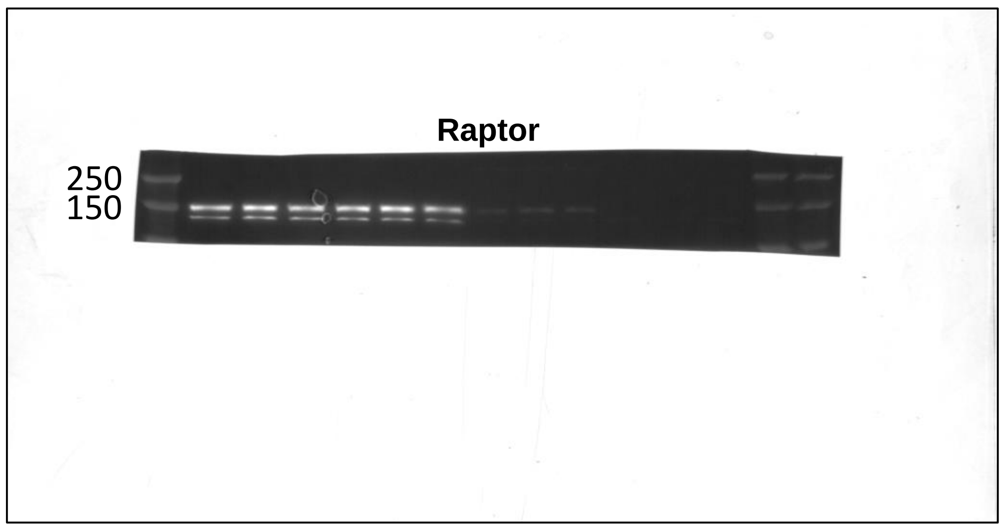

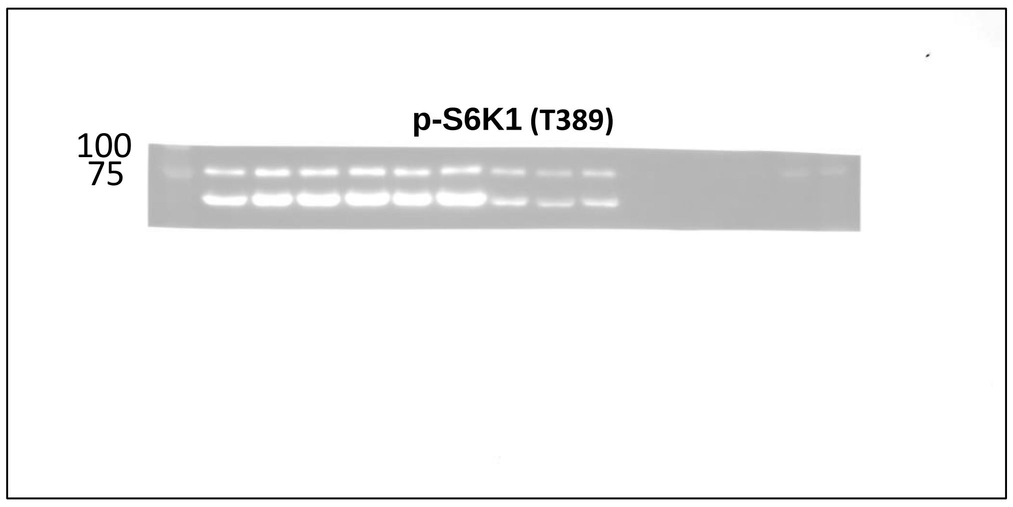

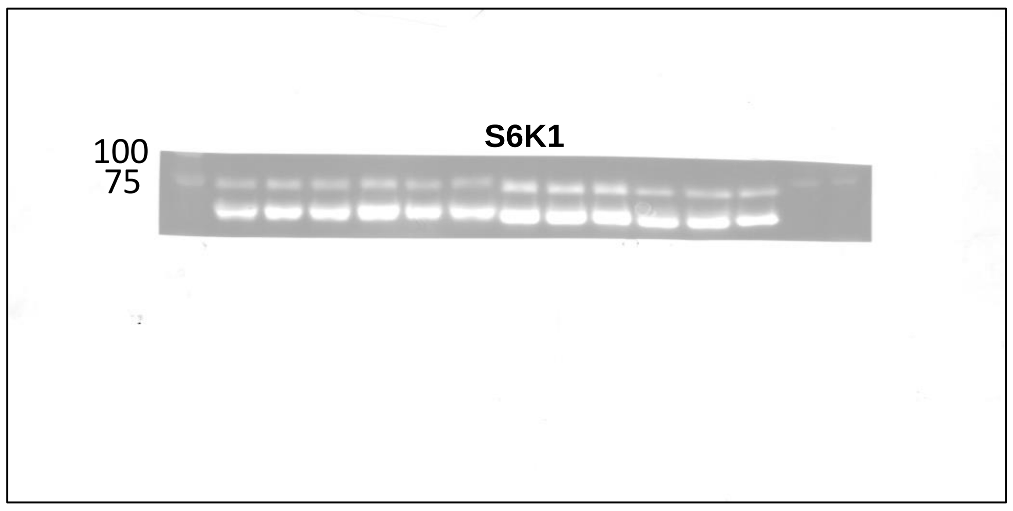

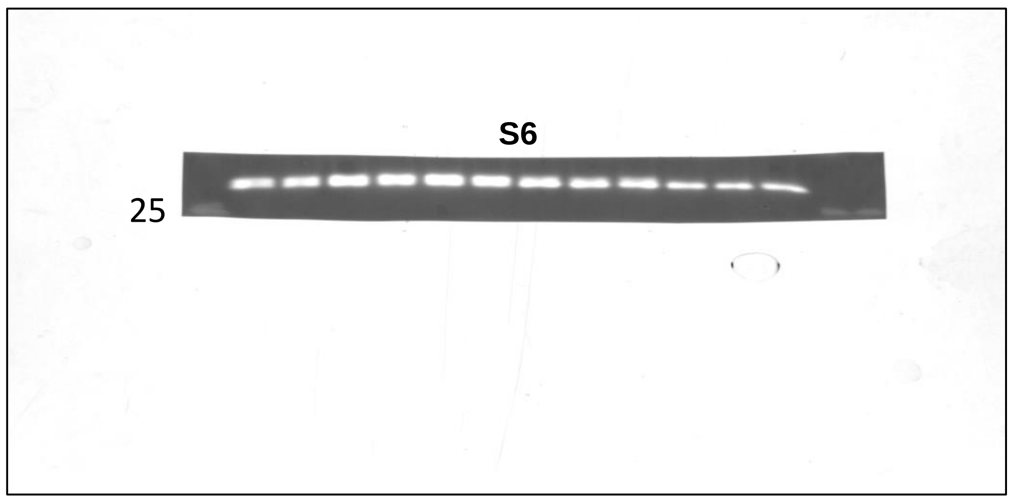

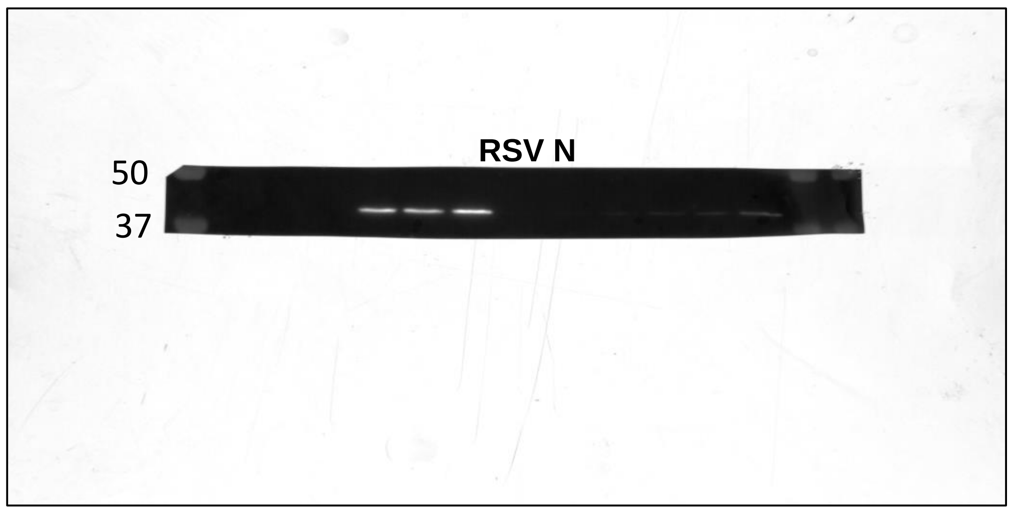

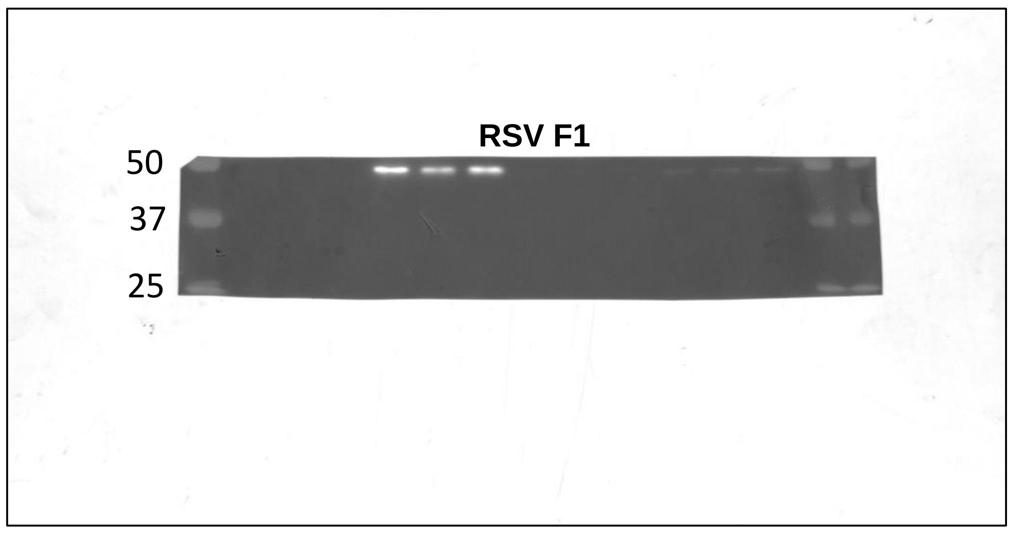

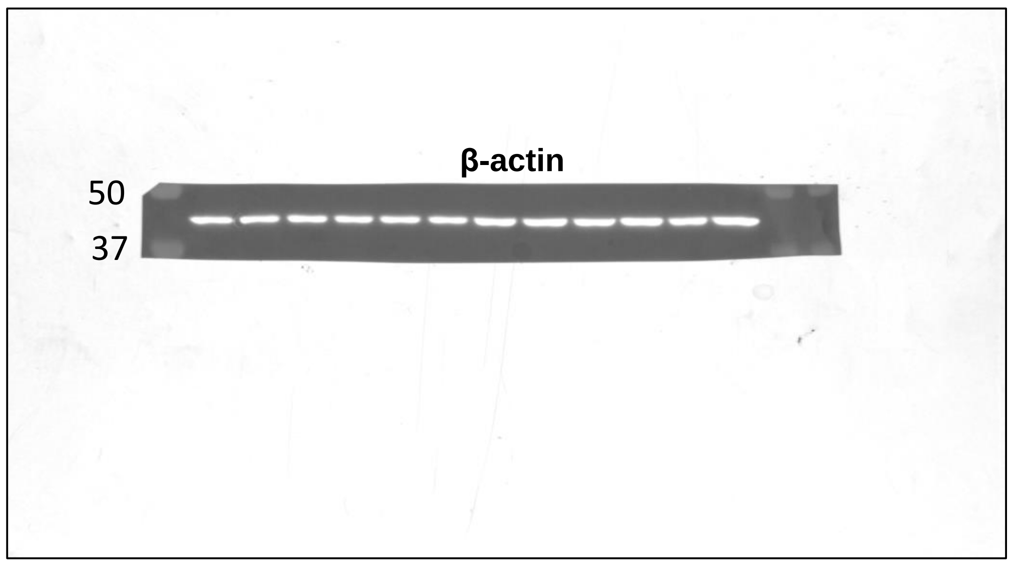

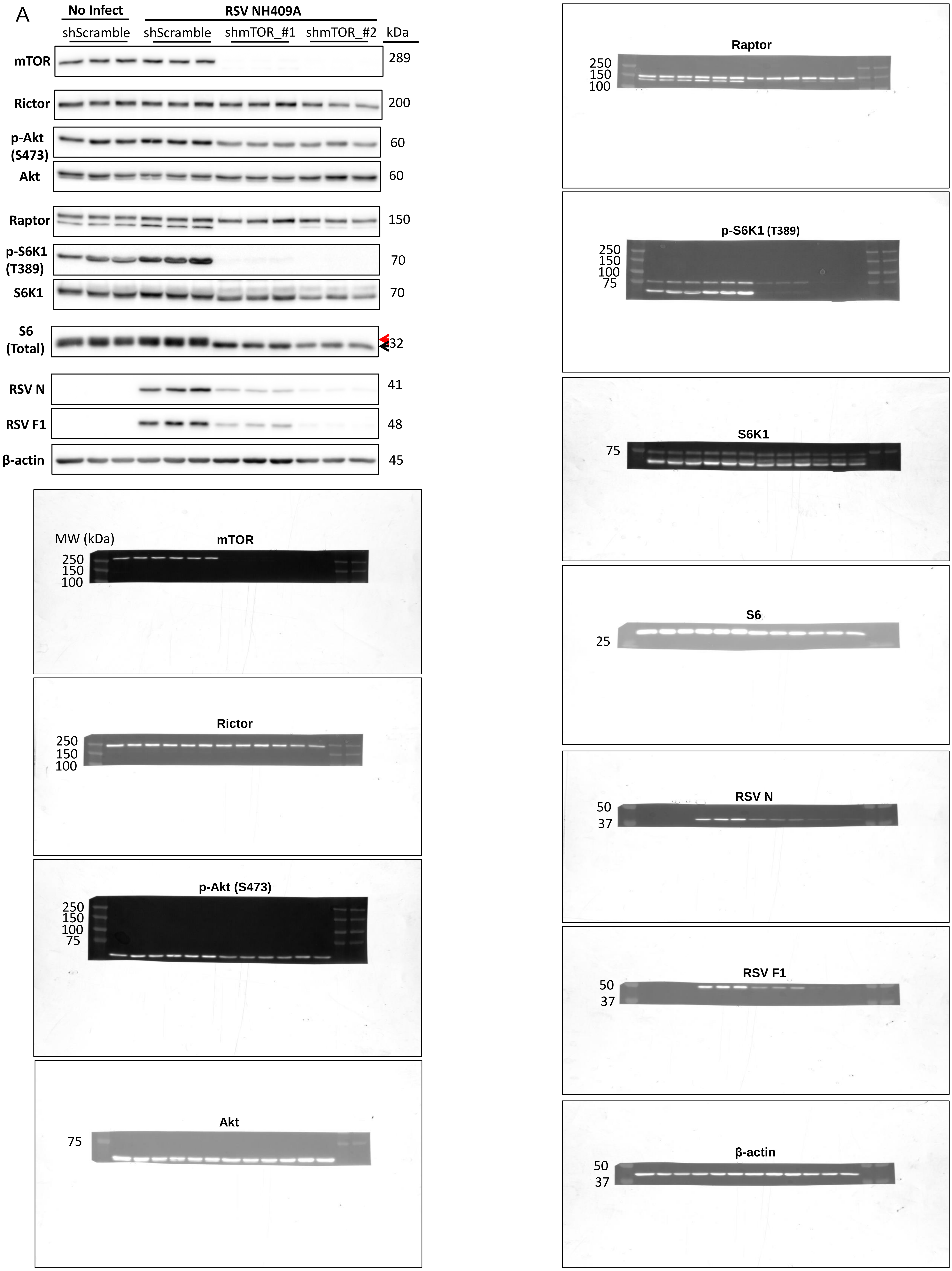
A
No Infect
RSV NH409A
shScramble
shScramble
shmTOR_#1
shmTOR_#2
kDa
mTOR
289
Rictor
200
p-Akt (S473)
60
Akt
60
Raptor
150
p-S6K1 (T389)
70
S6K1
70
S6 (Total)
32
RSV N
41
RSV F1
48
β-actin
45
MW (kDa)
mTOR
250
150
100
Rictor
250
150
100
p-Akt (S473)
250
150
100
75
Akt
75
Raptor
250
150
100
p-S6K1 (T389)
250
150
100
75
S6K1
75
S6
25
RSV N
50
37
RSV F1
50
37
β-actin
50
37

Raw Images of Western Blot for Figure 6A **Huynh et al**

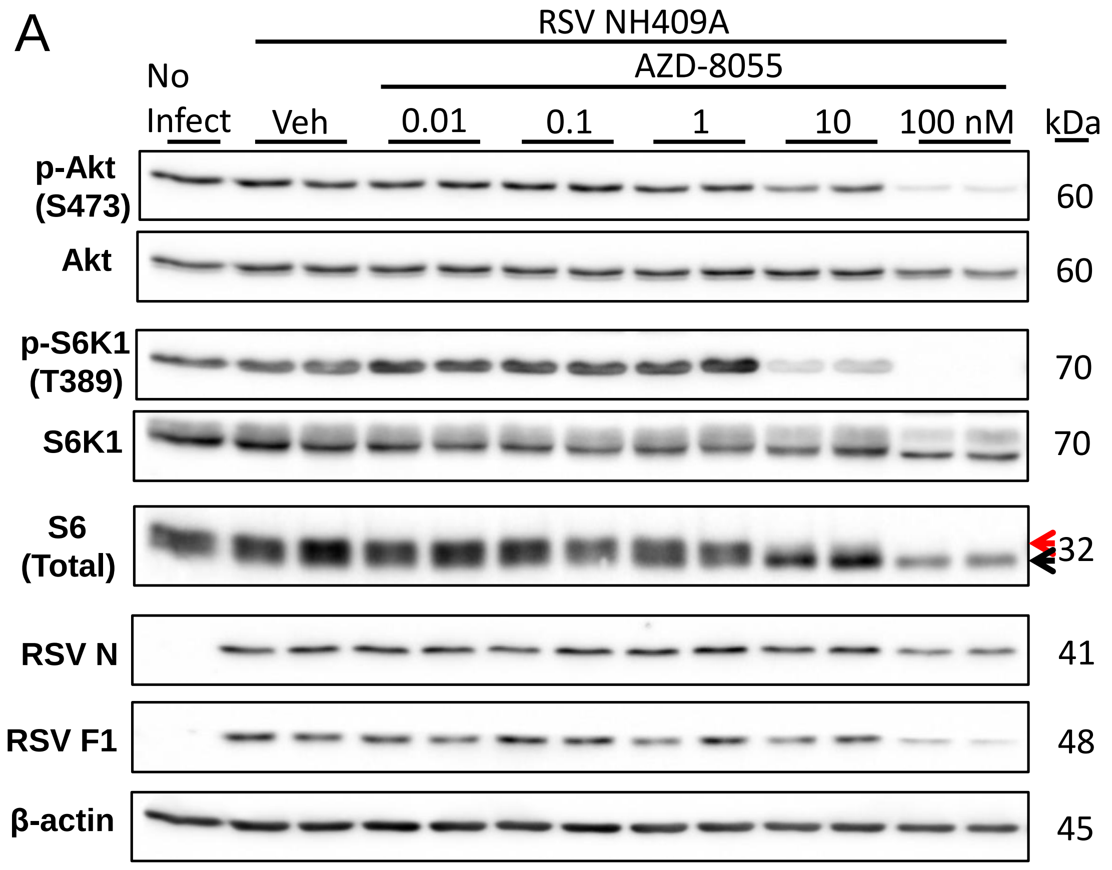

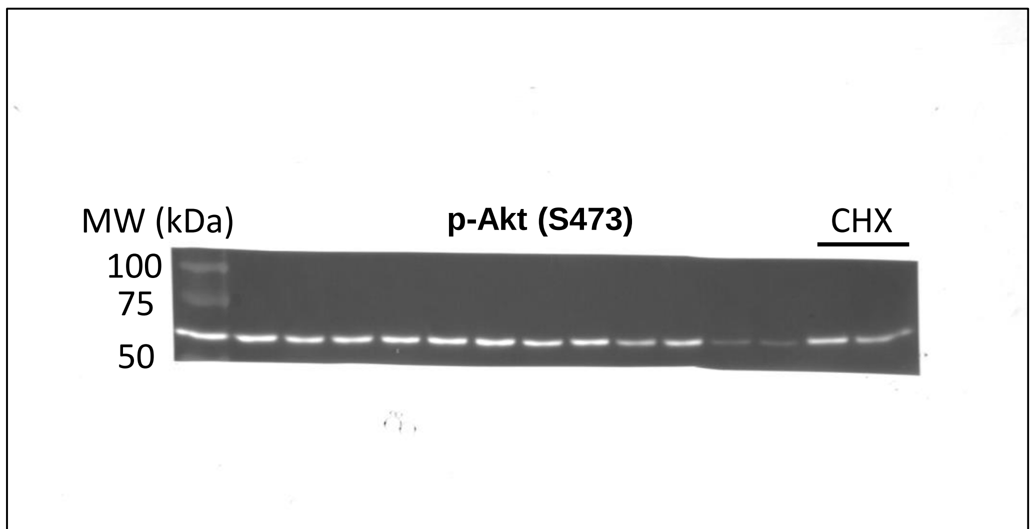

CHX: cycloheximide (500 nM as positive control)

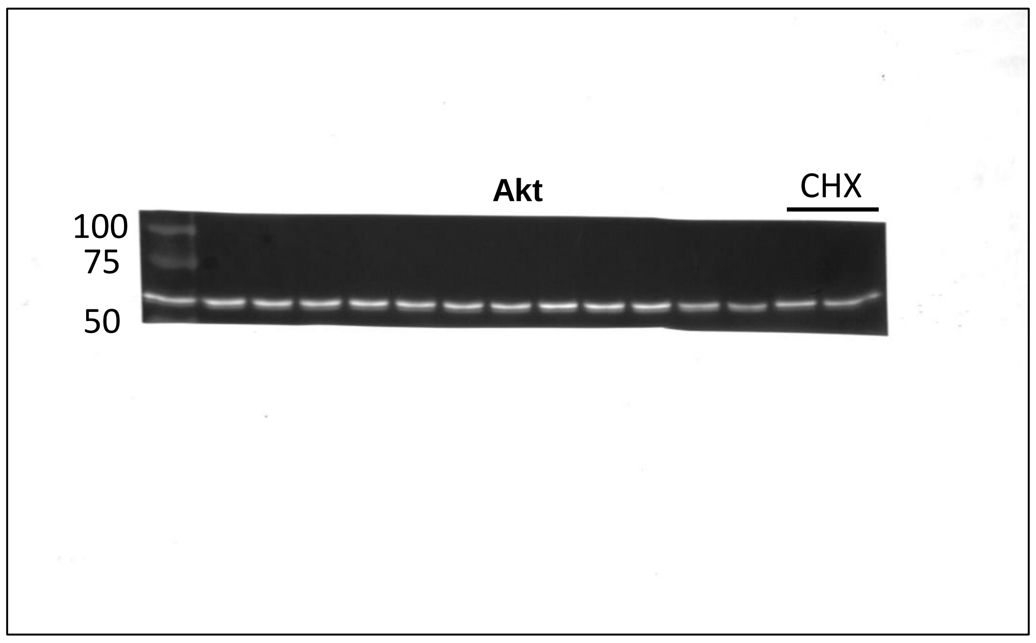

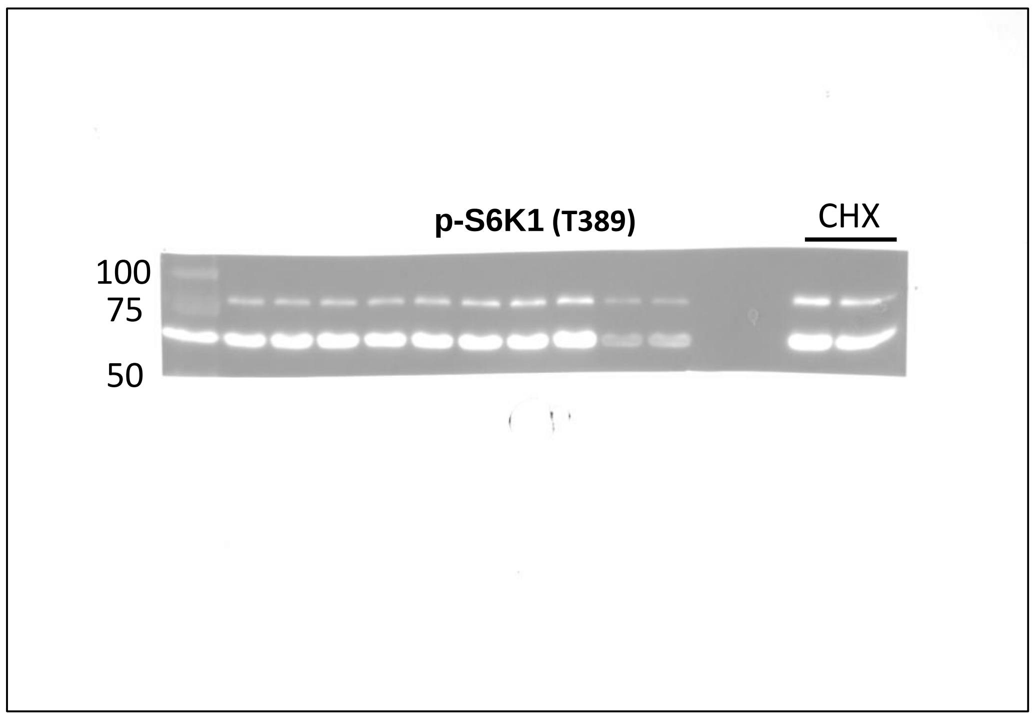

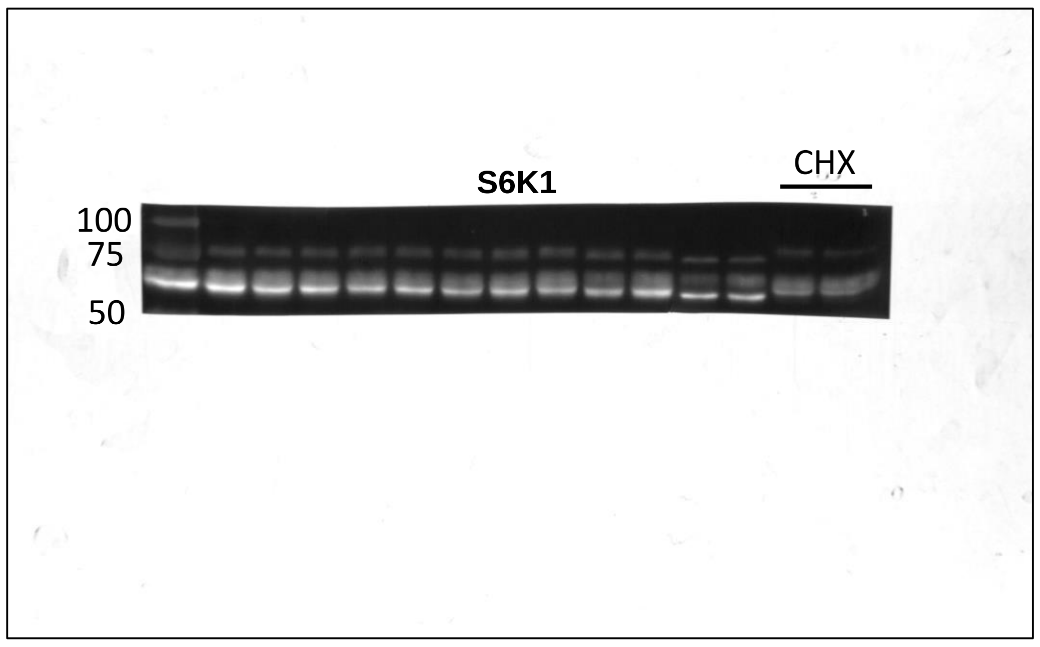

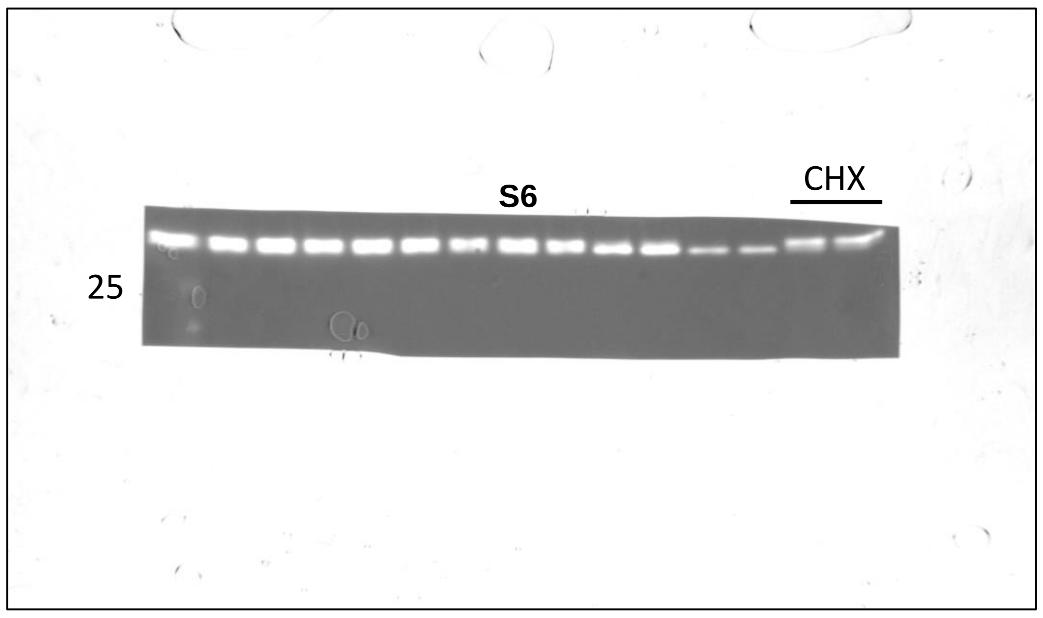

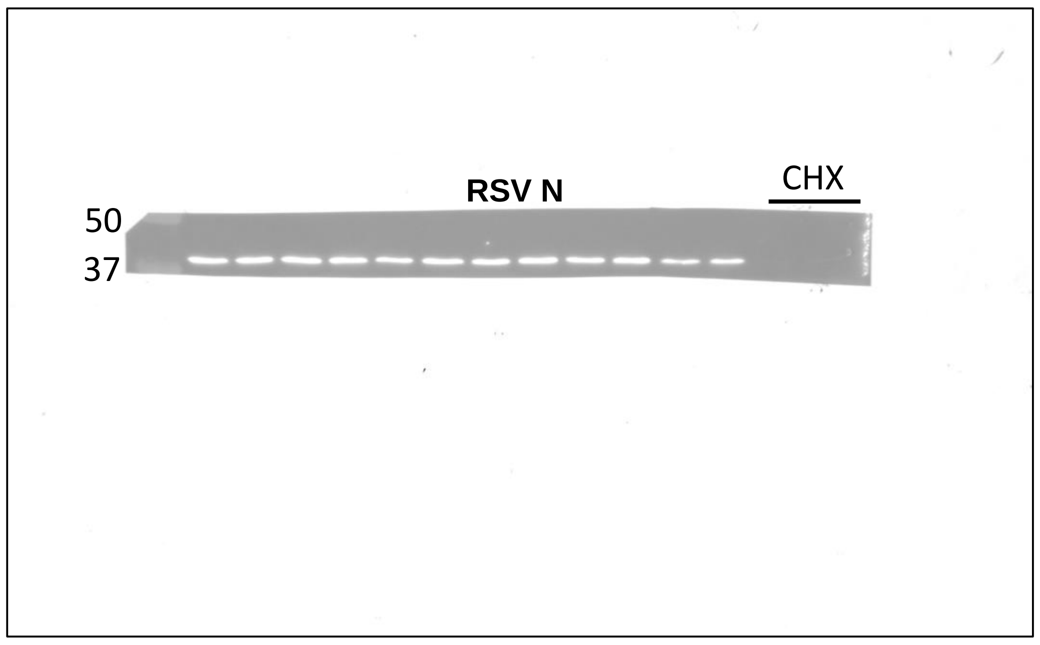

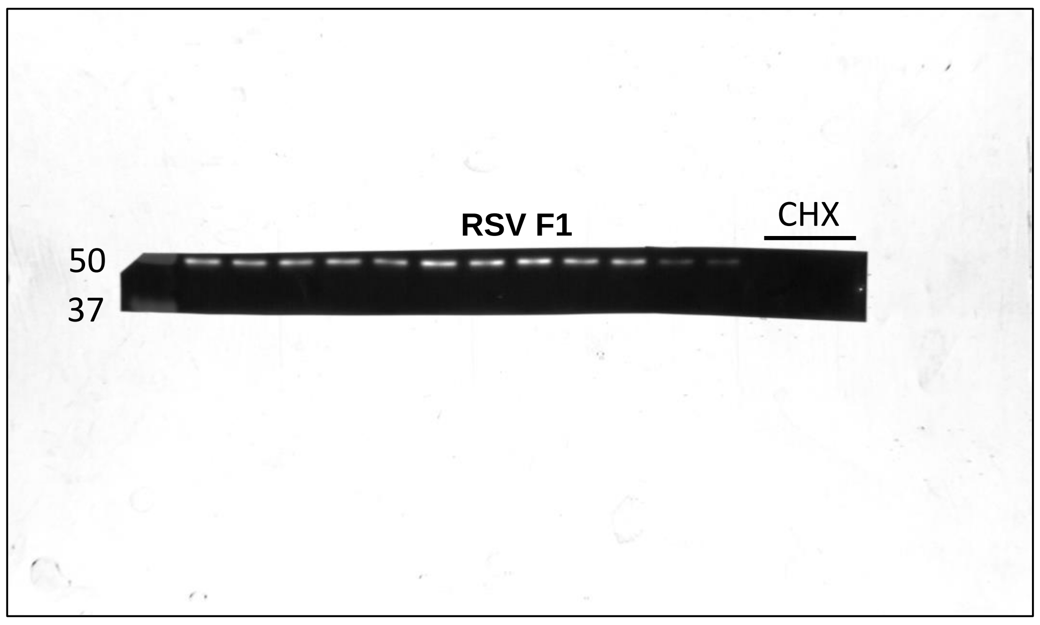

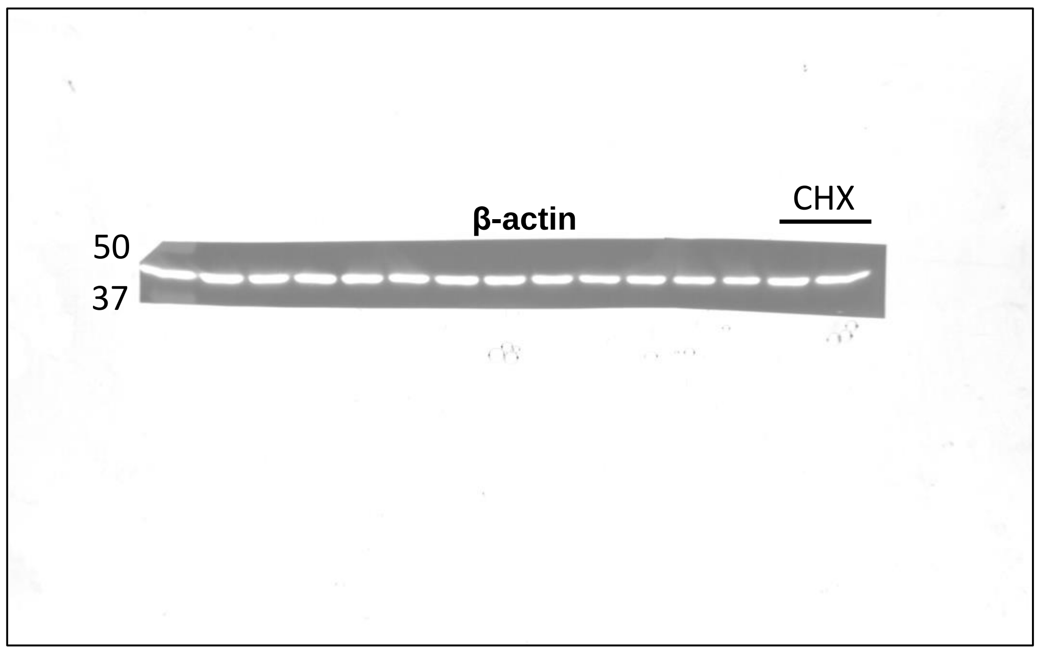

Raw Images of Western Blot for Figure 7A

A

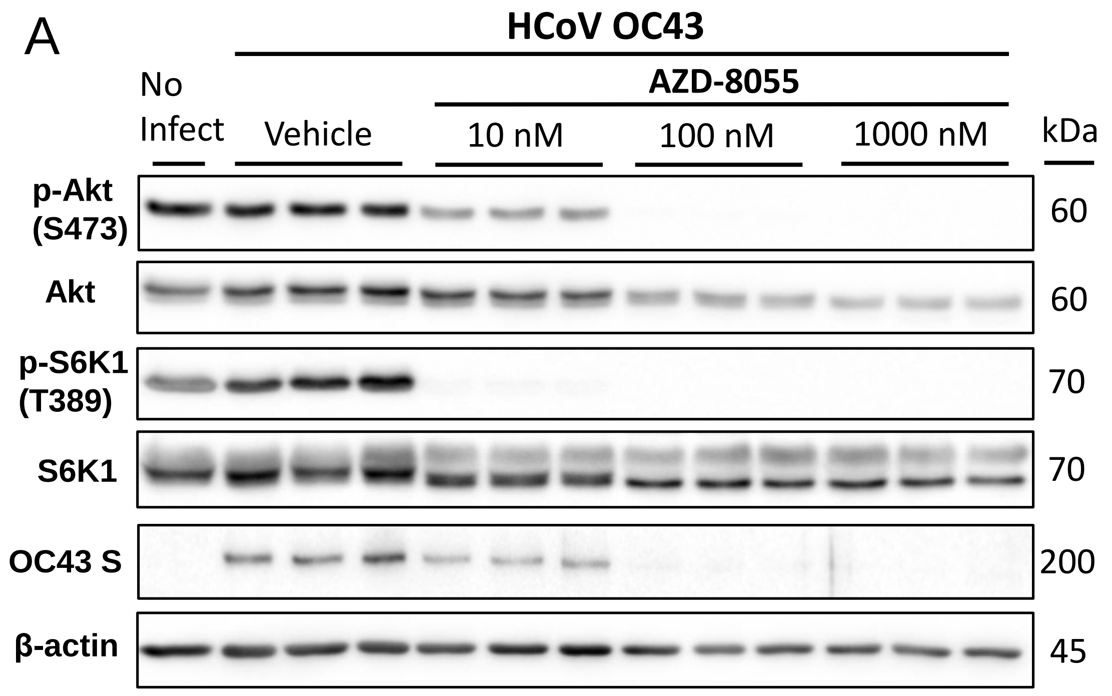

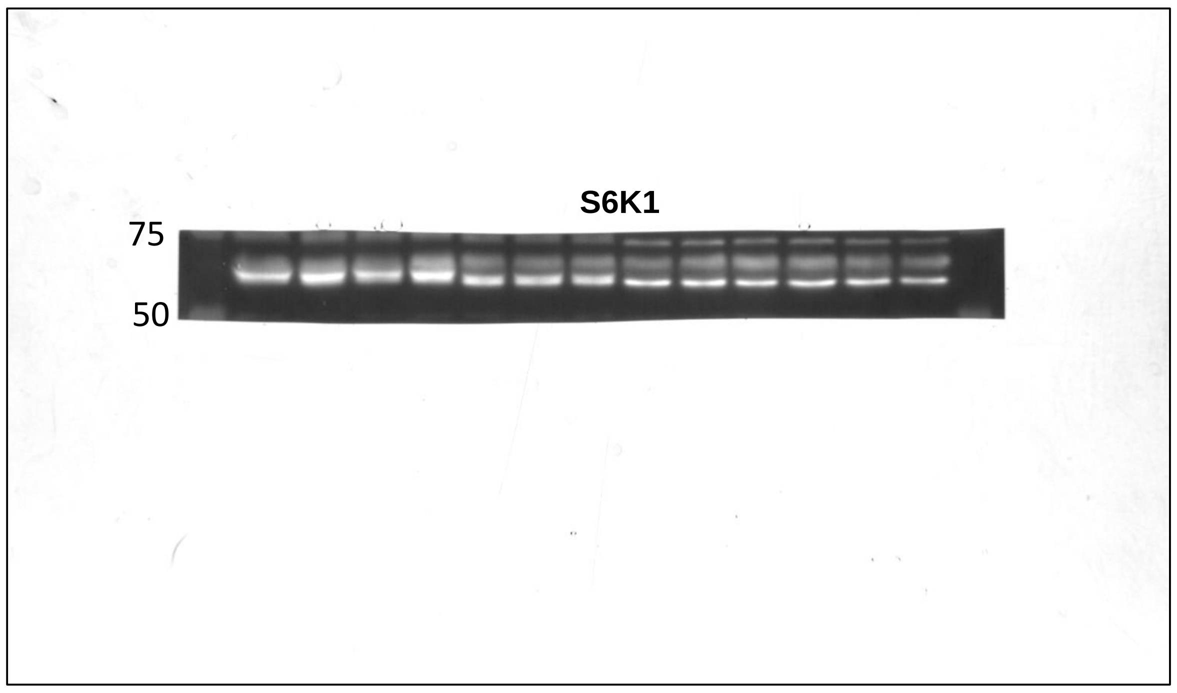

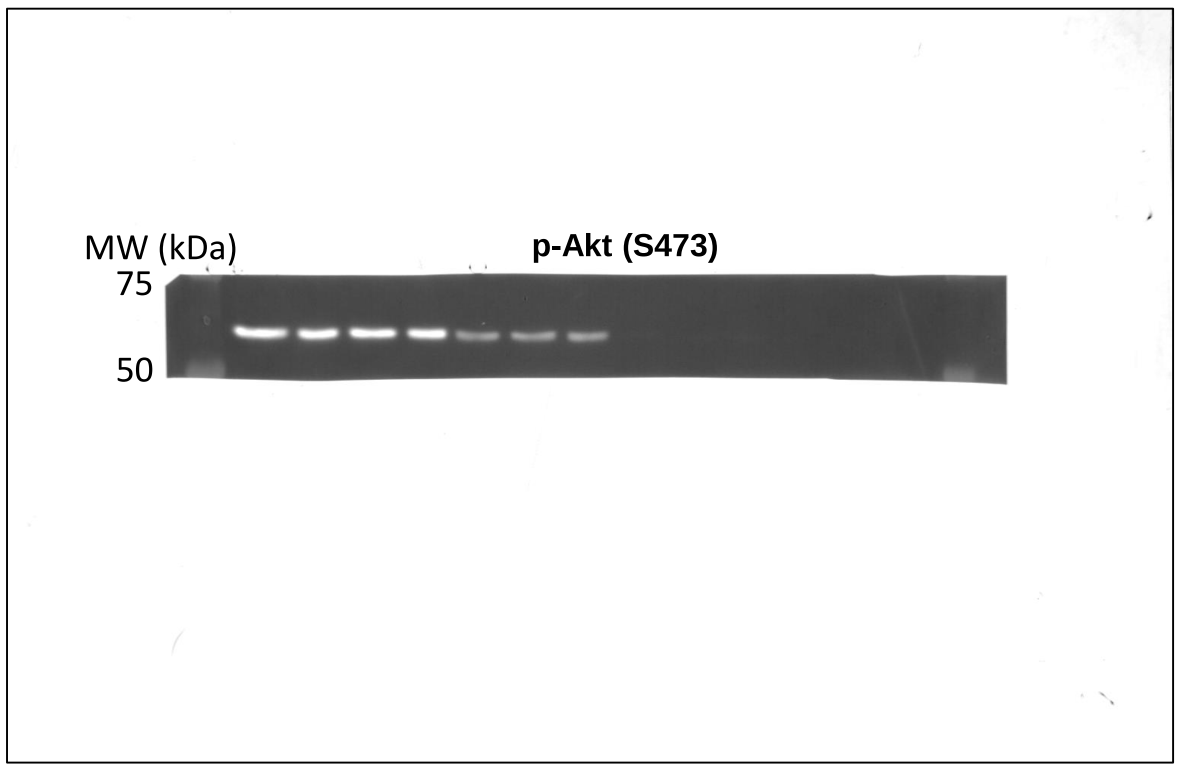

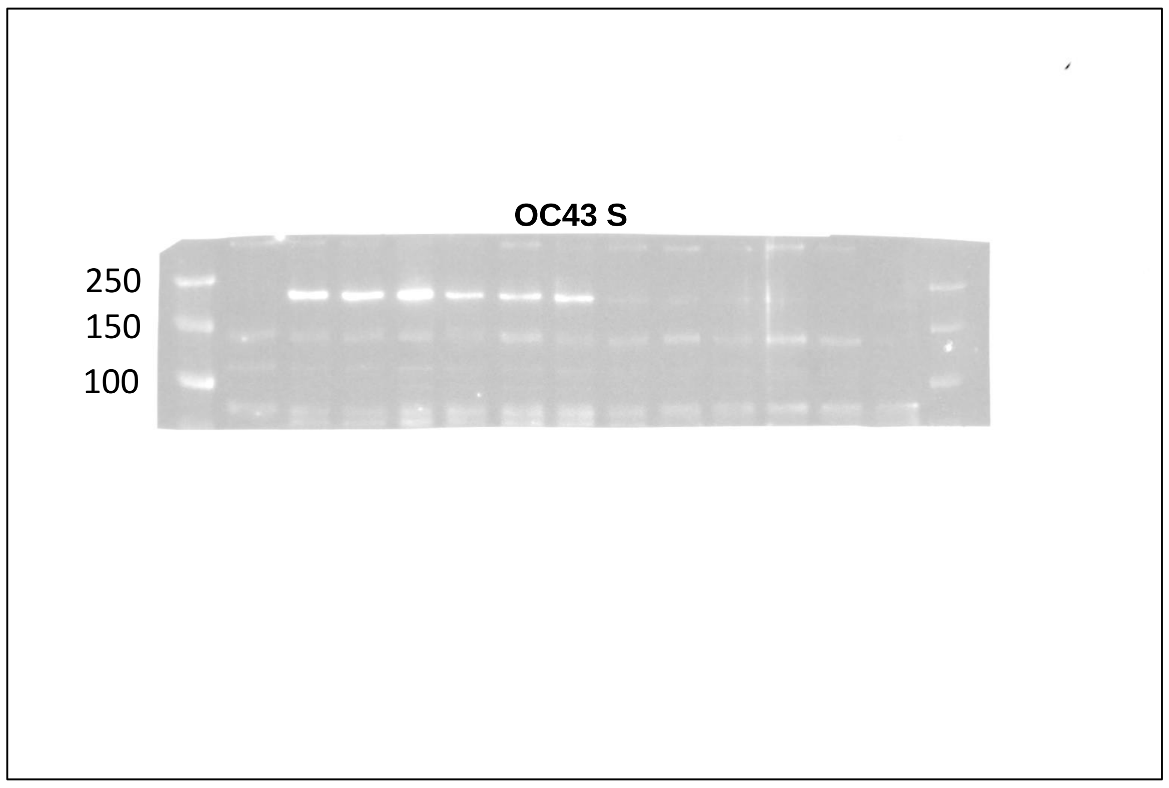

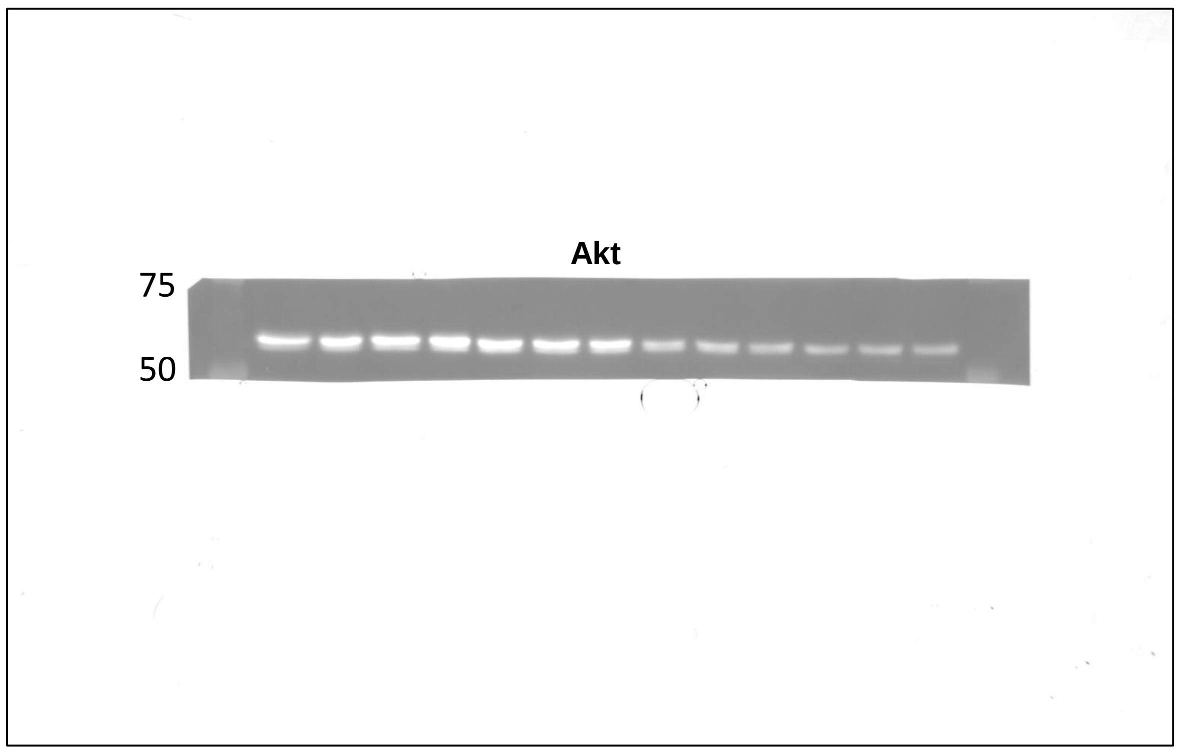

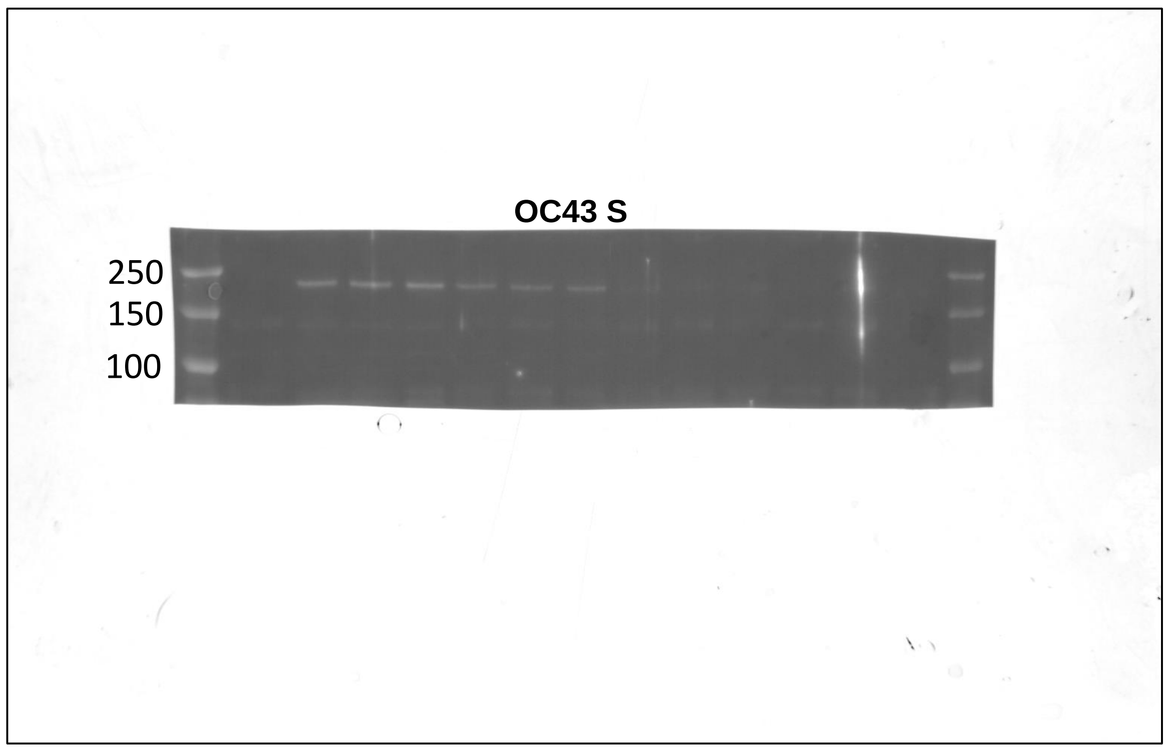

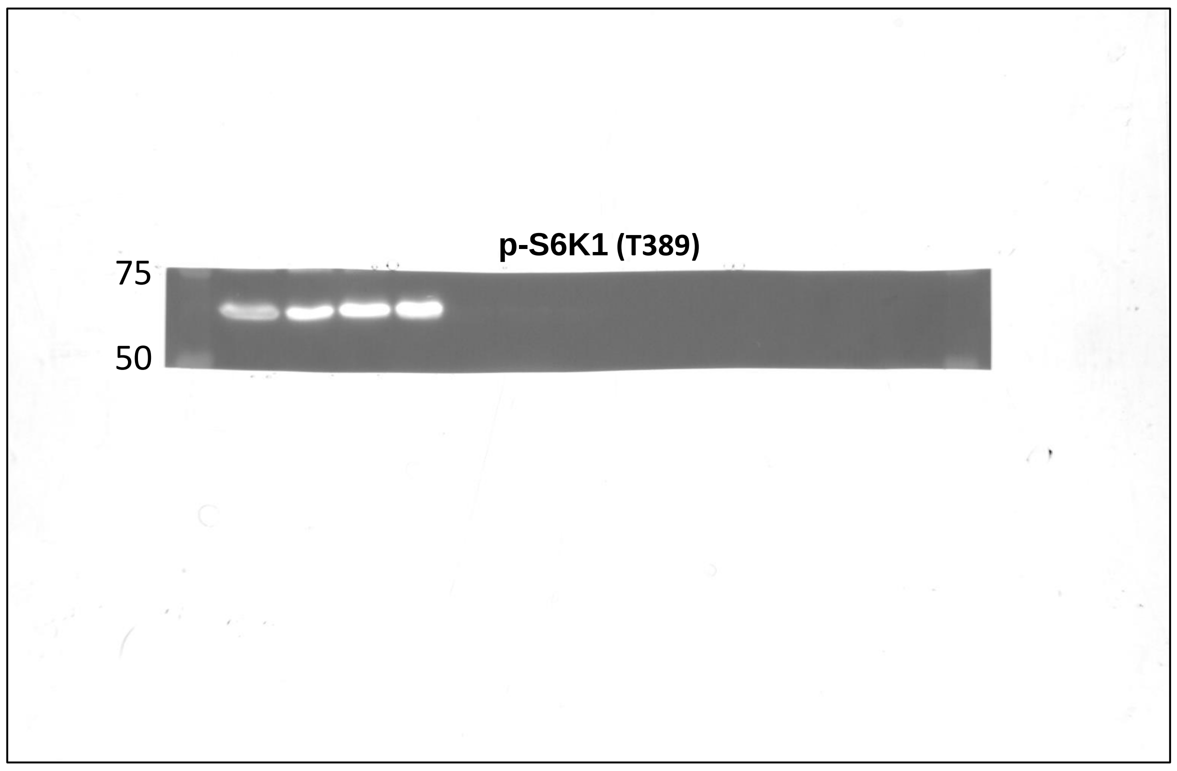

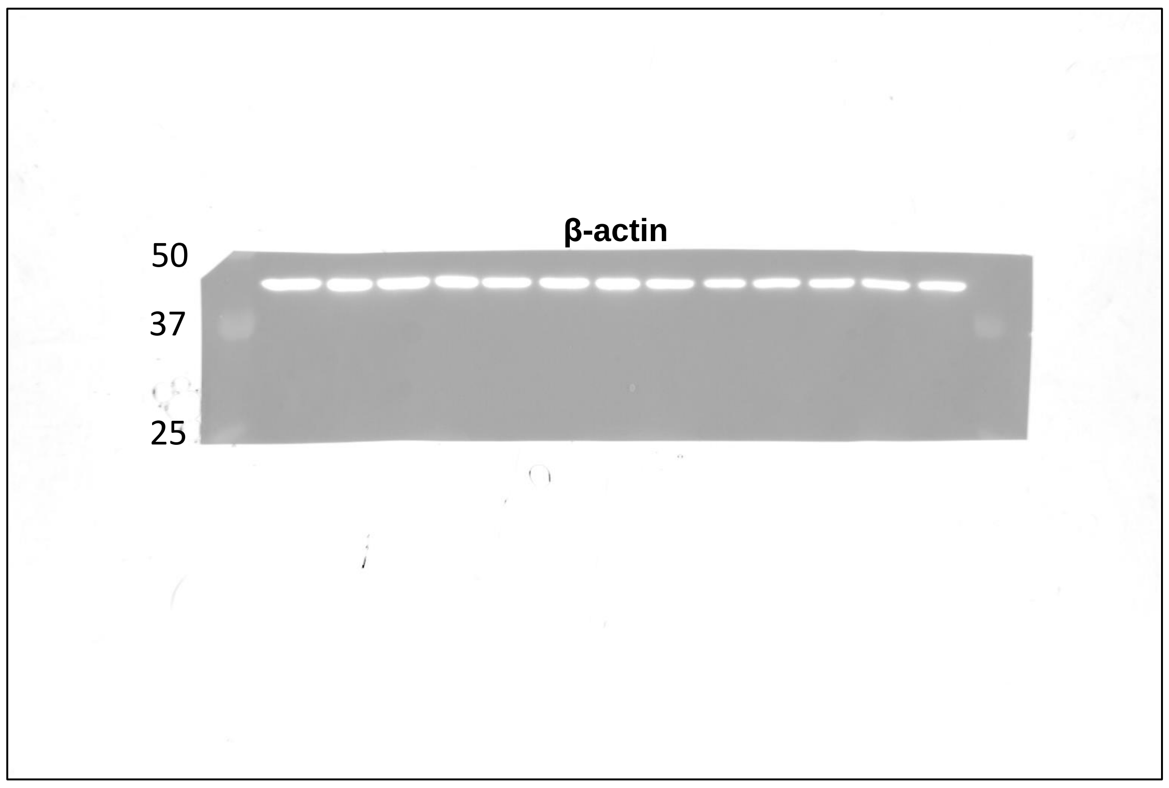

Raw Images of Western Blot for Figure 7C

Huynh et al

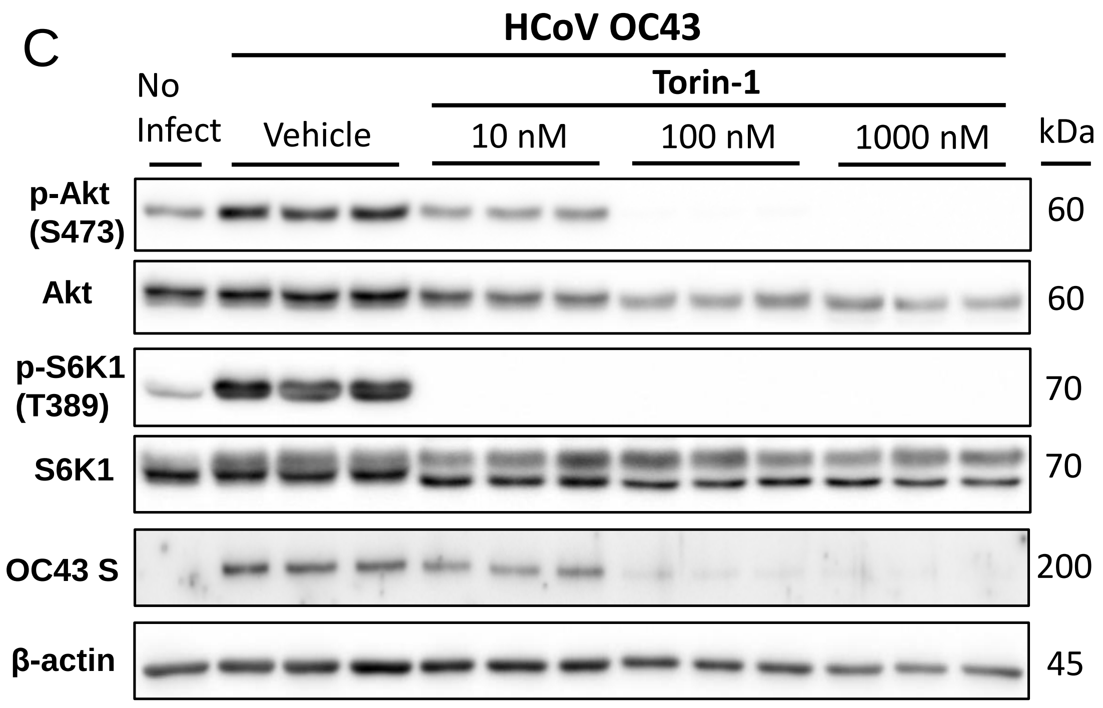

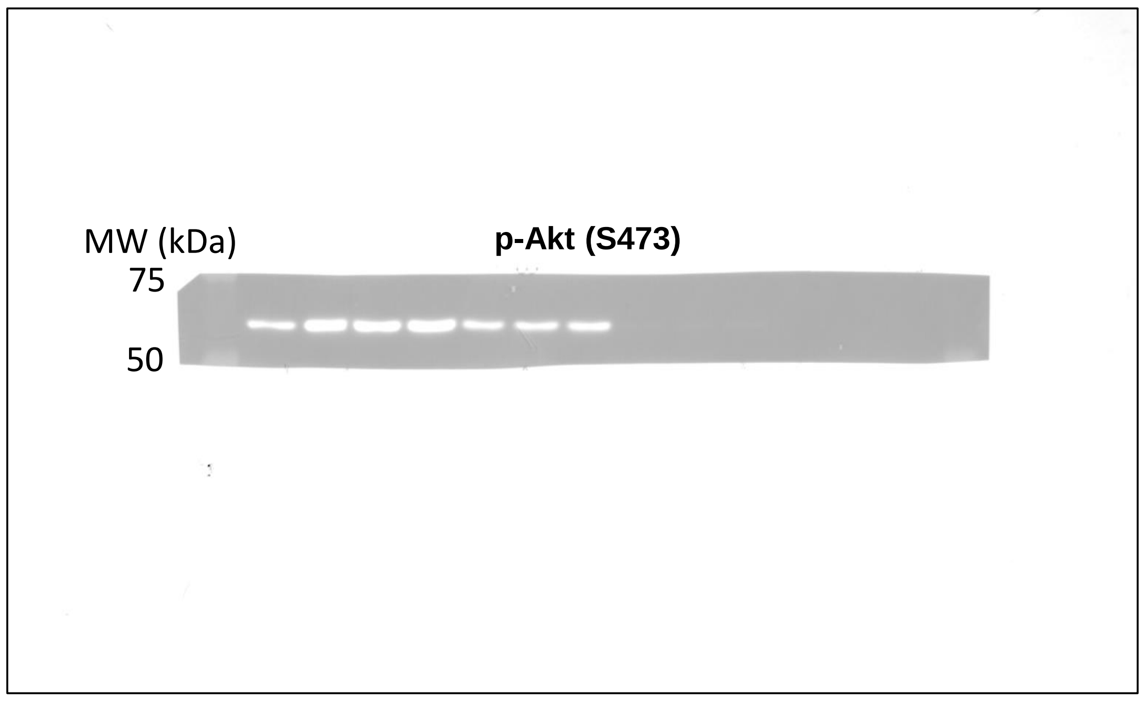

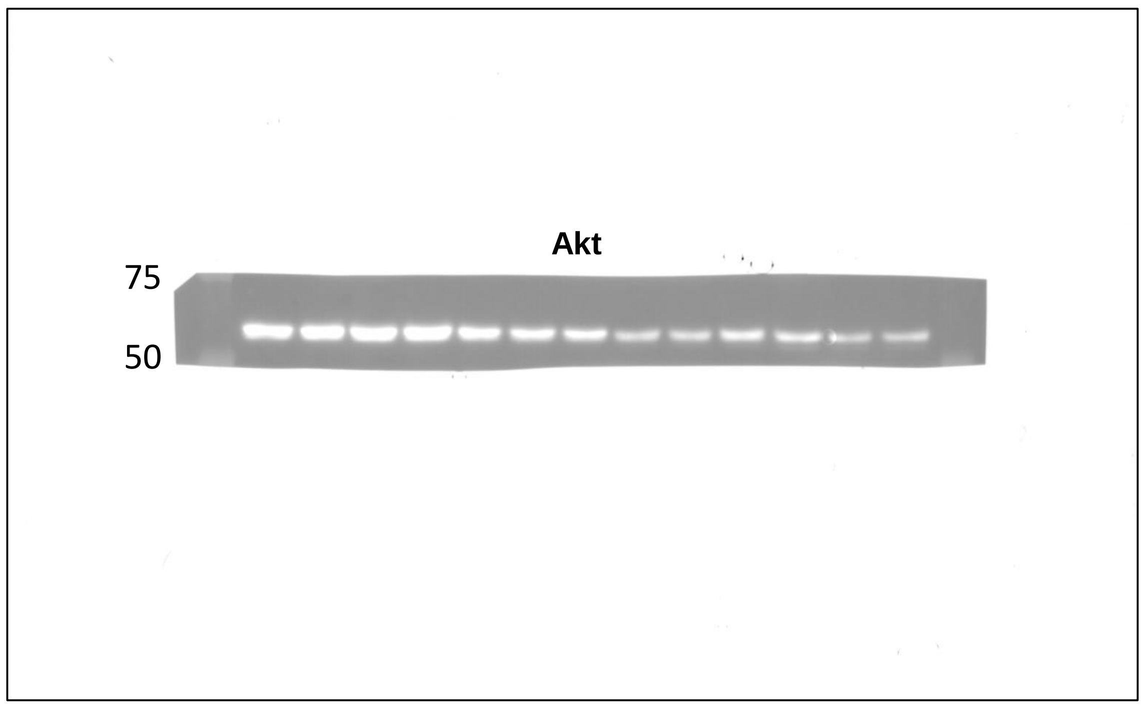

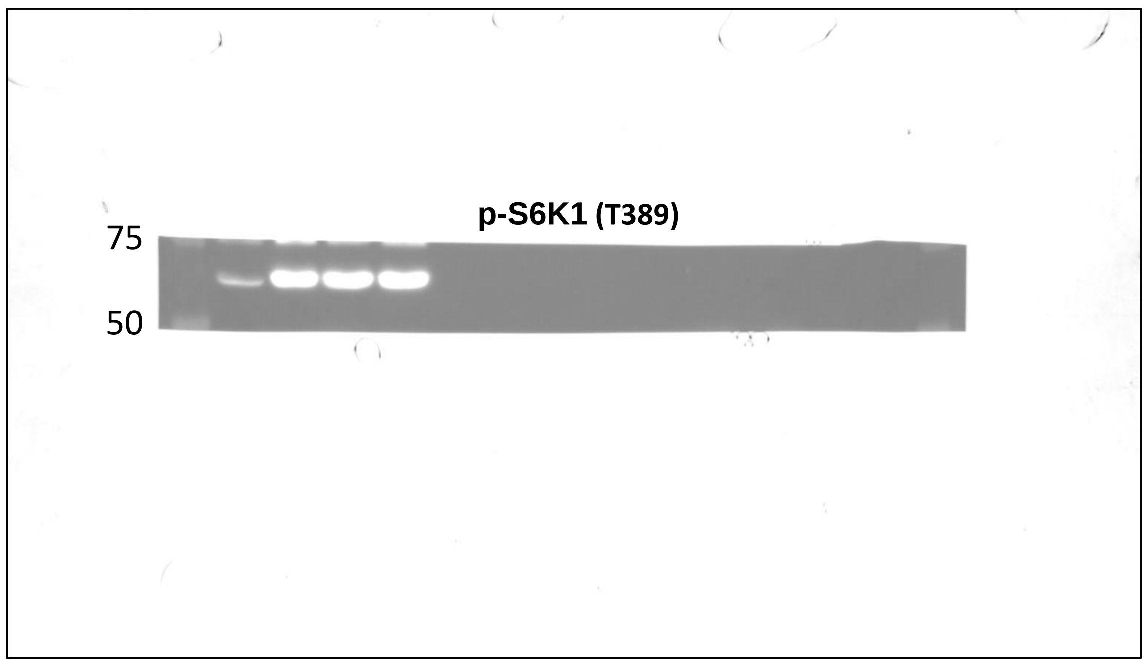

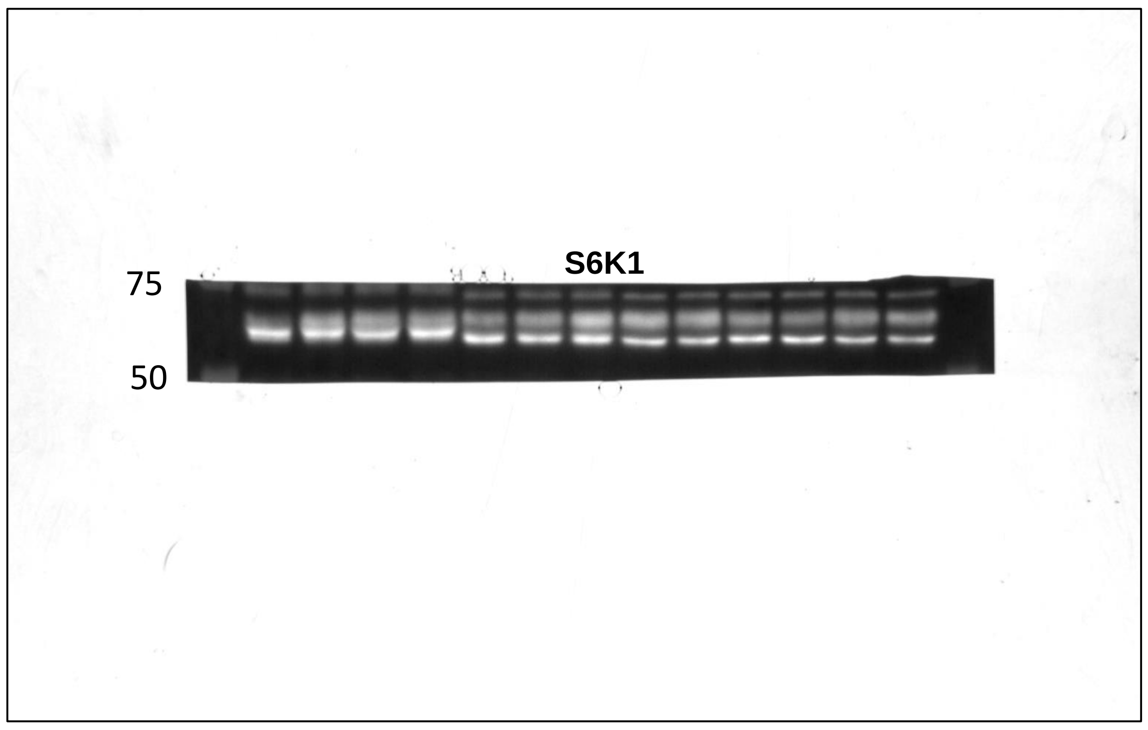

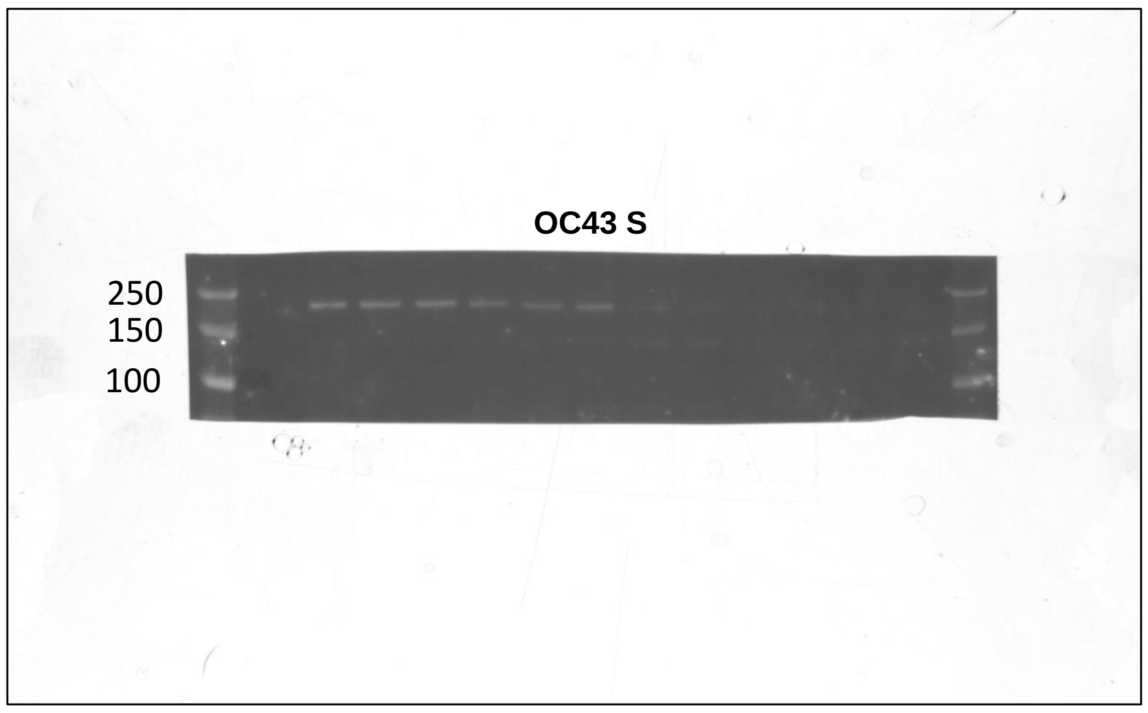

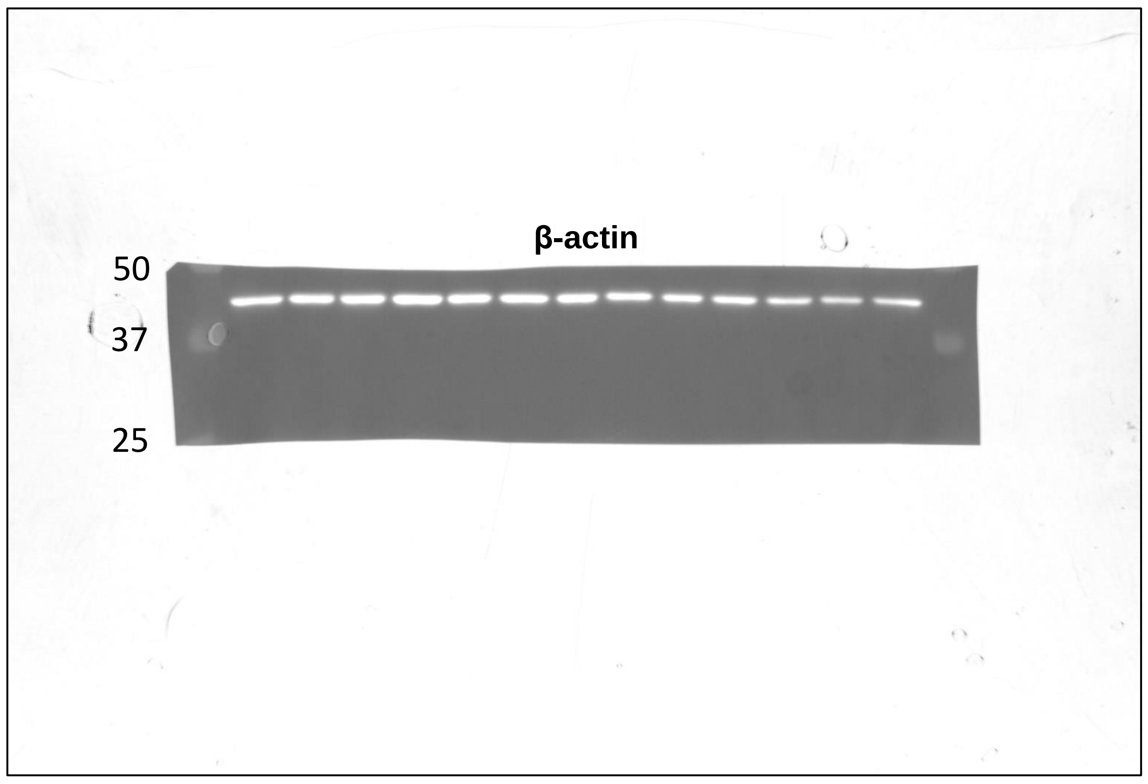

Raw Images of Western Blot for Figure 7E

Huynh et al

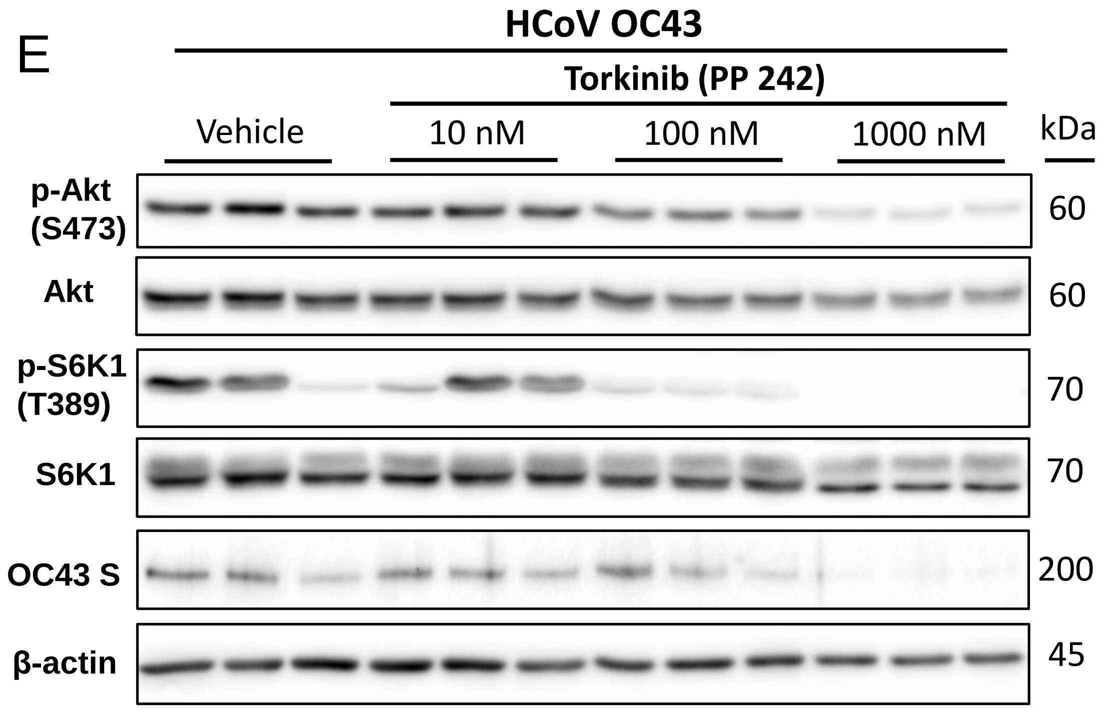

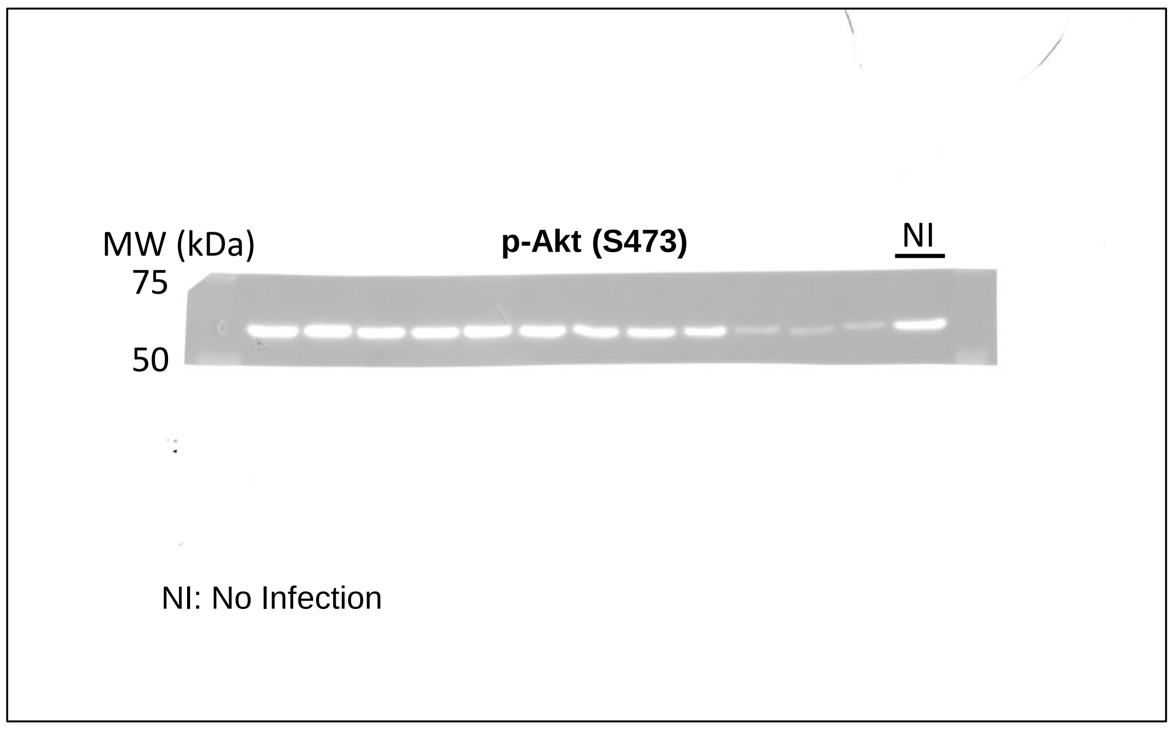

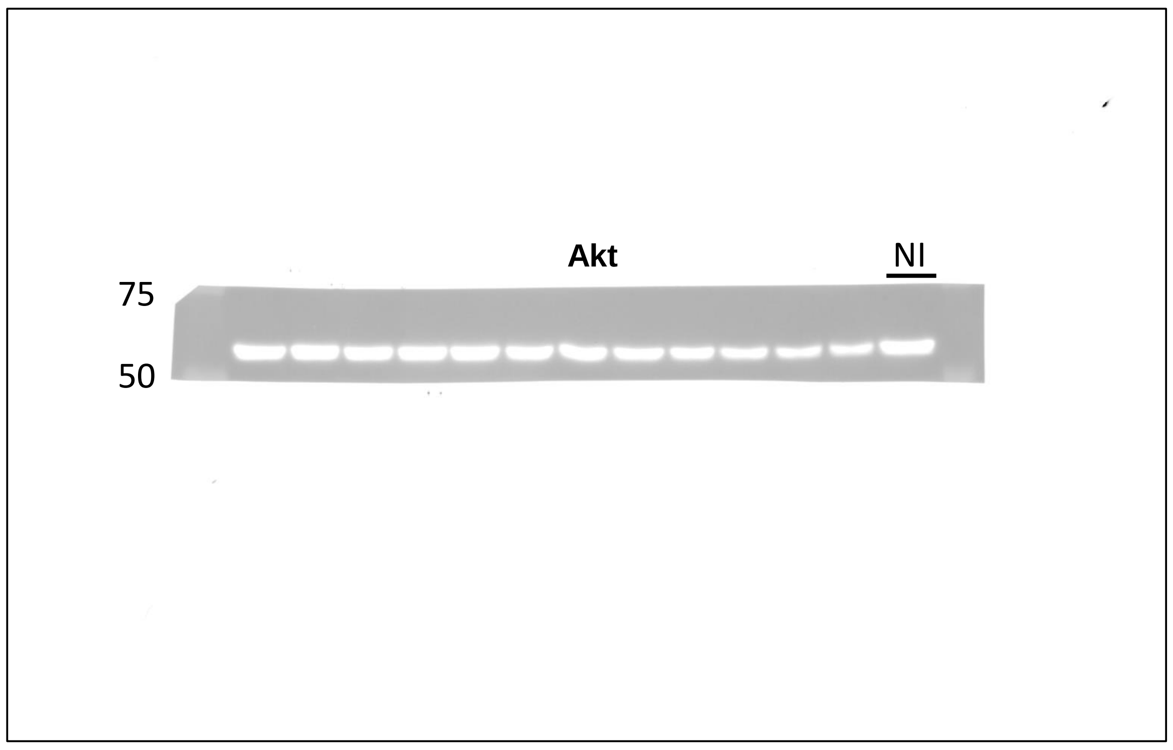

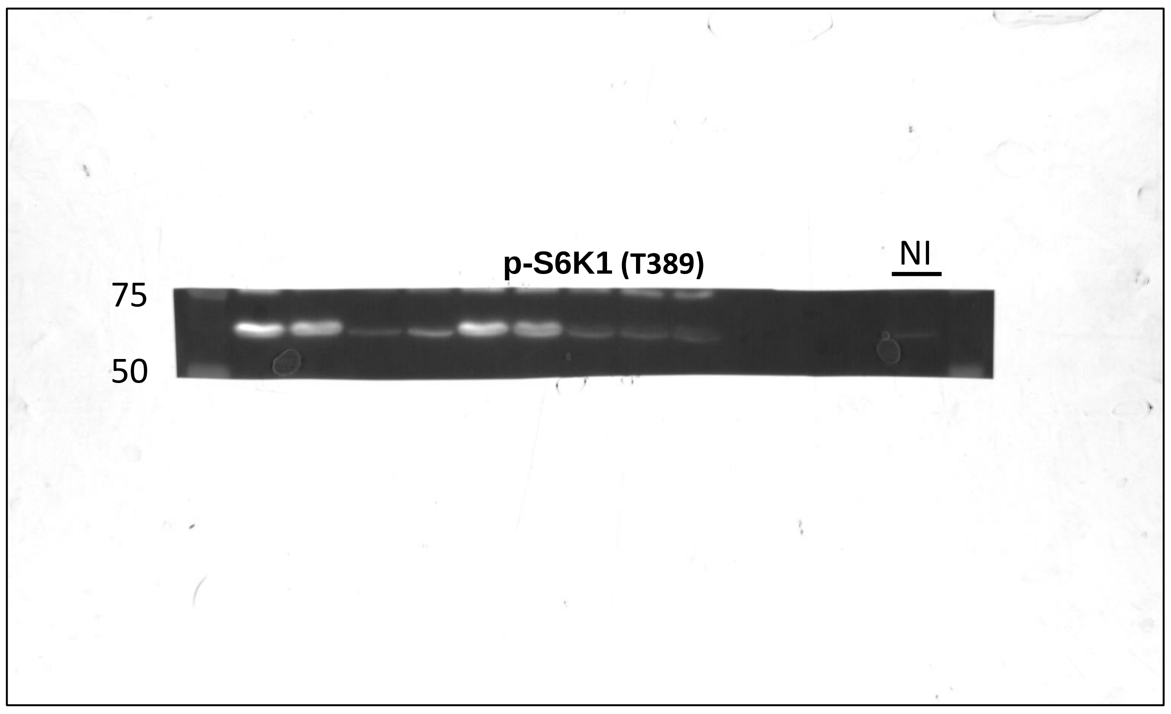

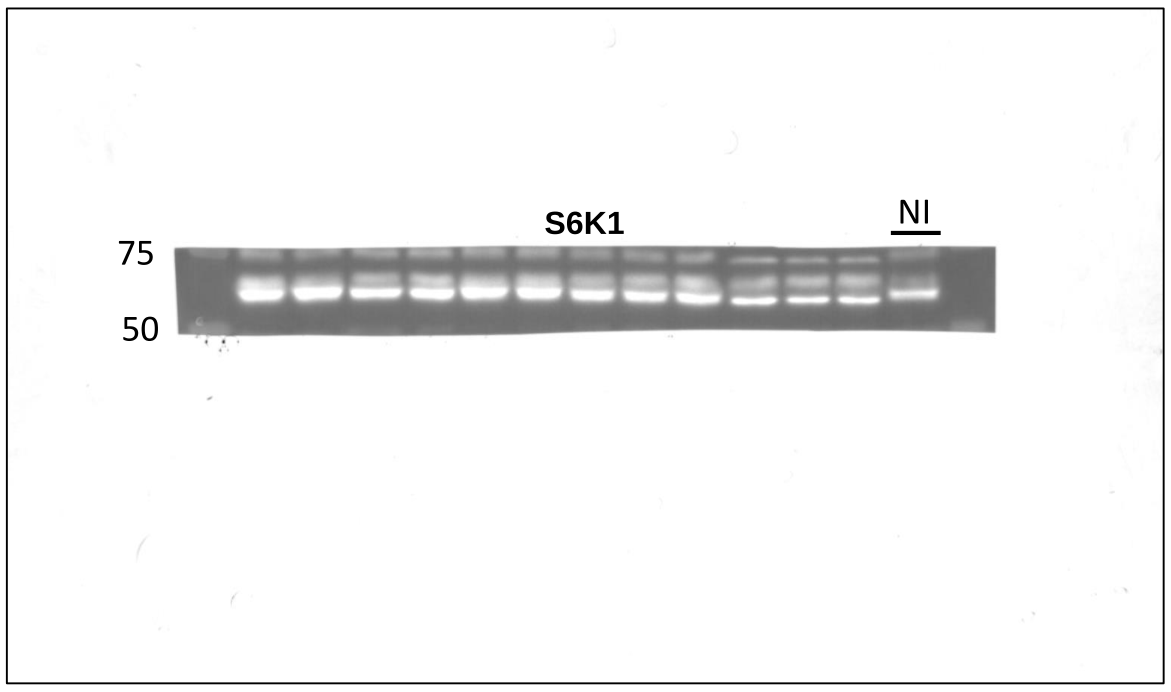

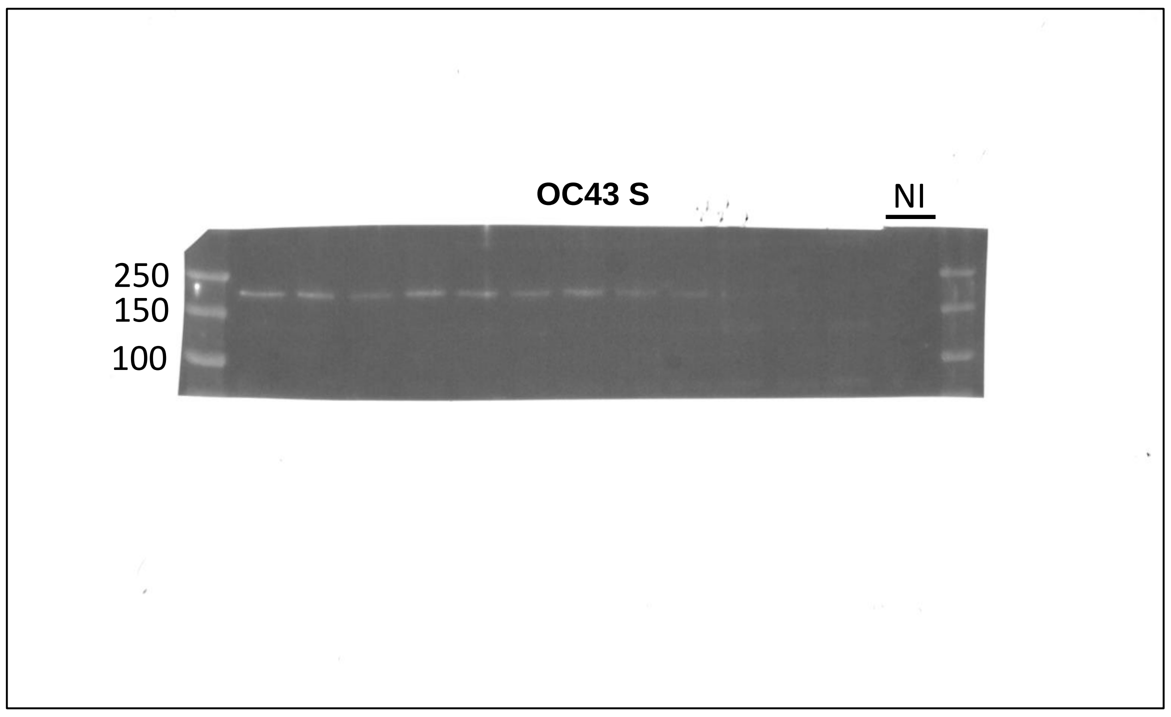

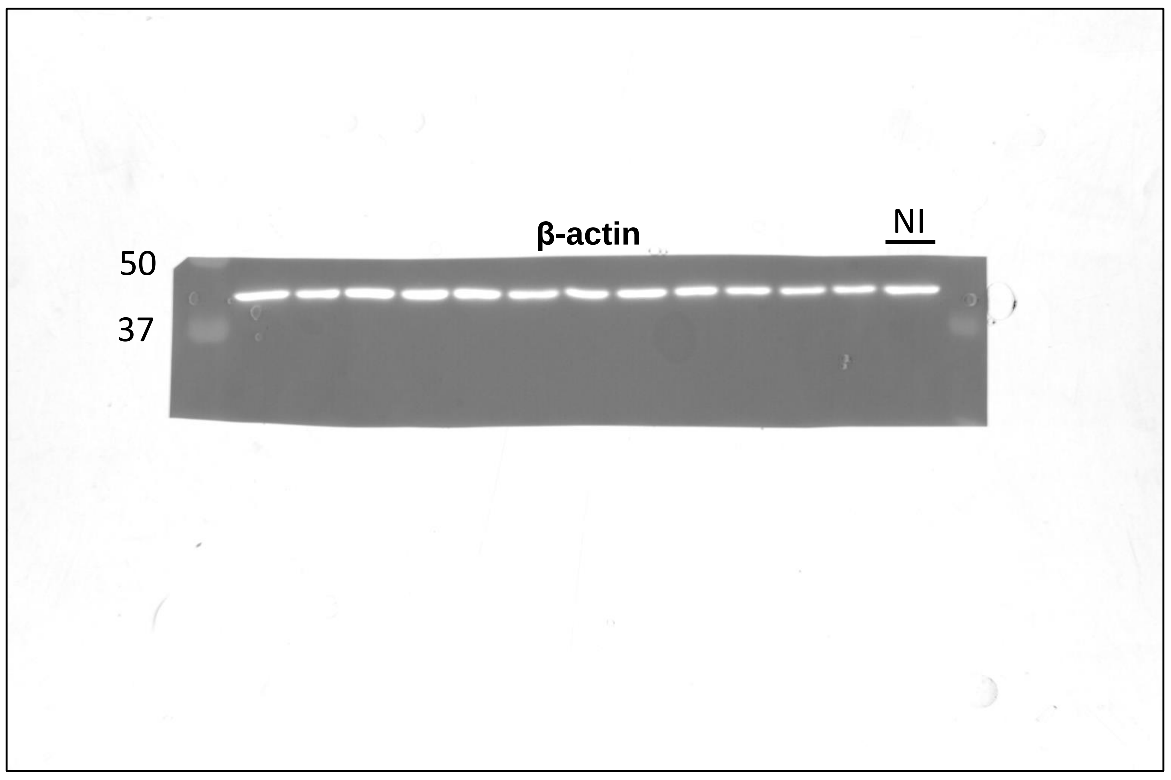

Raw Images of Western Blot for Supplementary Figure 1A

Huynh et al

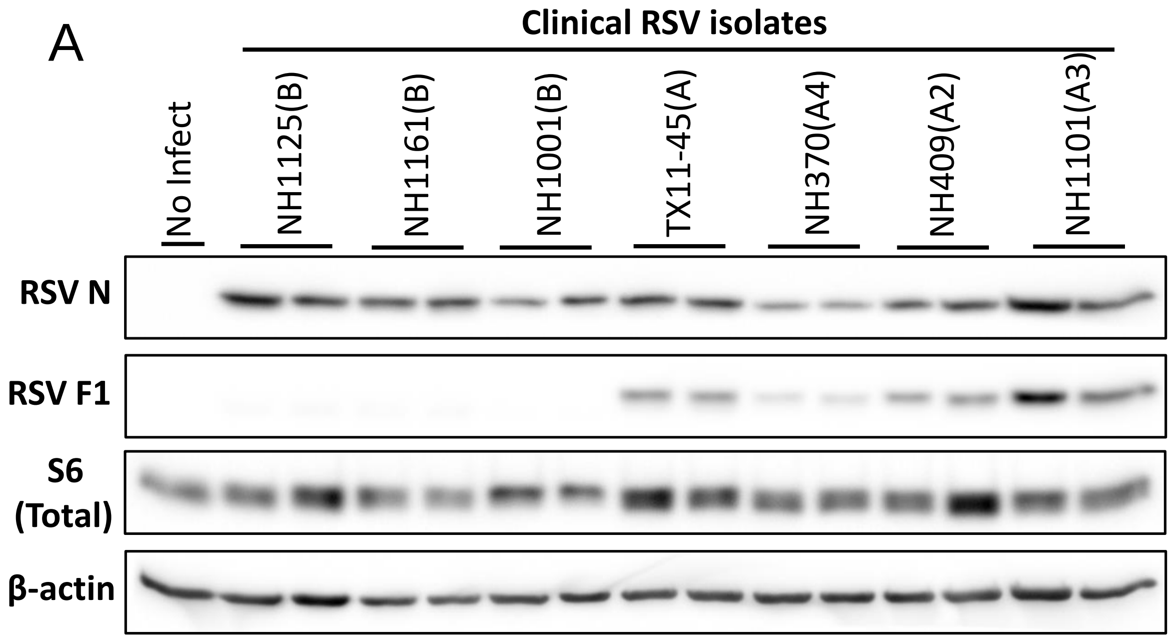

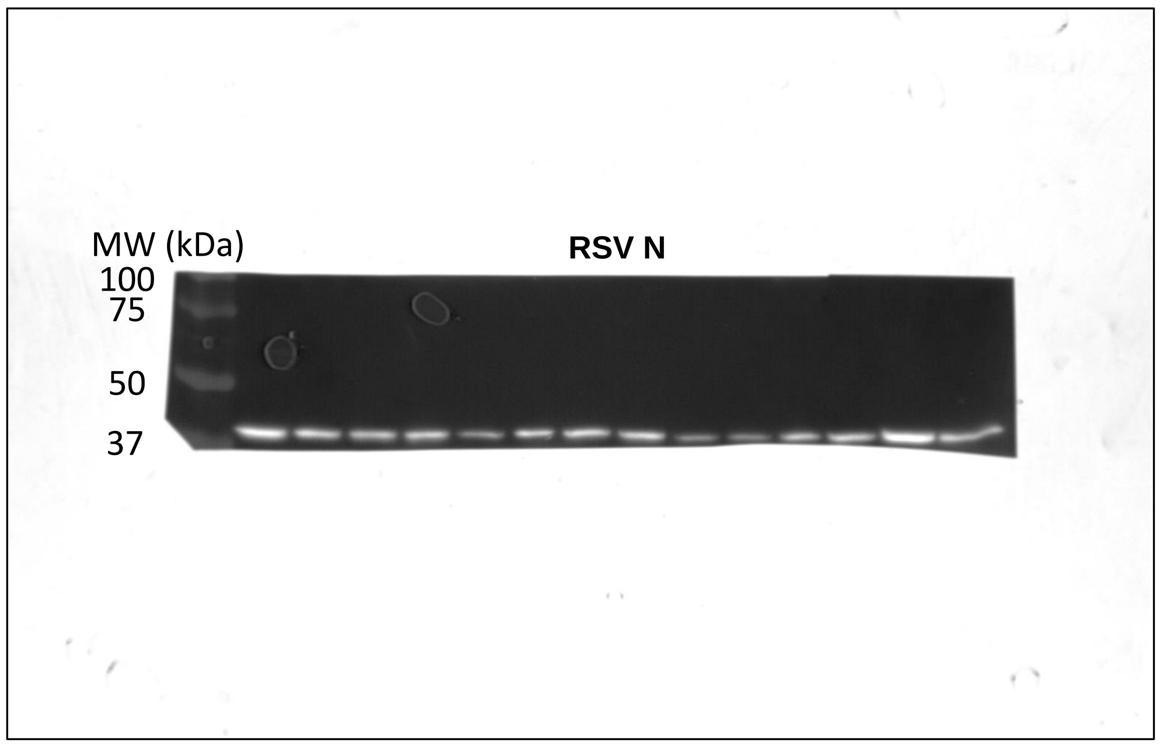

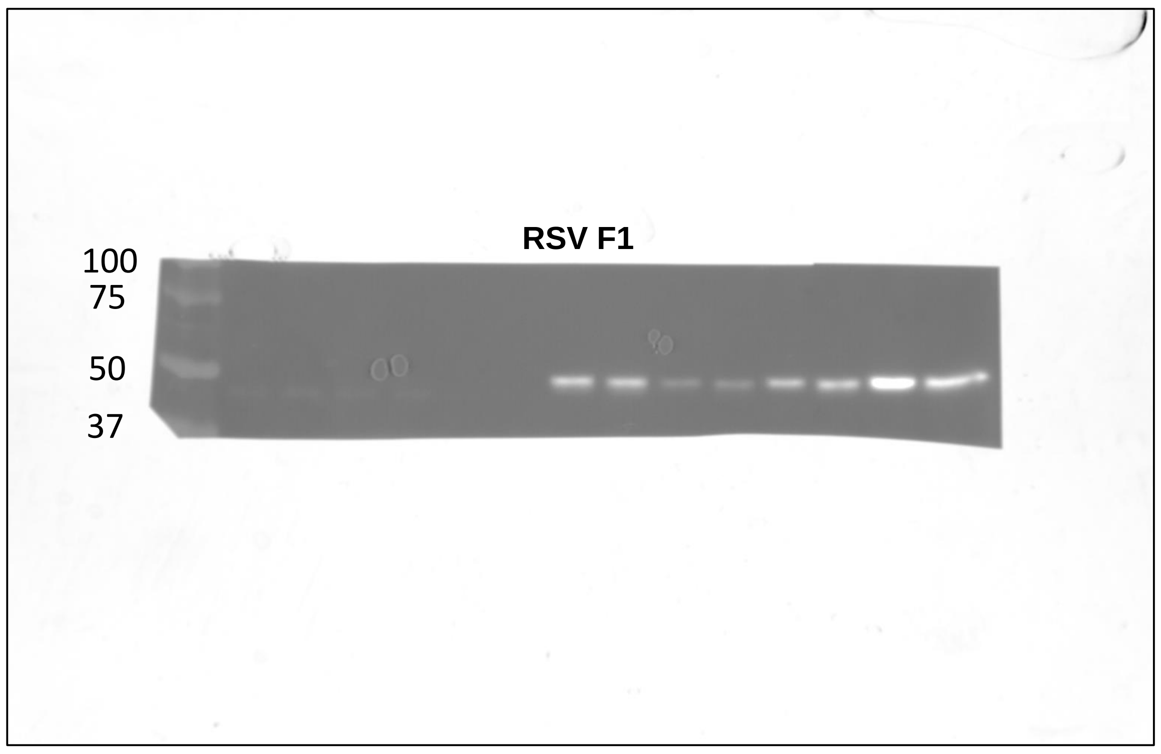

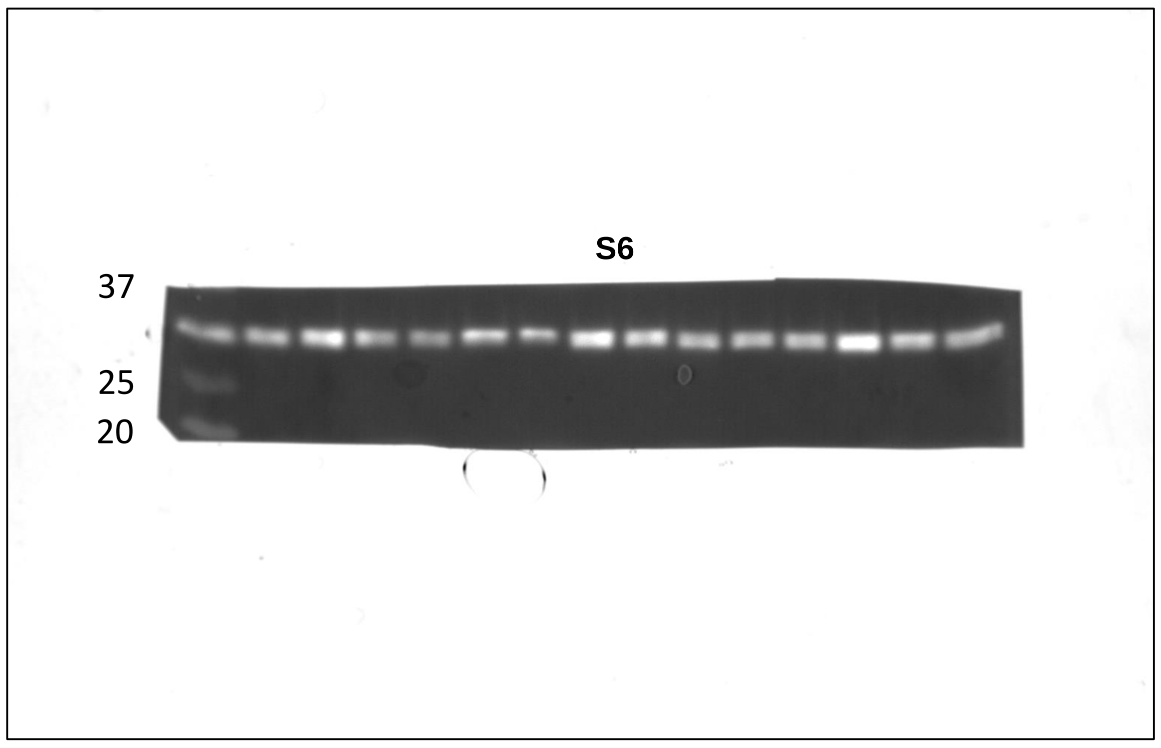

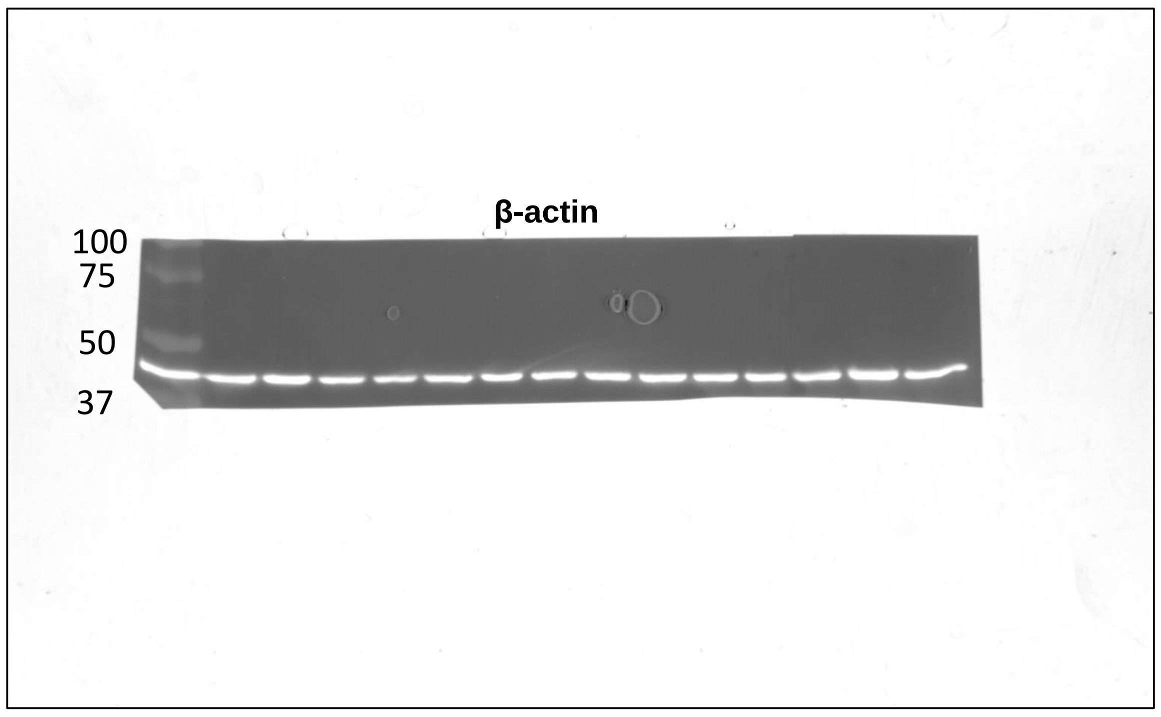

Raw Images of Western Blot for Supplementary Figure 2A

Huynh et al

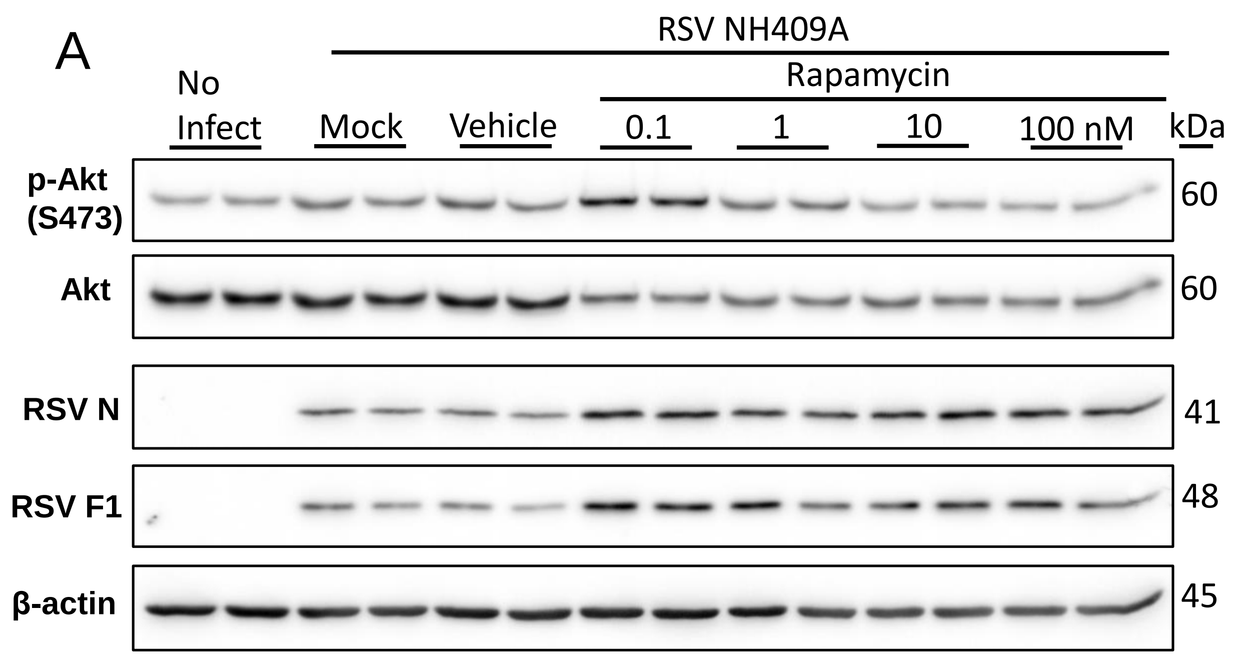

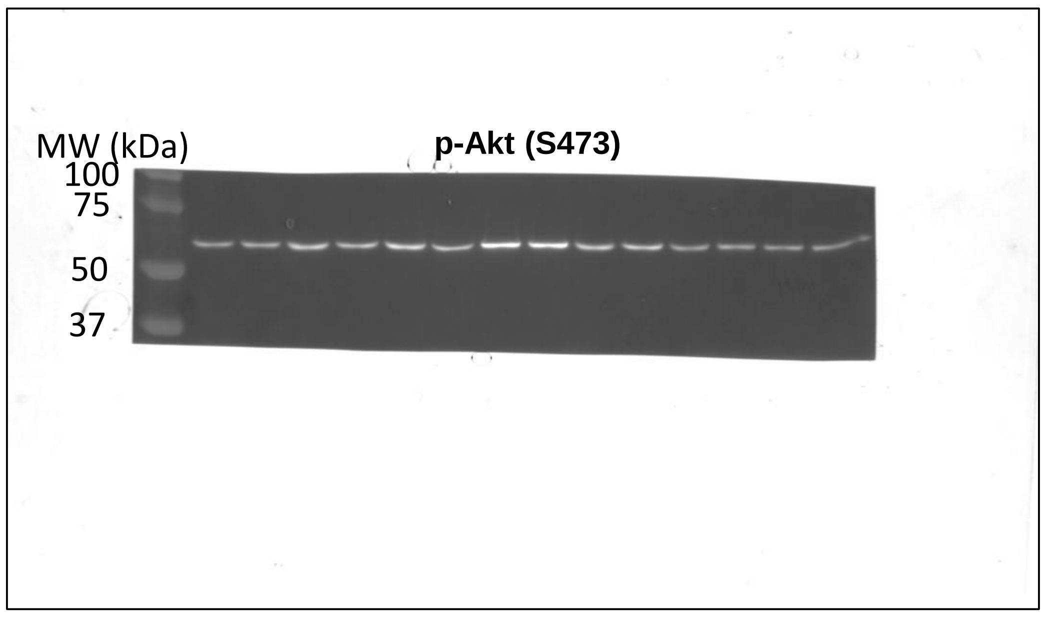

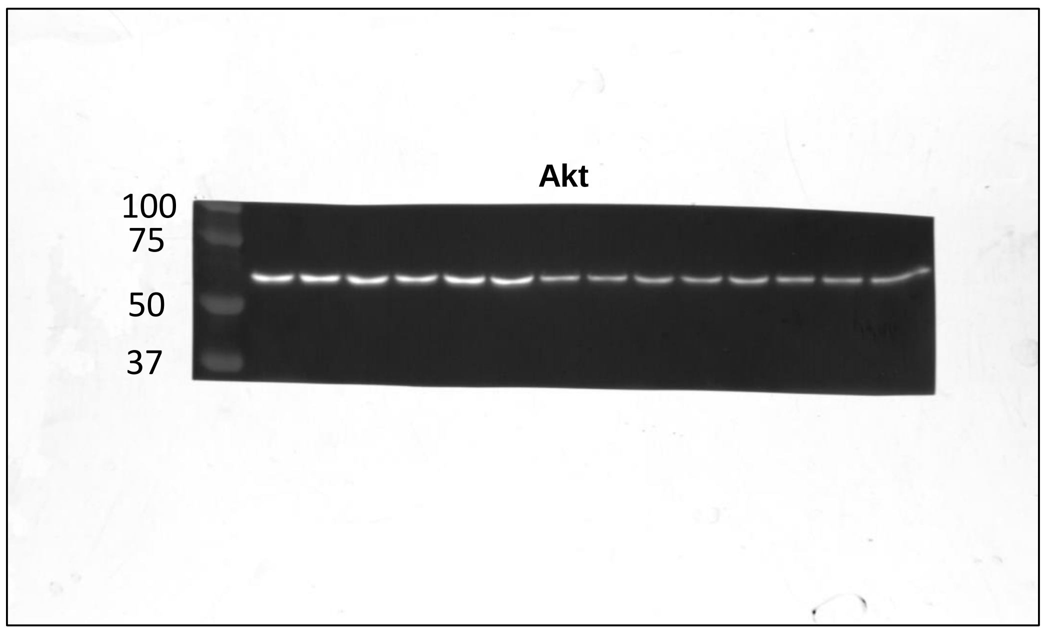

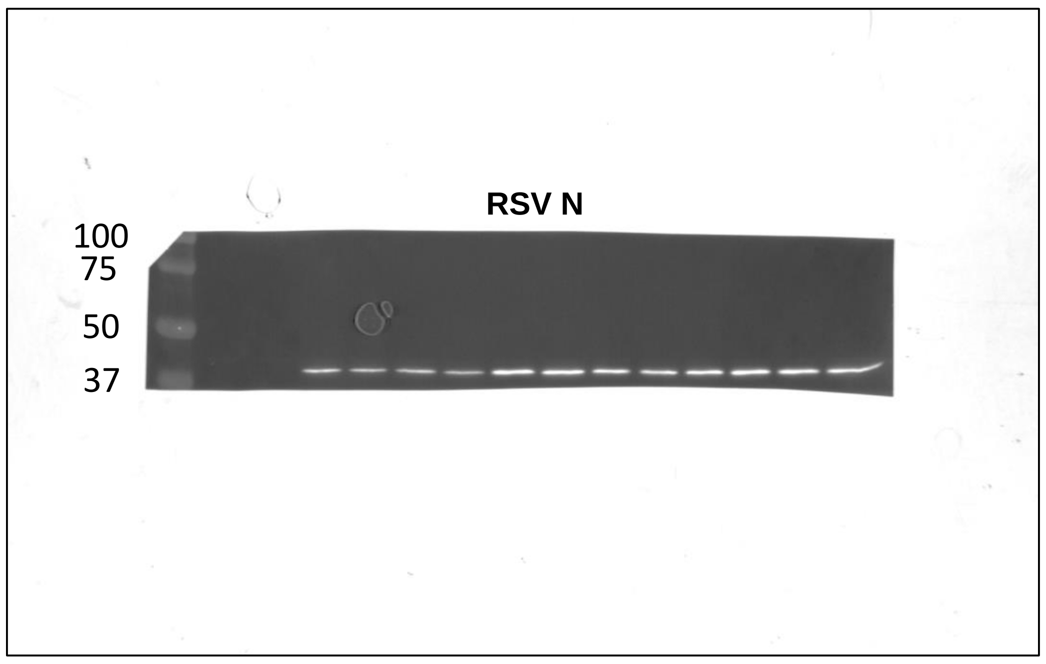

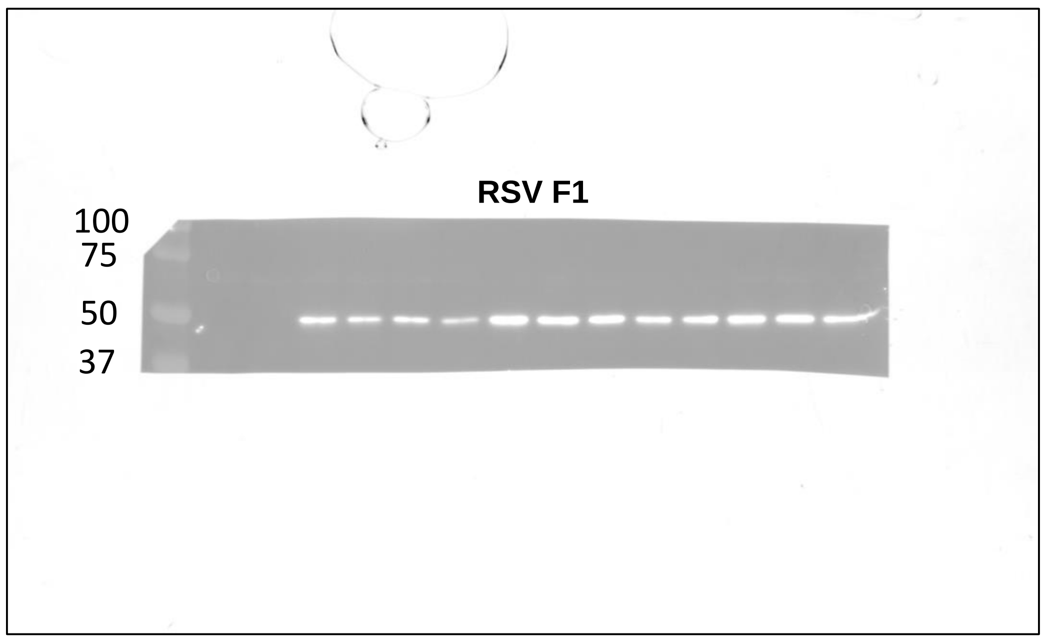

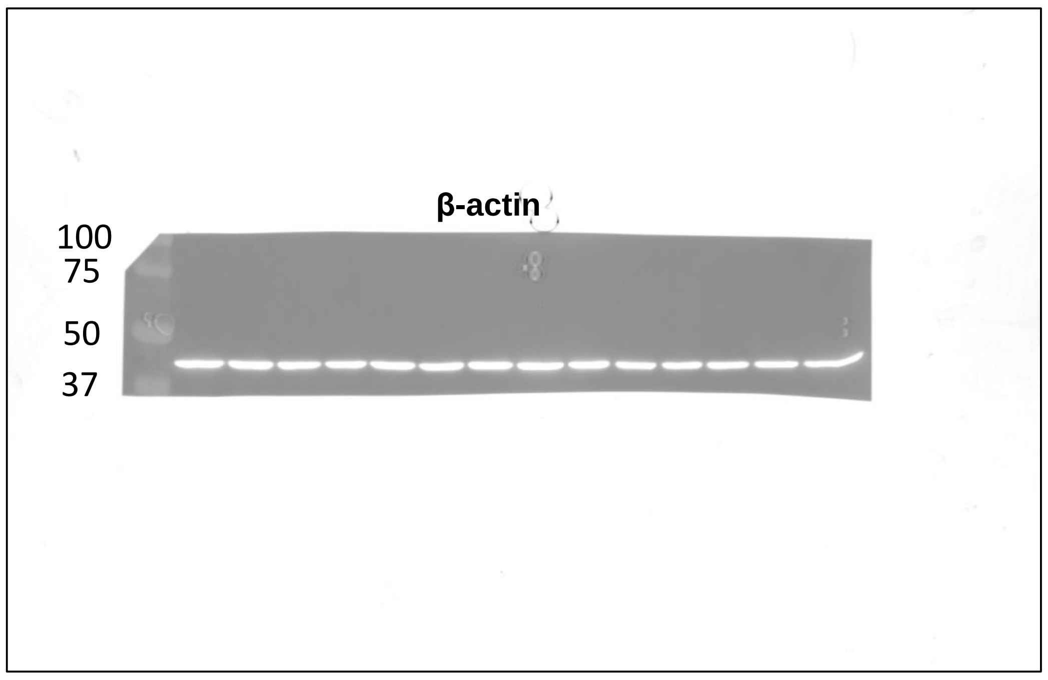

Raw Images of Western Blot for Supplementary Figure 3A **Huynh et al**

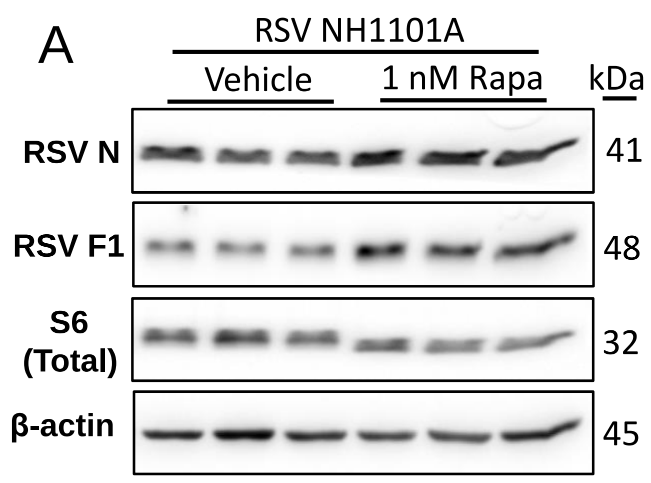

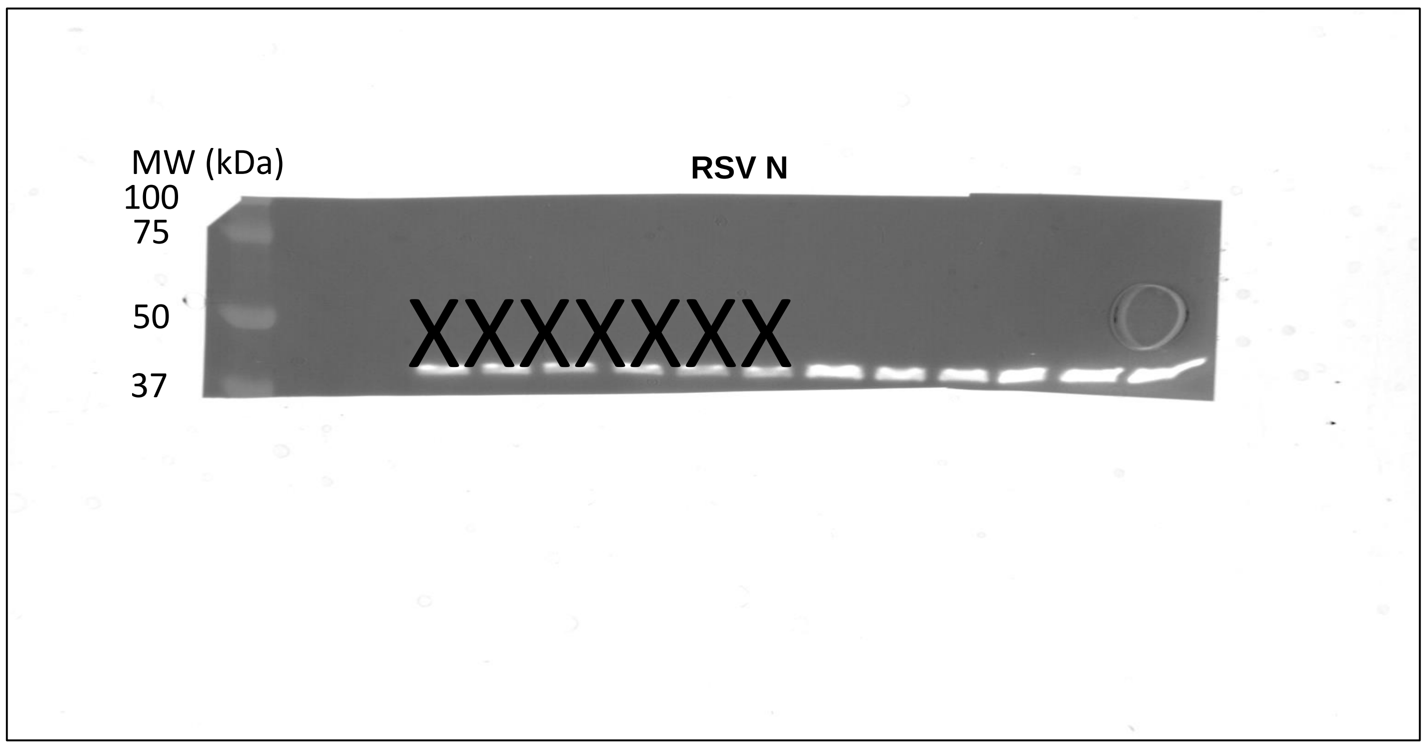

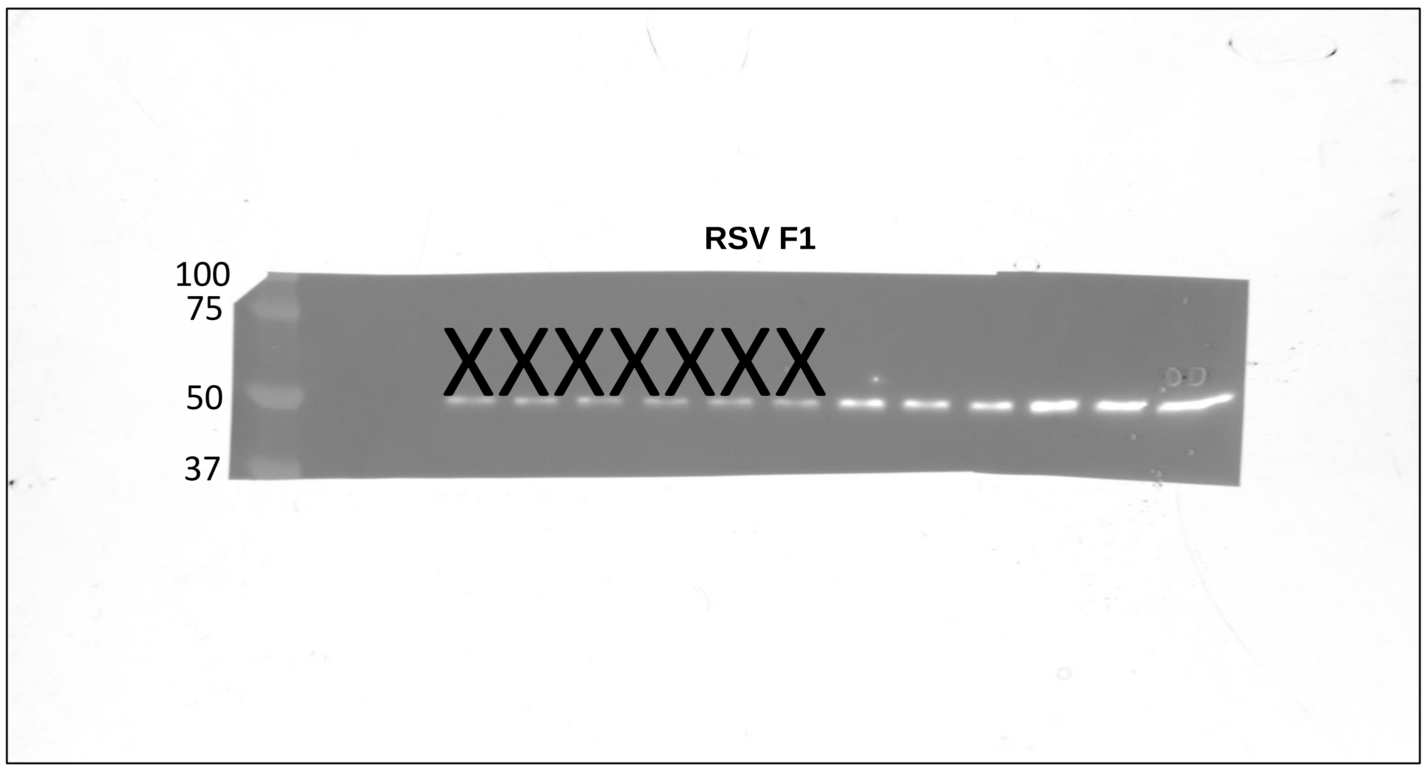

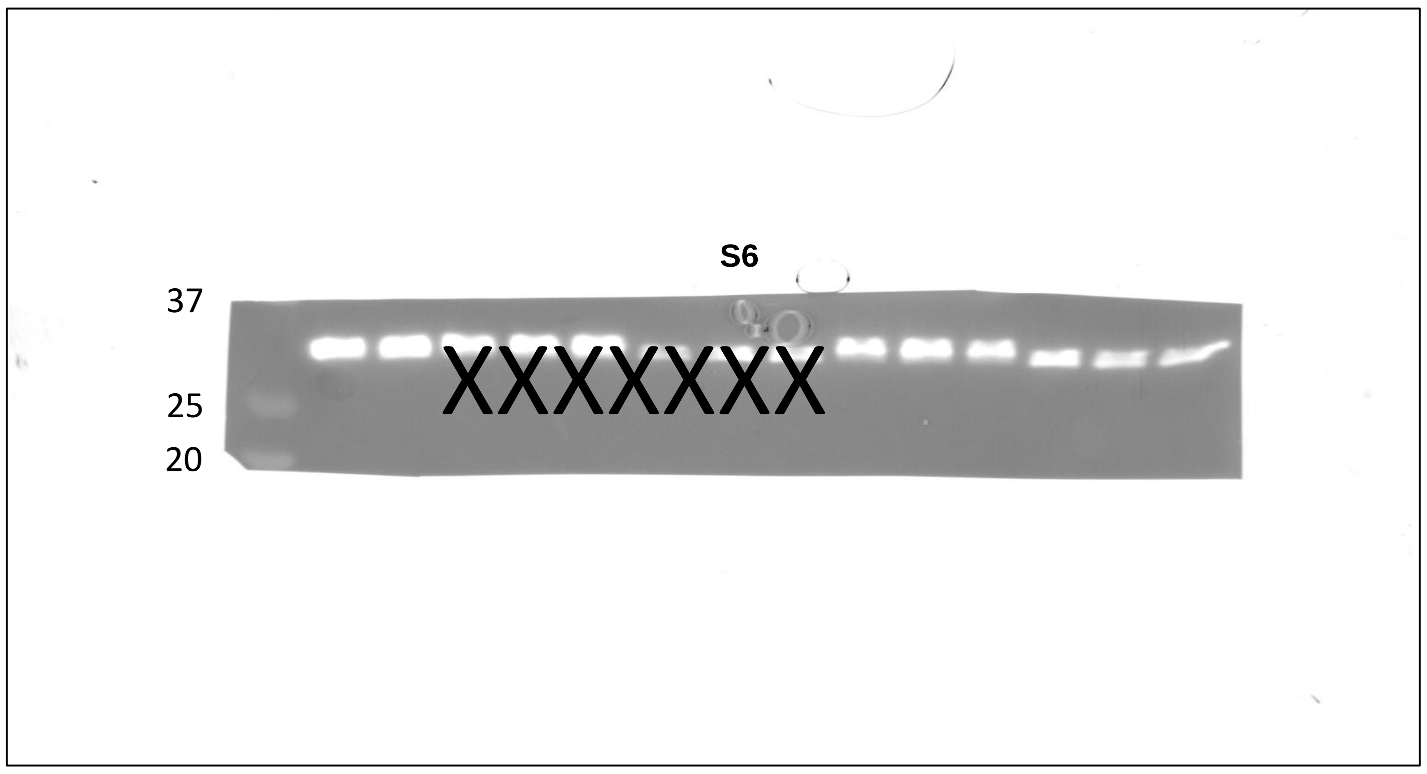

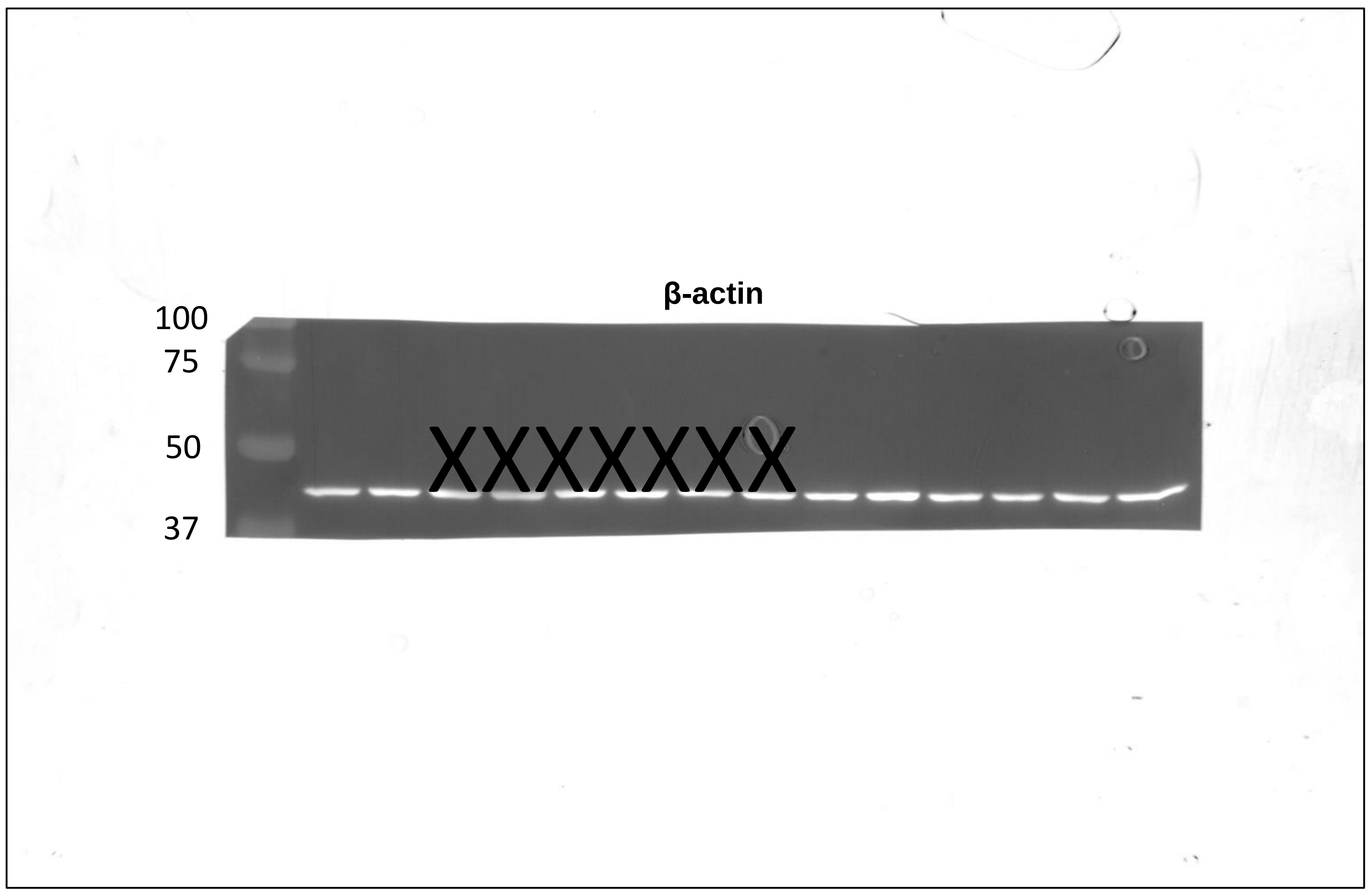

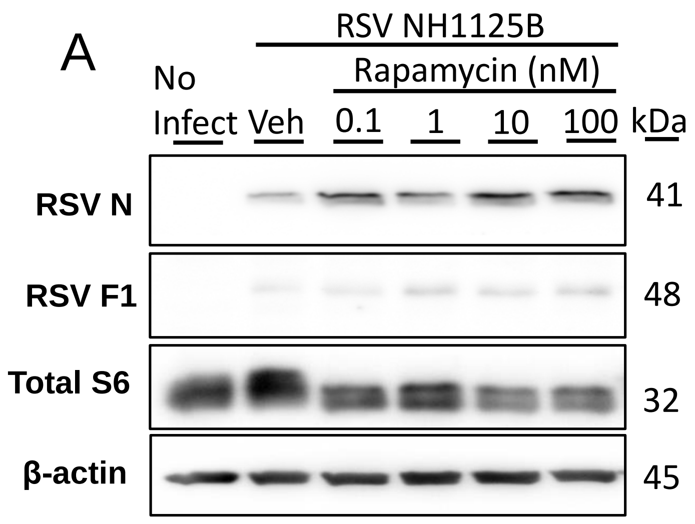
A
RSV NH1125B
Rapamycin (nM)
No Infect
Veh
0.1
1
10
100
kDa
RSV N
41
RSV F1
48
Total S6
32
β-actin
45

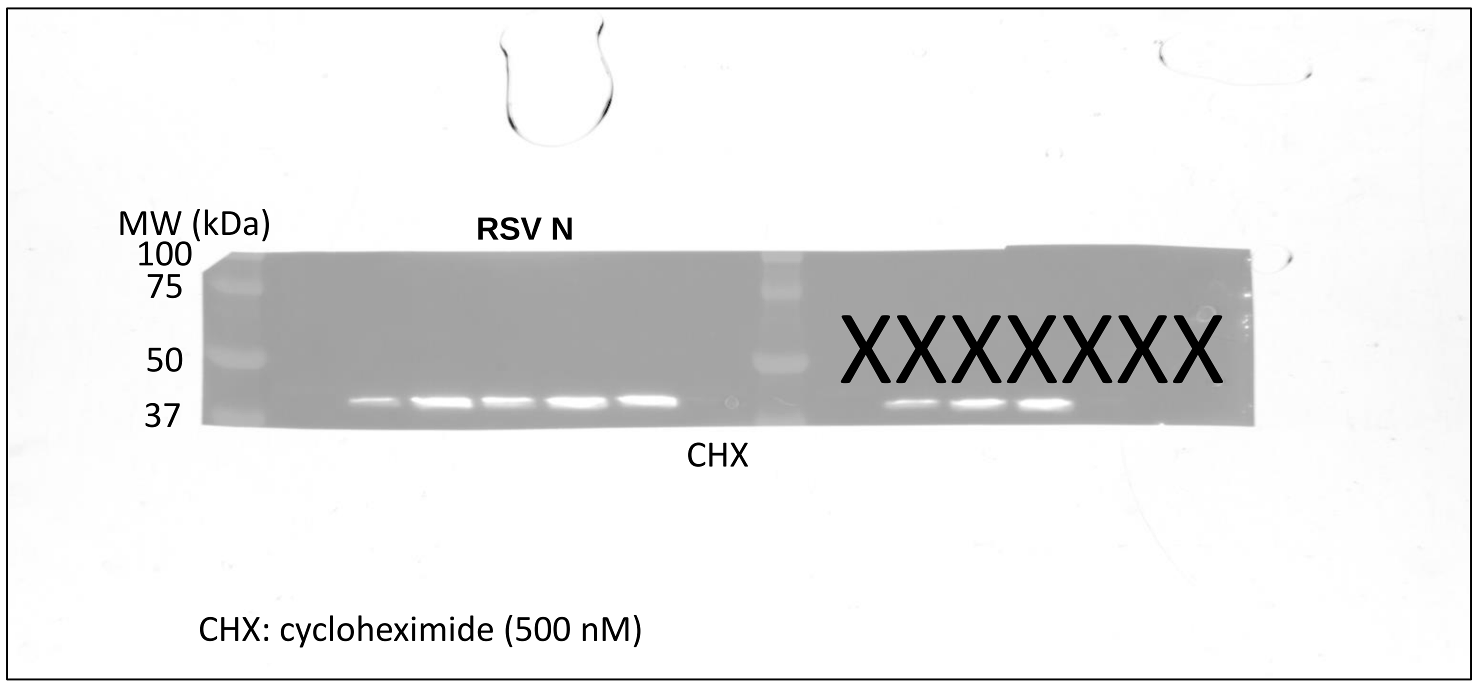
MW (kDa)
RSV N
100
75
50
37
XXXXXXX
CHX
CHX: cycloheximide (500 nM)

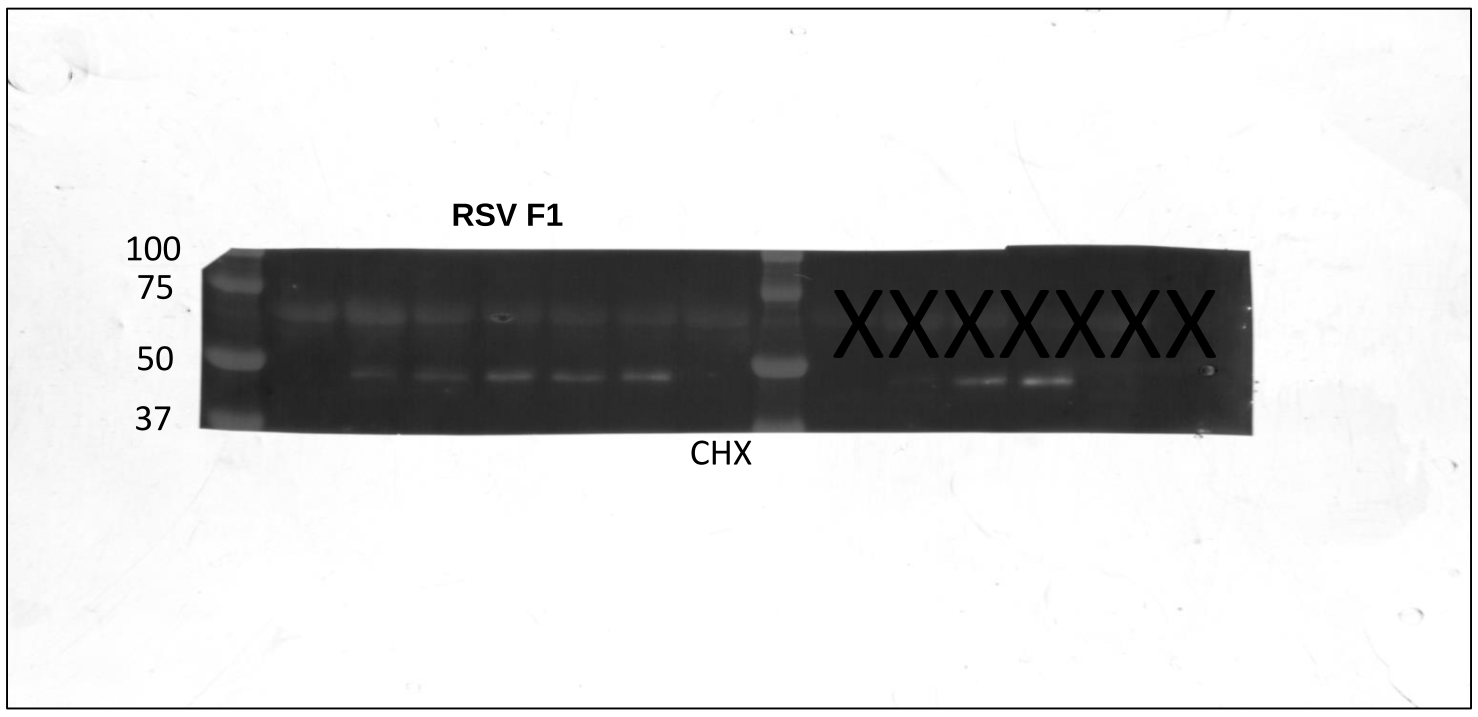
RSV F1
100
75
50
37
XXXXXXX
CHX

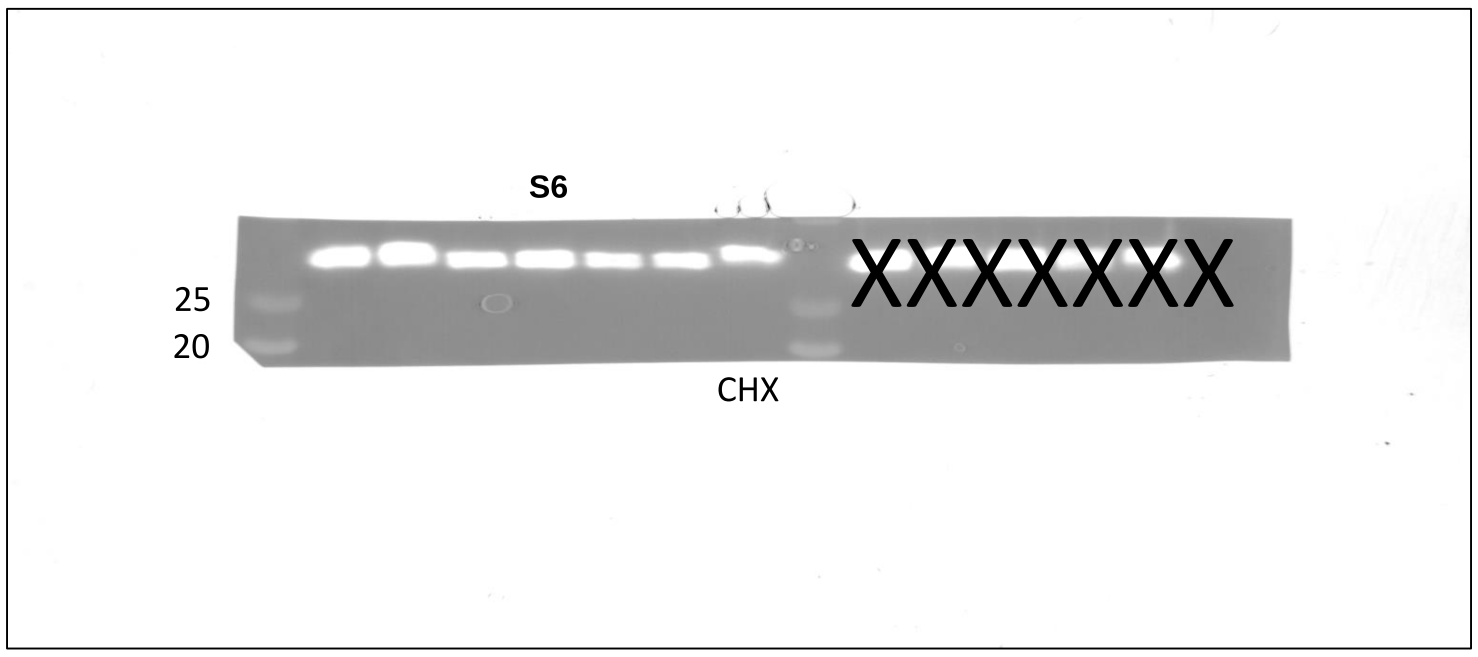
S6
25
20
XXXXXXX
CHX

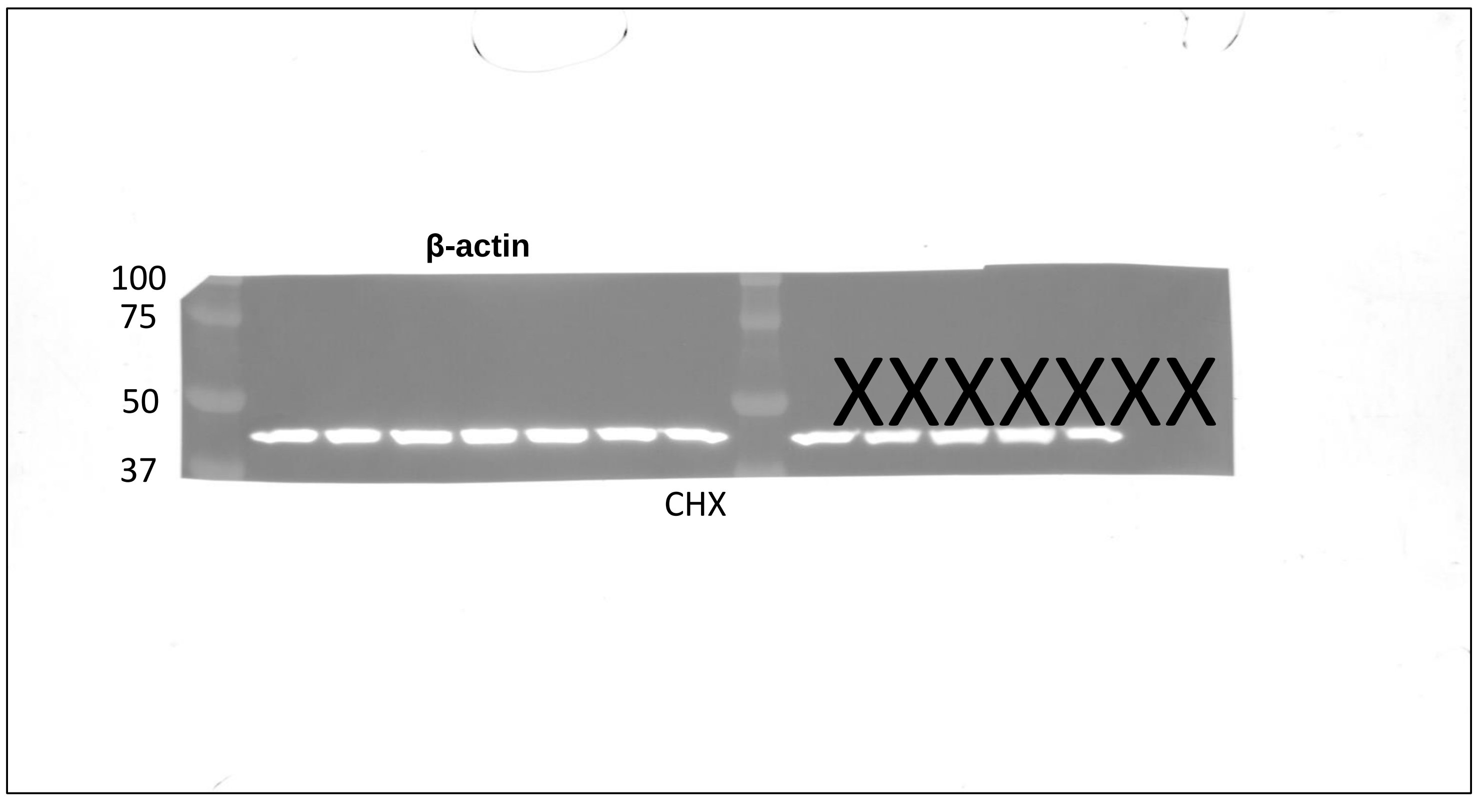
β-actin
100
75
50
37
XXXXXXX
CHX

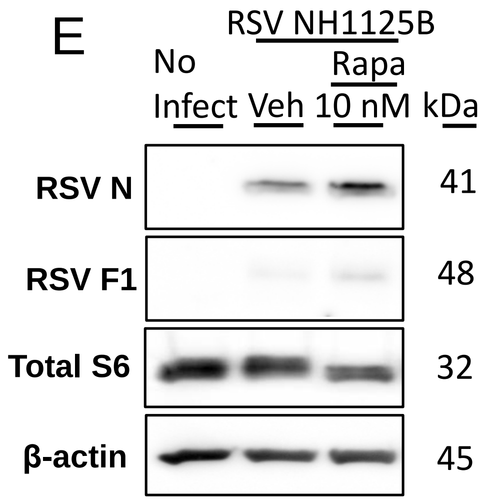
E
RSV NH1125B
No Infect
Veh
Rapa 10 nM
kDa
RSV N
41
RSV F1
48
Total S6
32
β-actin
45

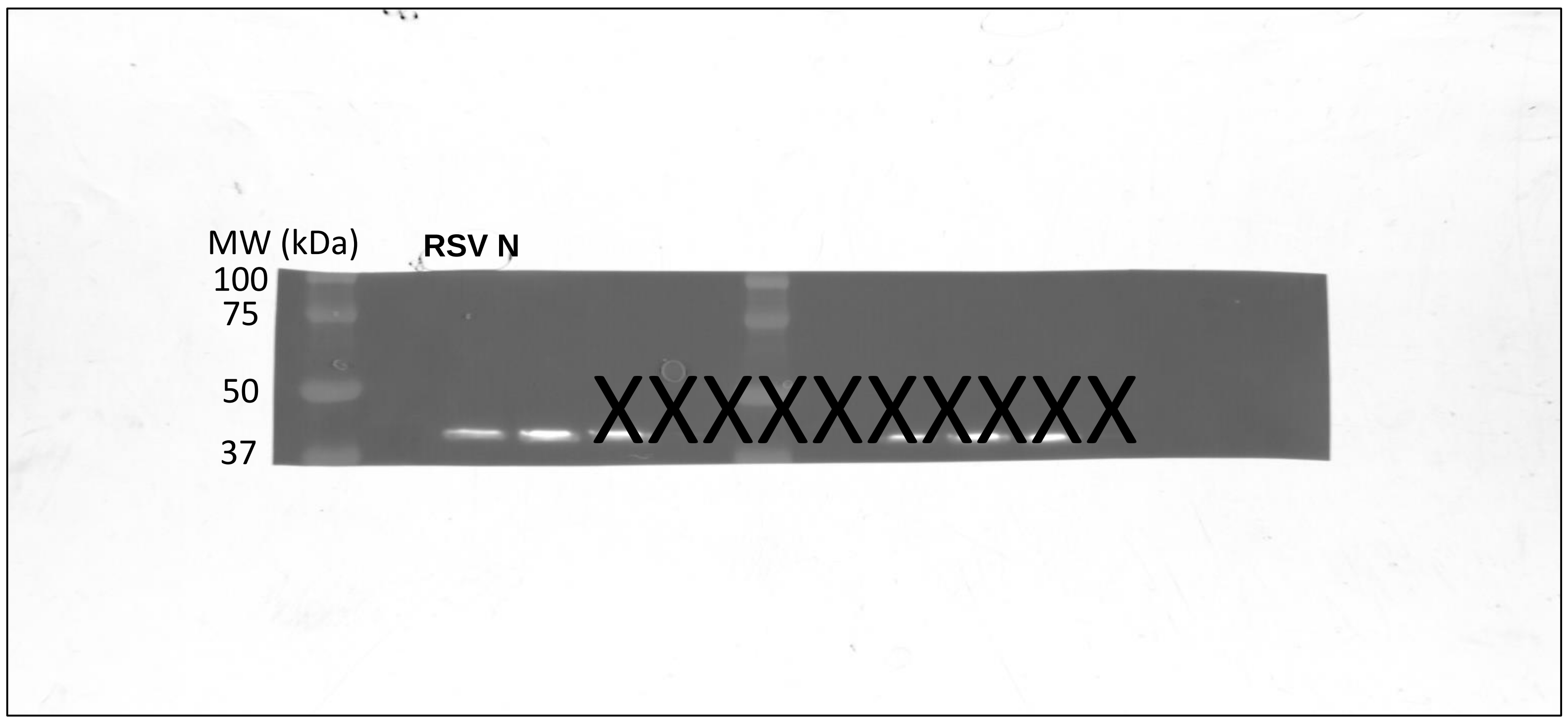
MW (kDa)
RSV N
100
75
50
37
XXXXXXXXXX

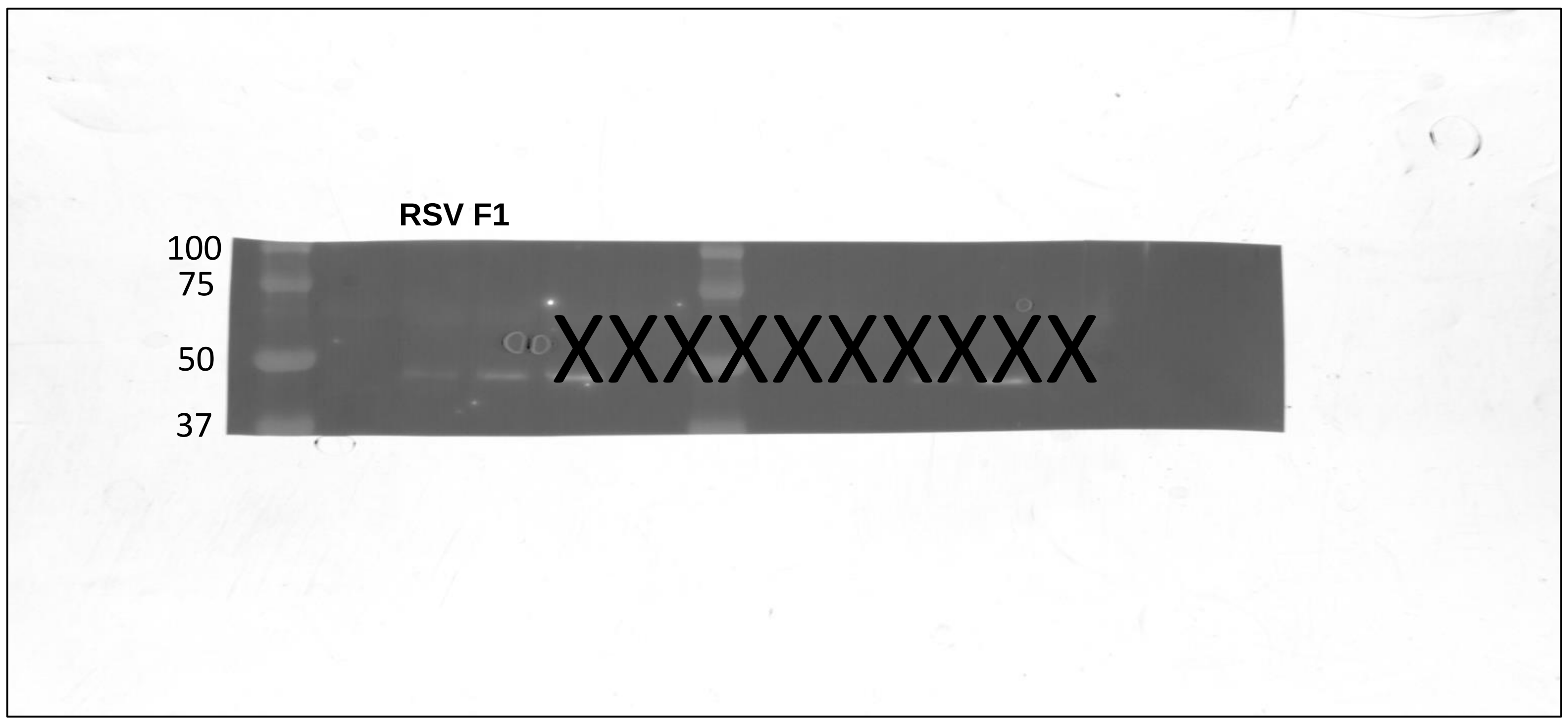
RSV F1
100
75
50
37
XXXXXXXXXX

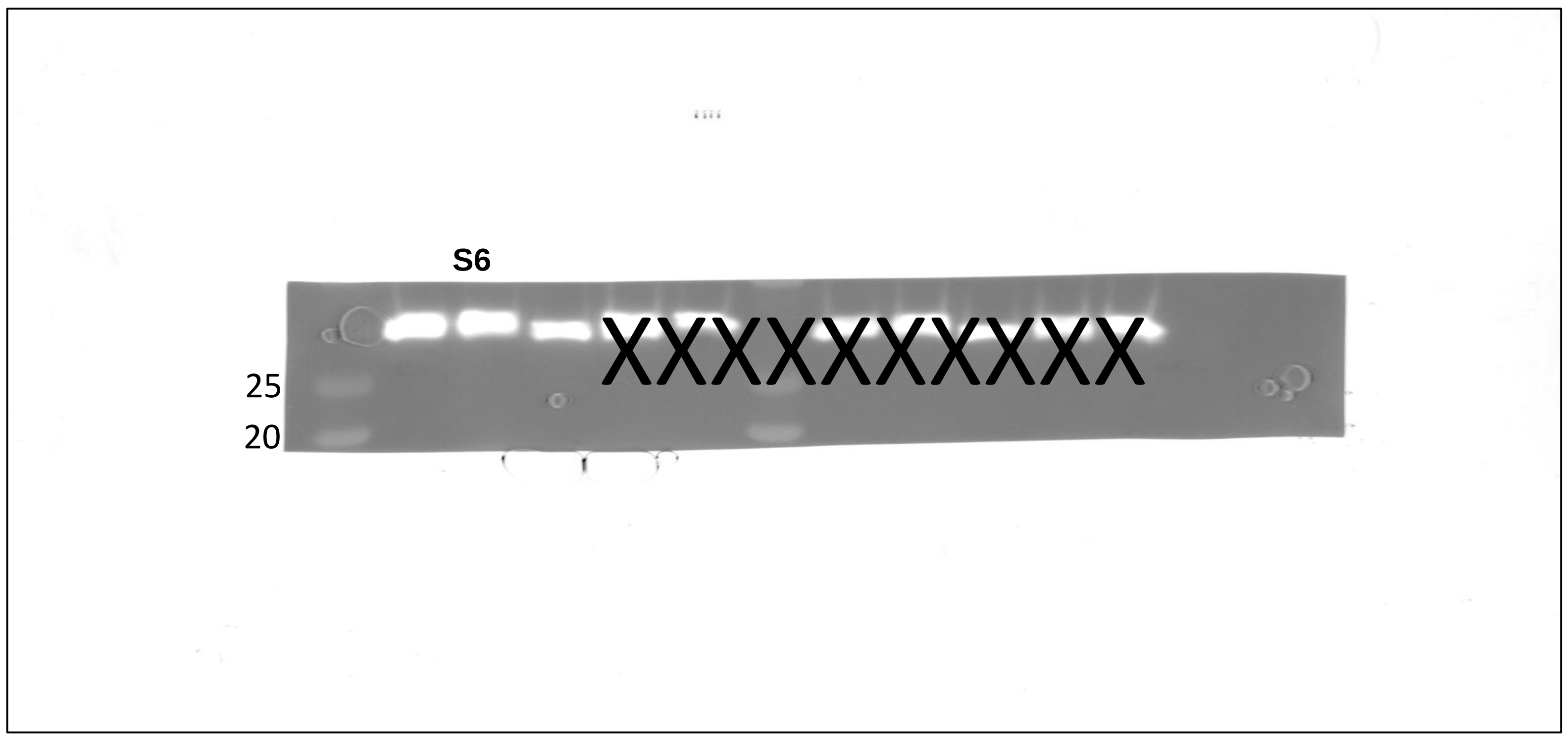
S6
25
20
XXXXXXXXXX

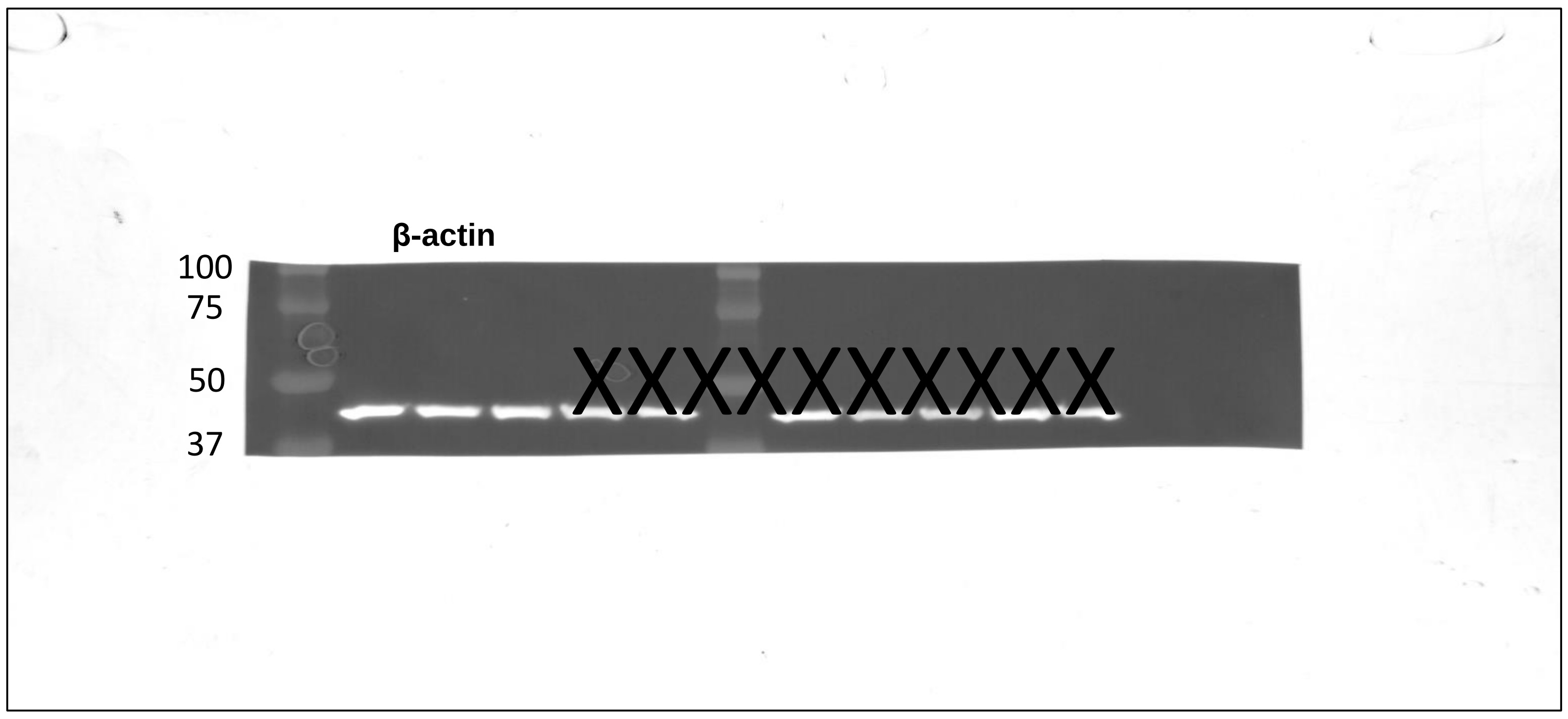
β-actin
100
75
50
37
XXXXXXXXXX

Raw Images of Western Blot for Supplementary Figure 5A

Huynh et al

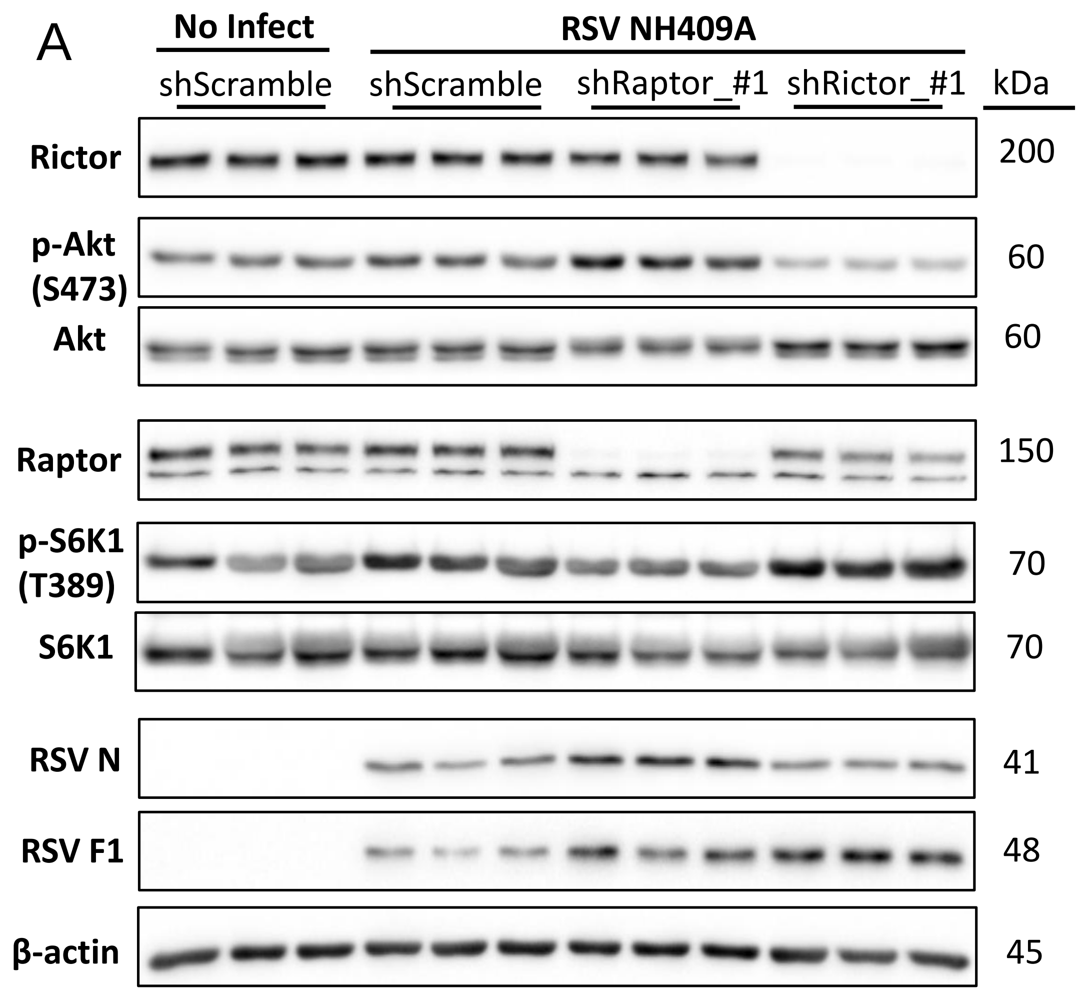

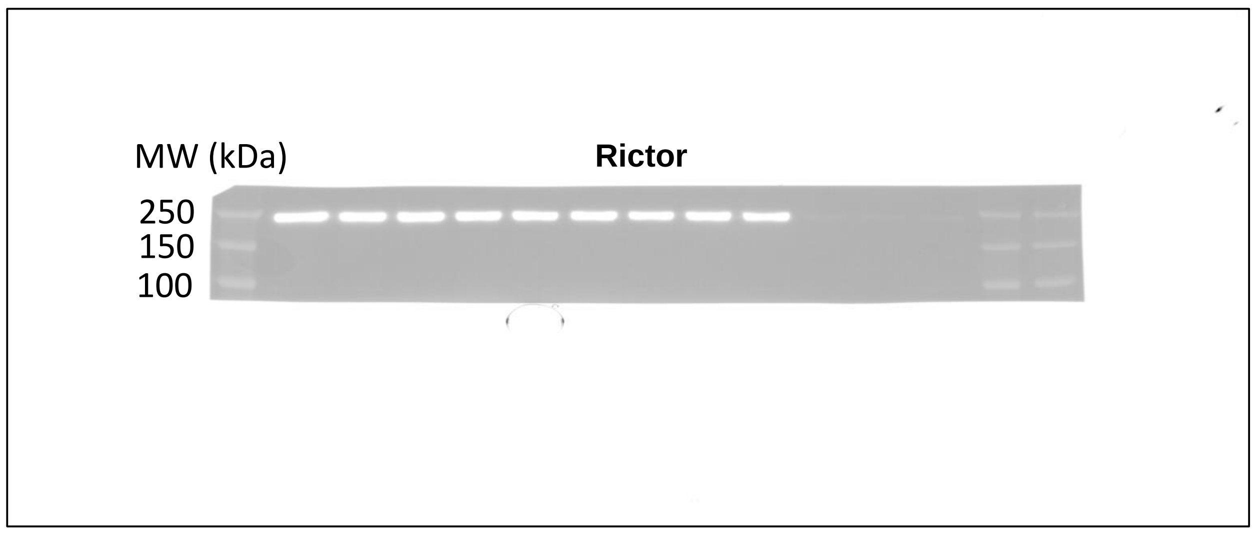

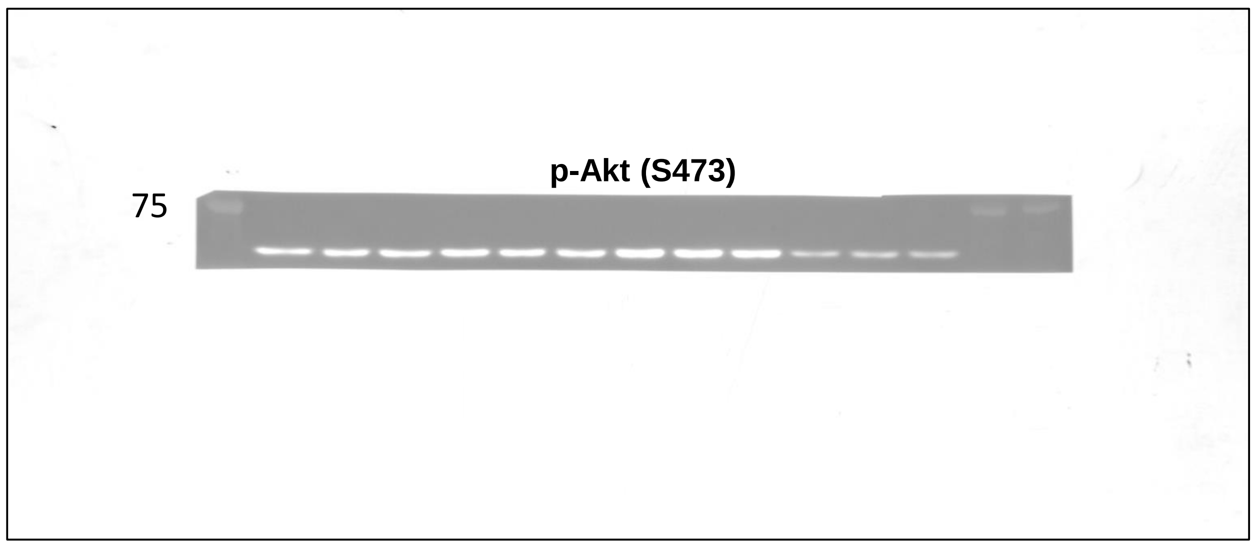

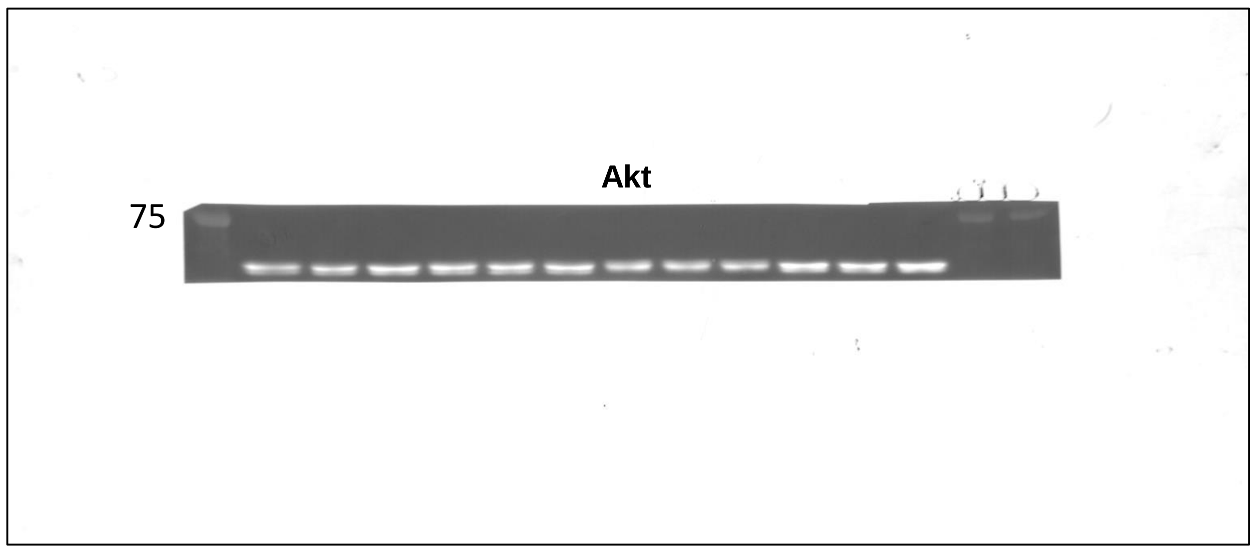

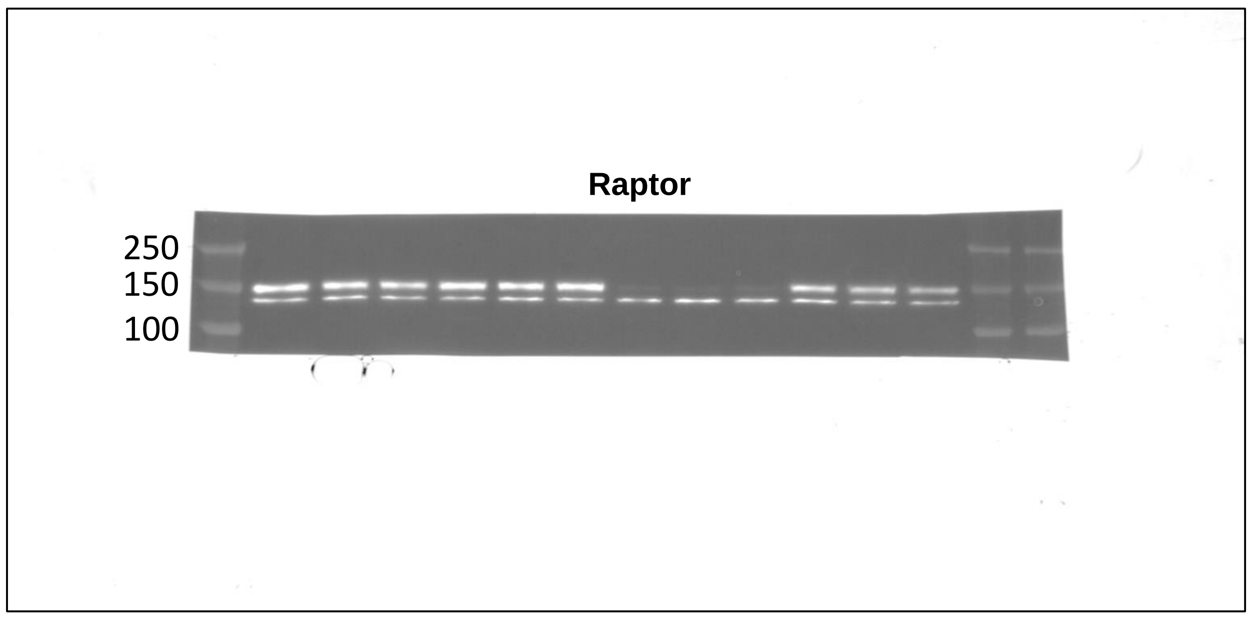

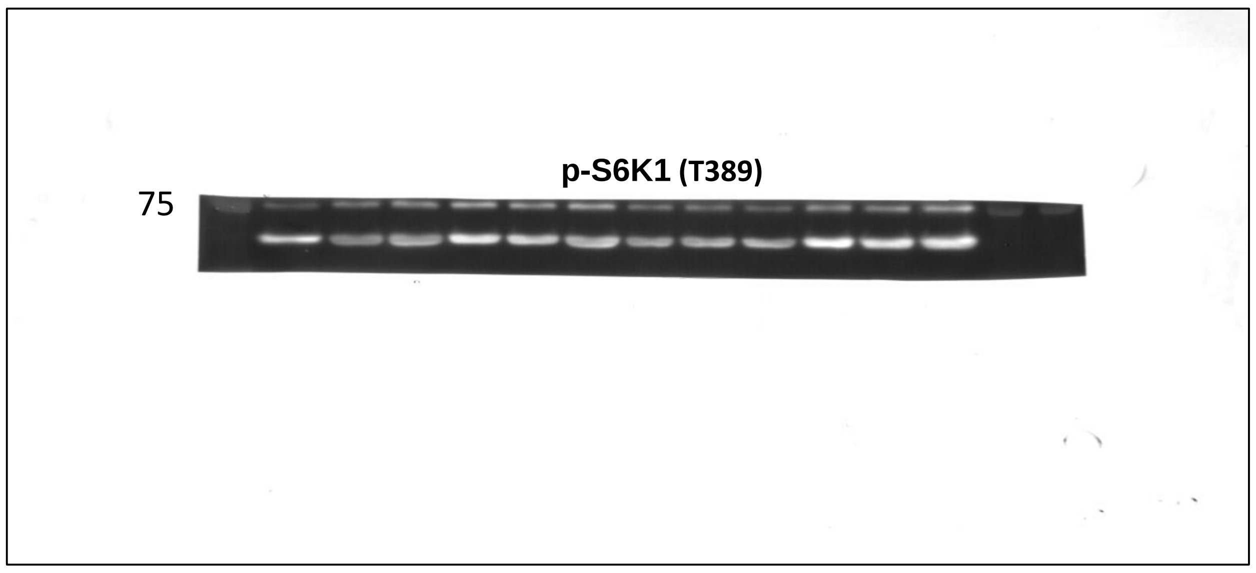

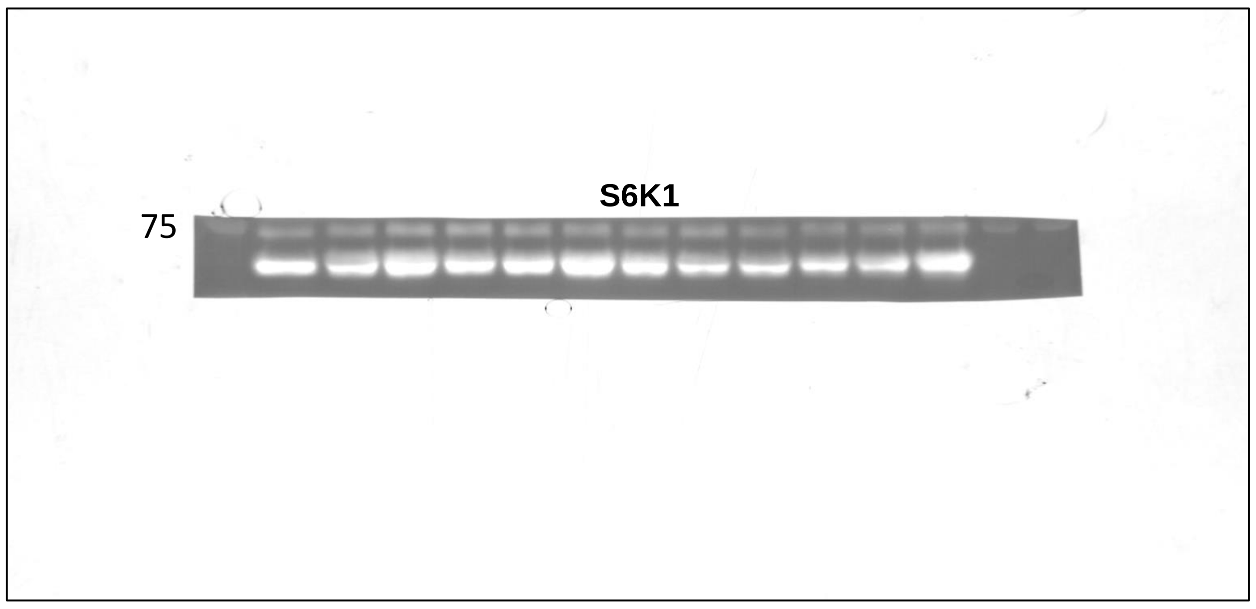

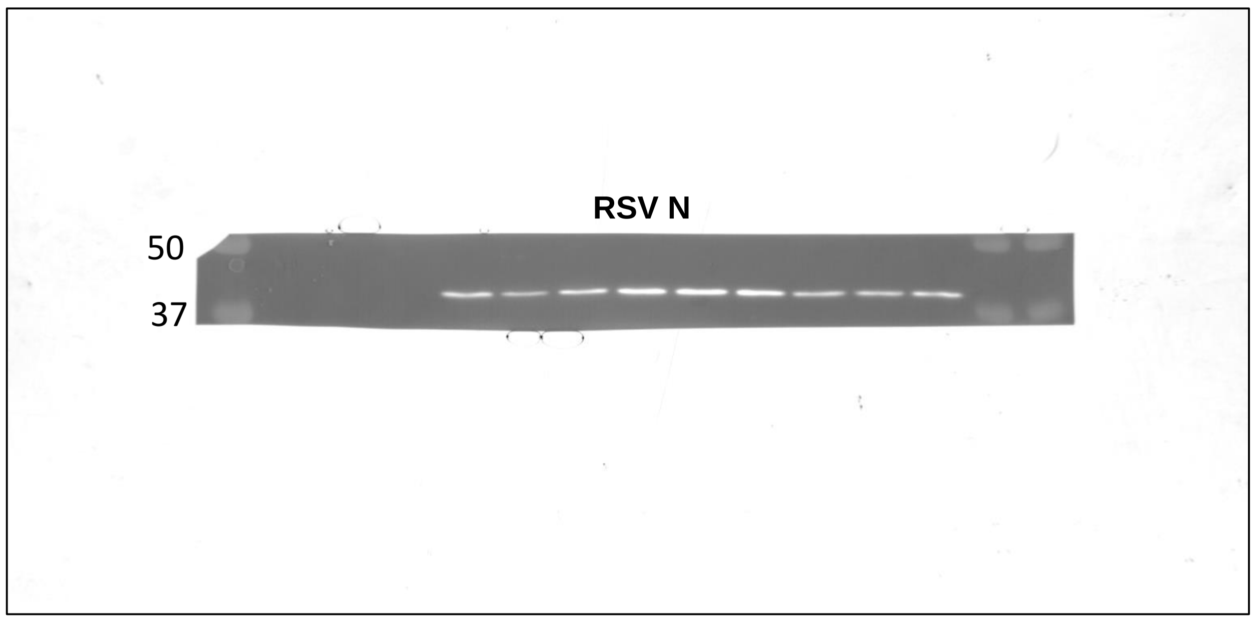

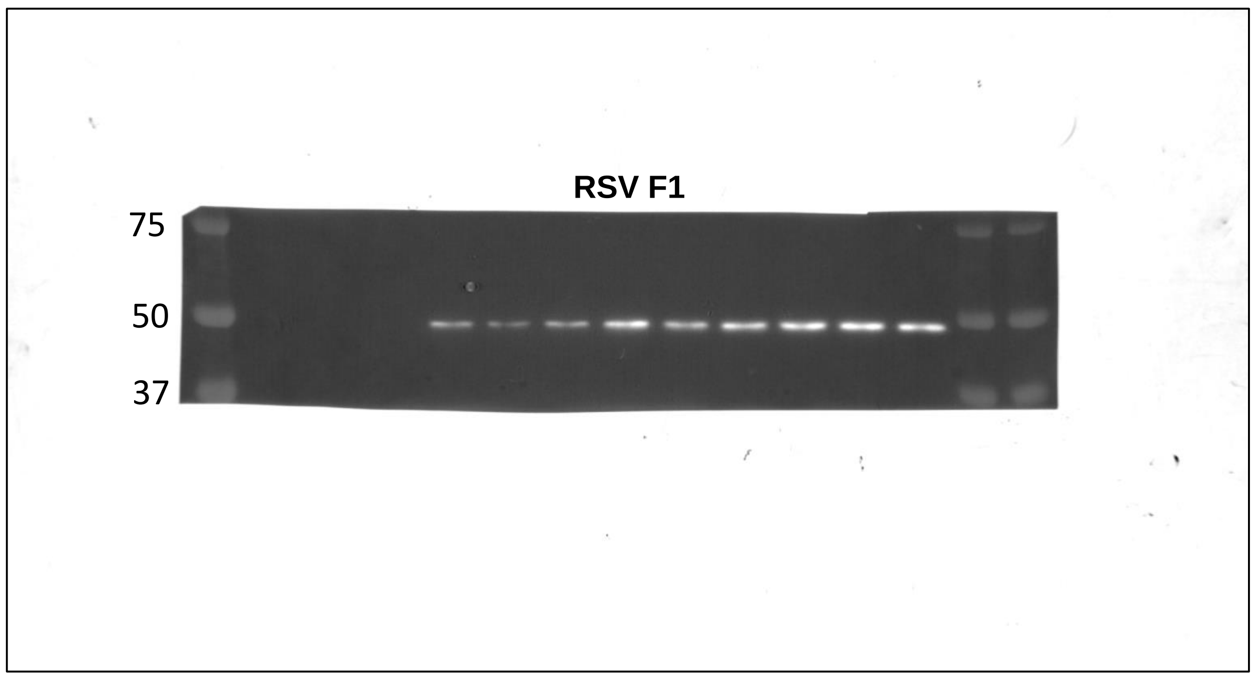

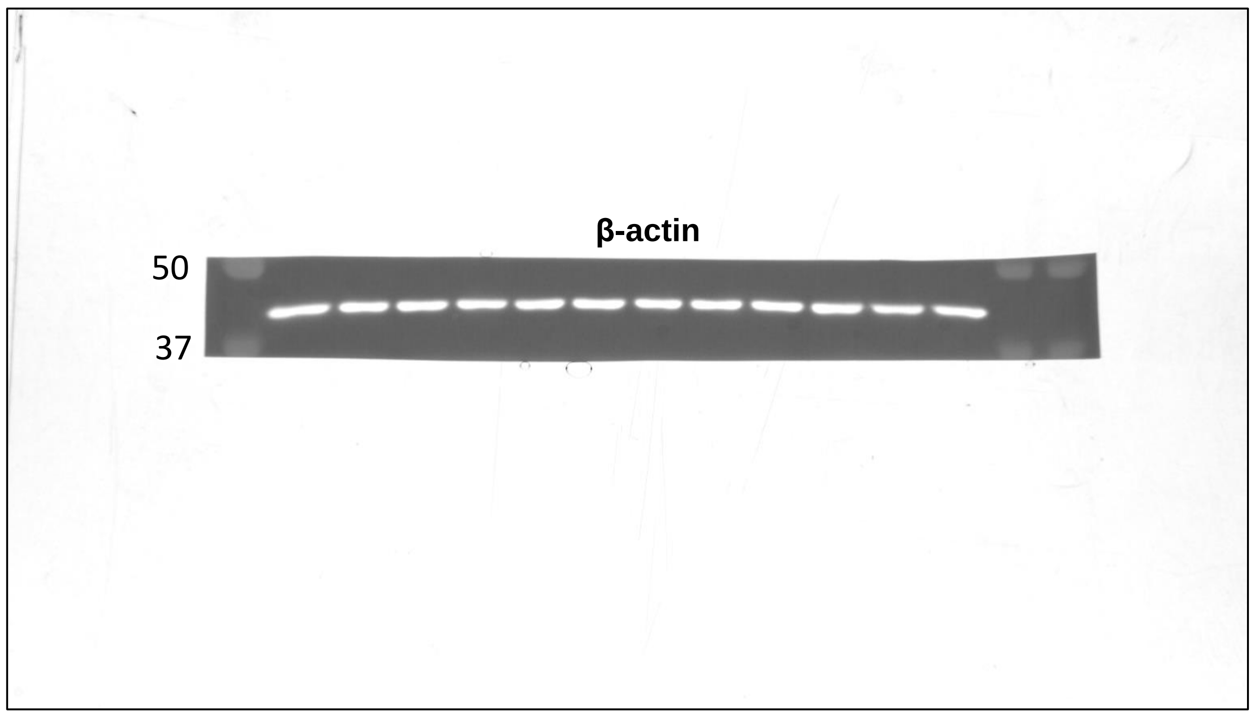

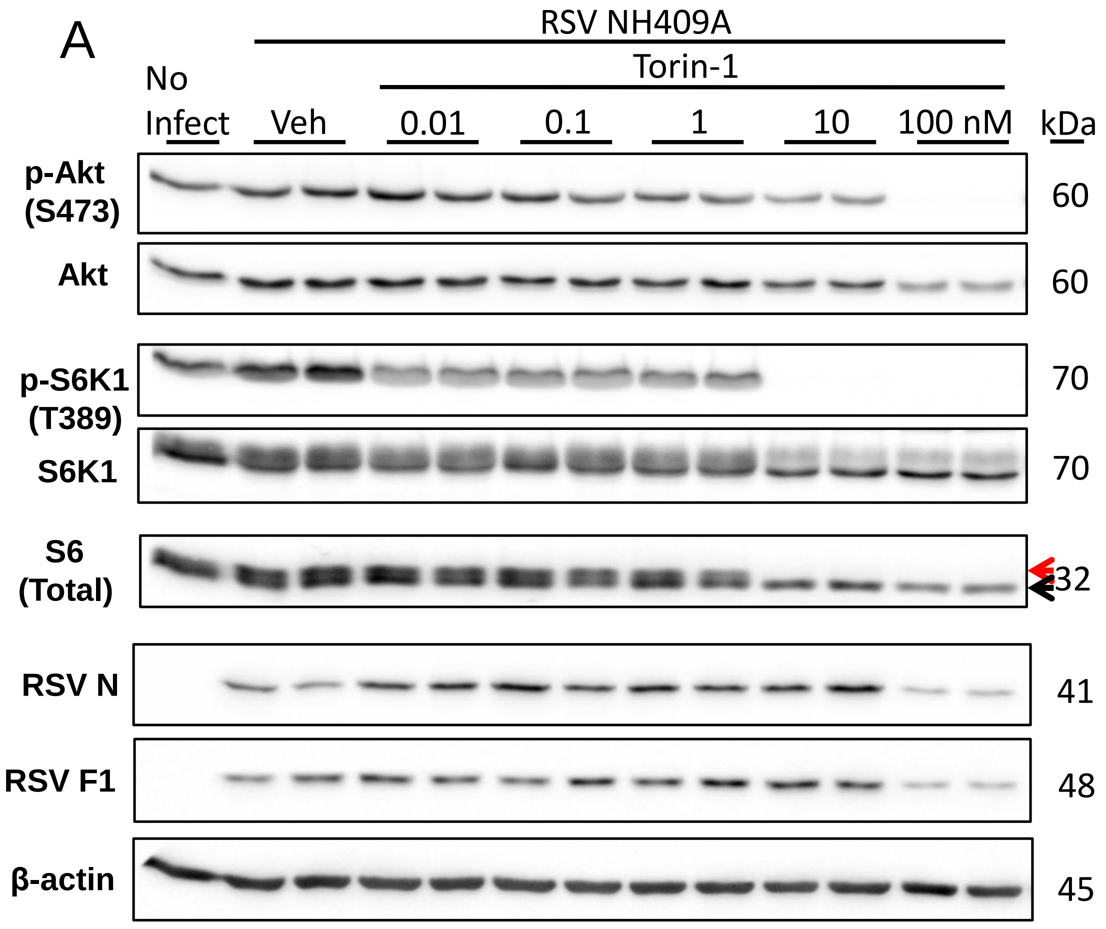

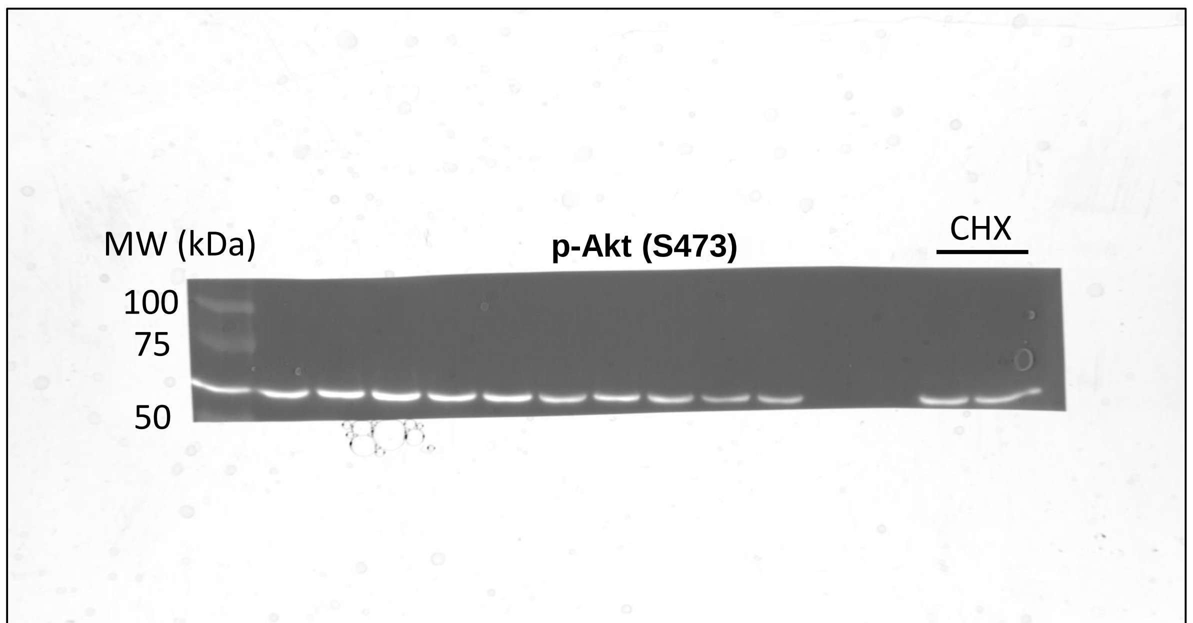

CHX: cycloheximide (500 nM as positive control)

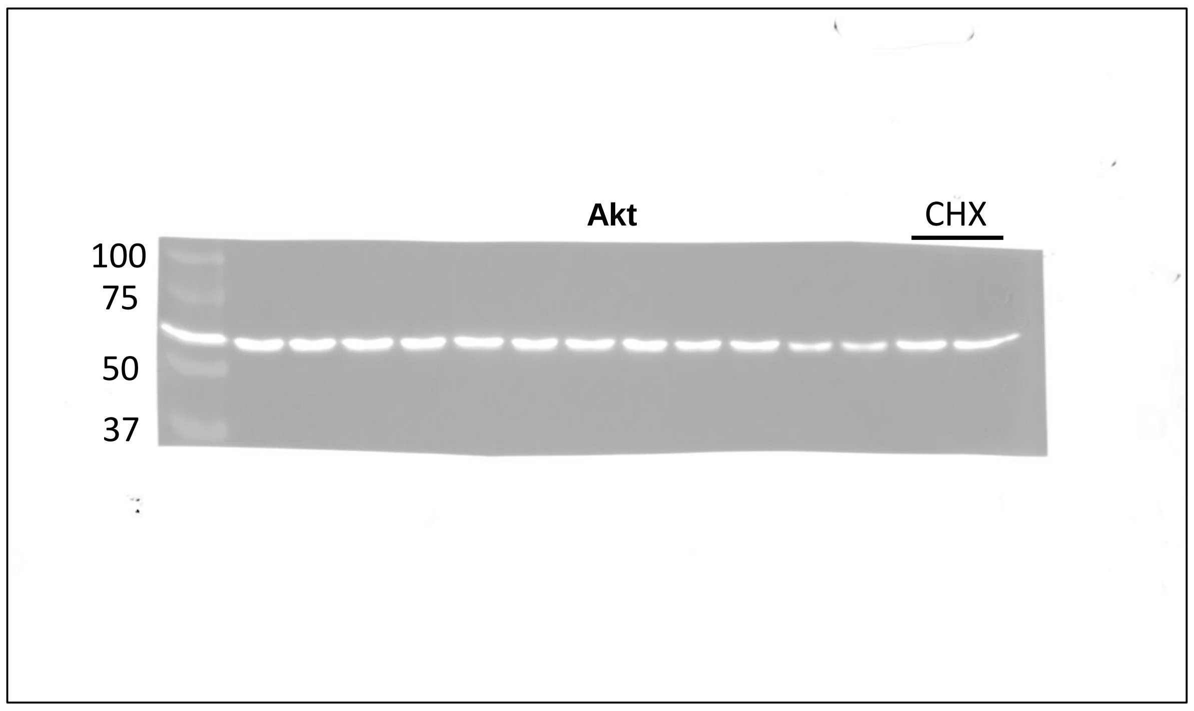

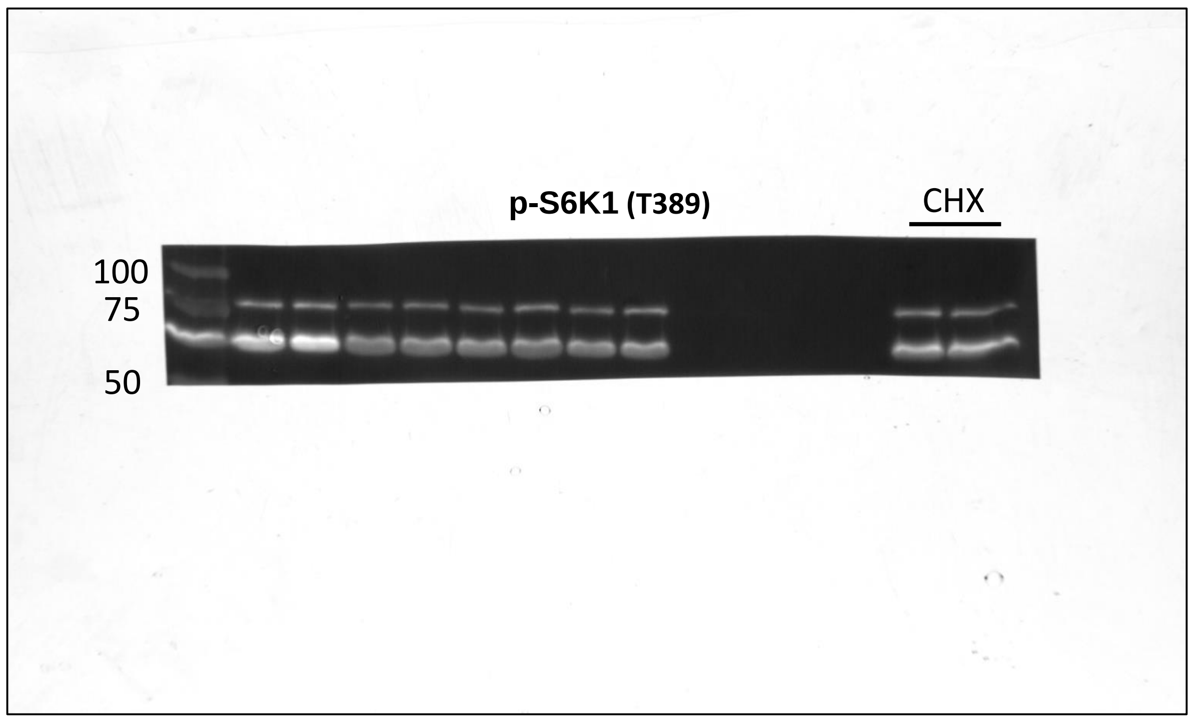

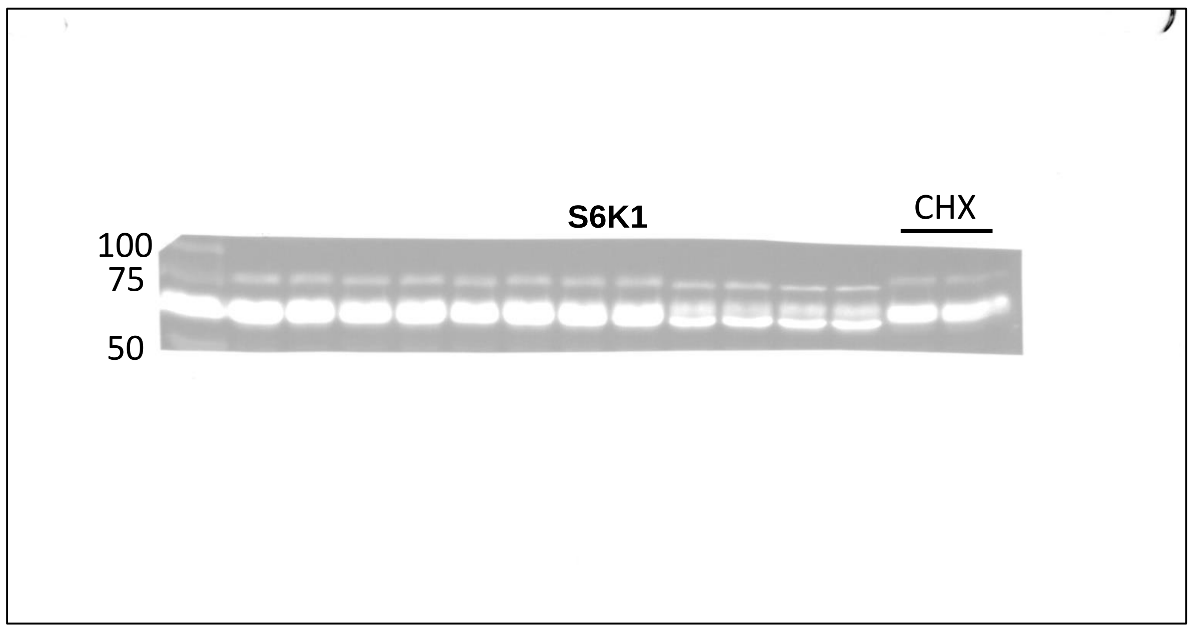

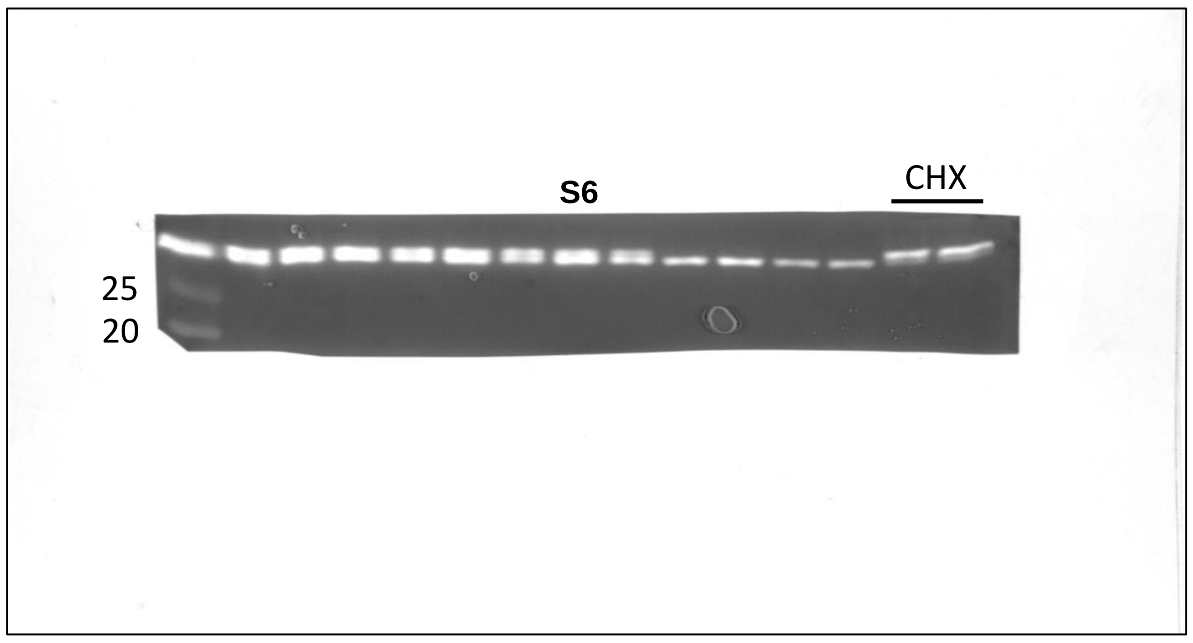

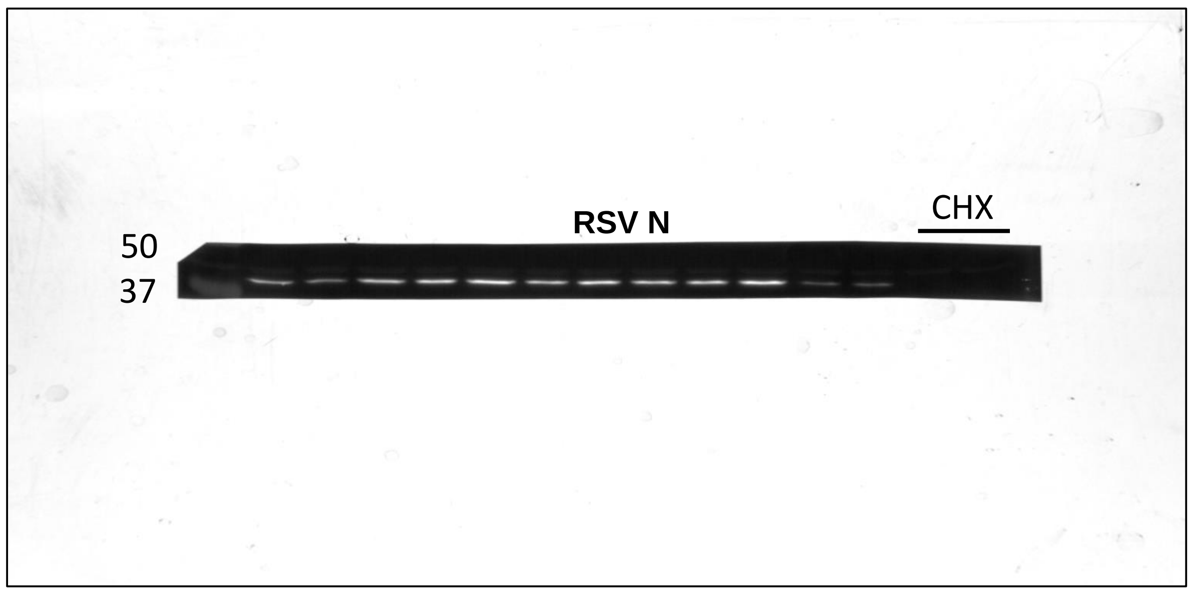

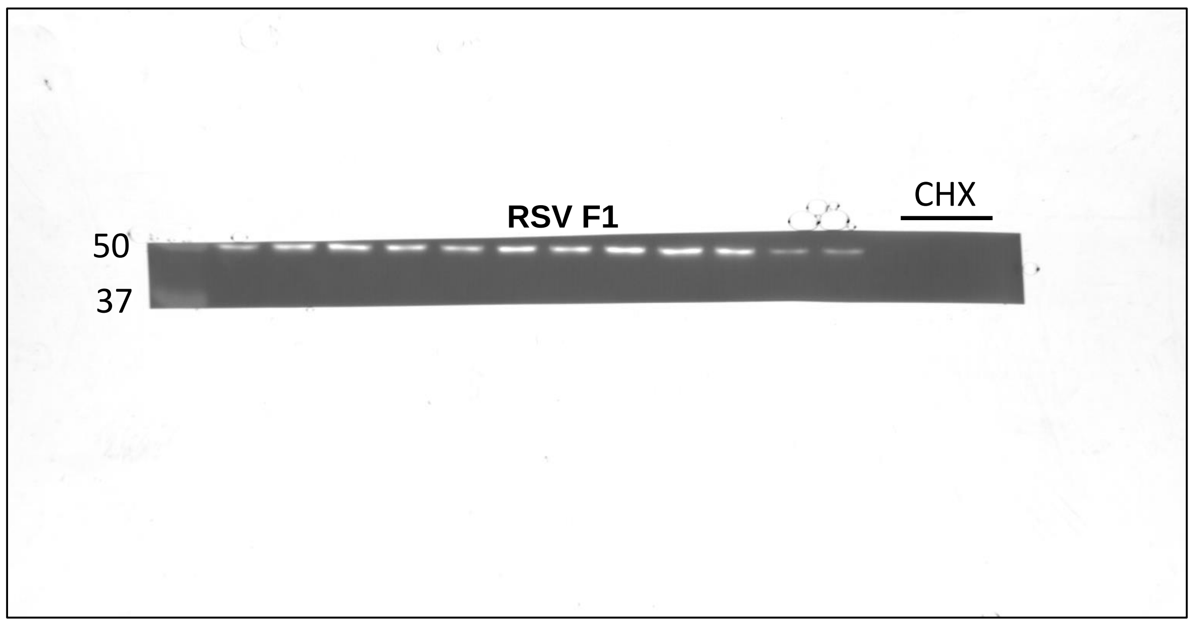

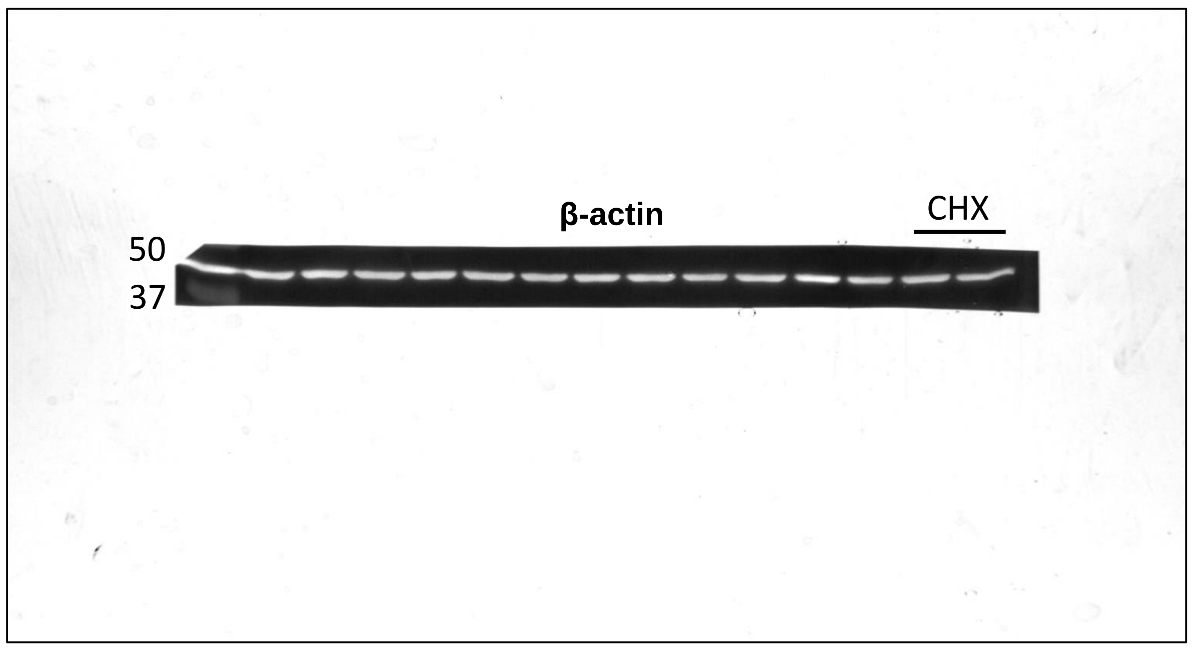

Raw Images of Western Blot for Supplementary Figure 8A **Huynh et al**

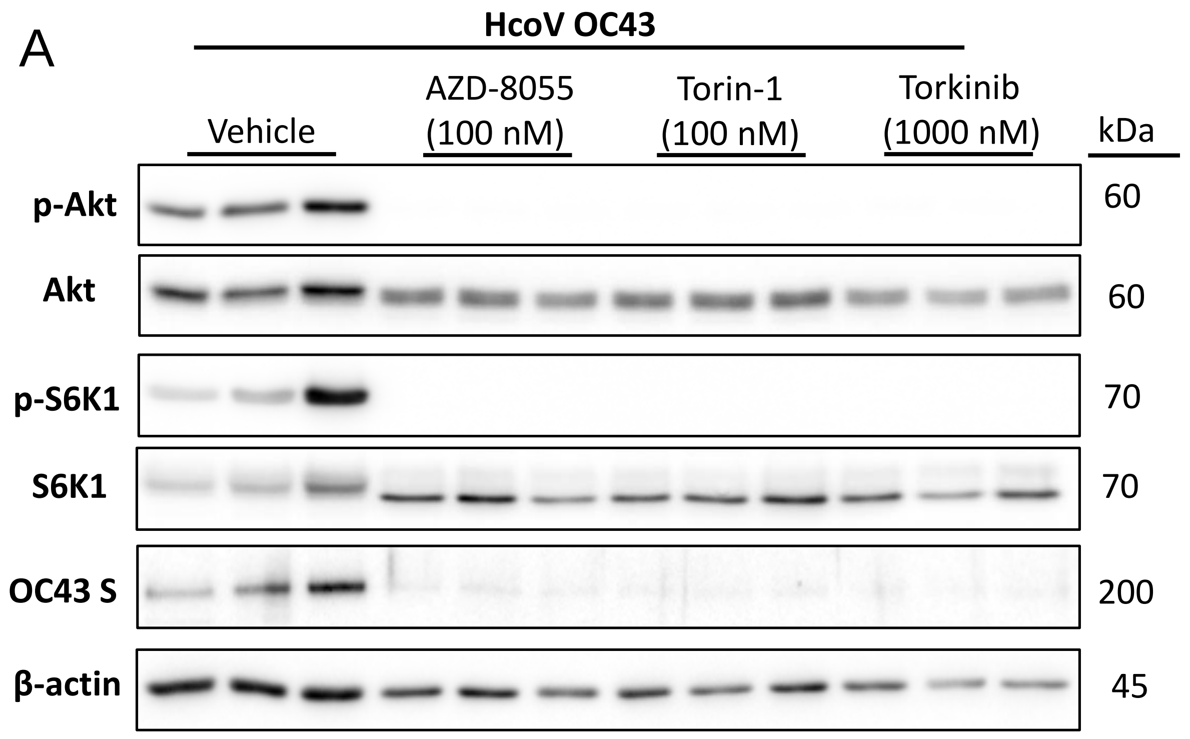

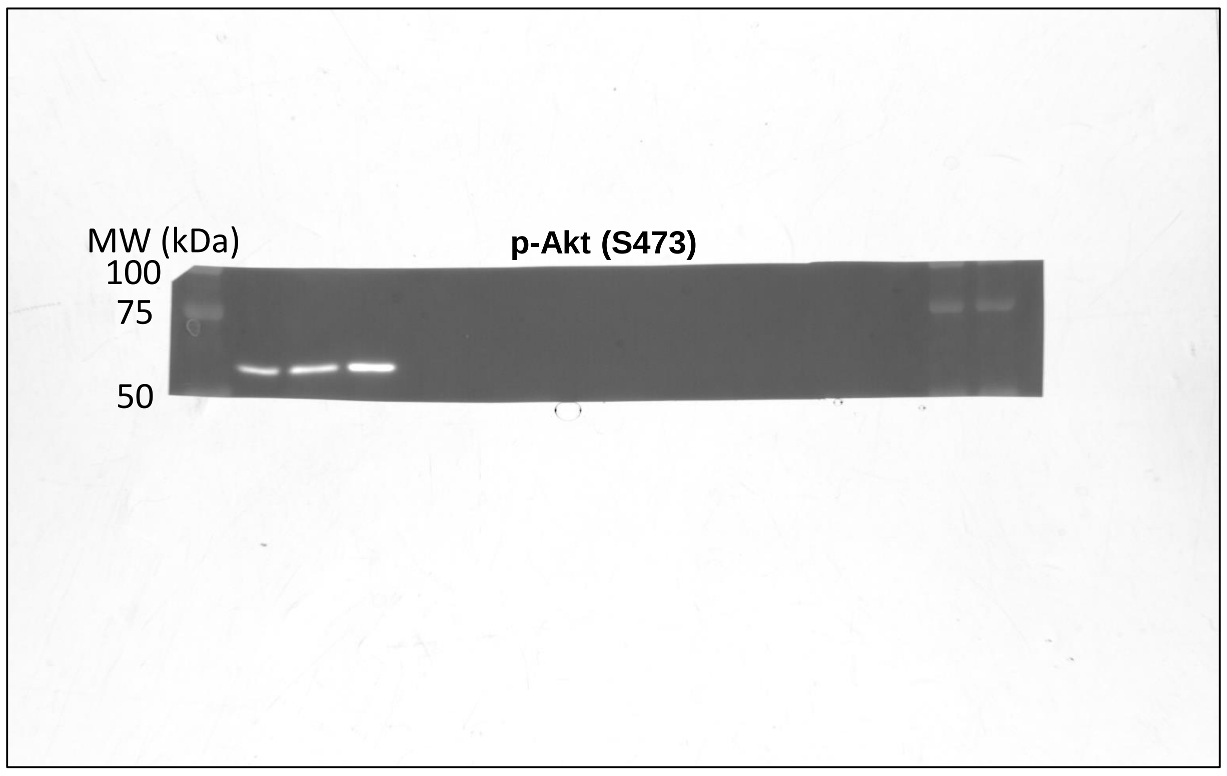

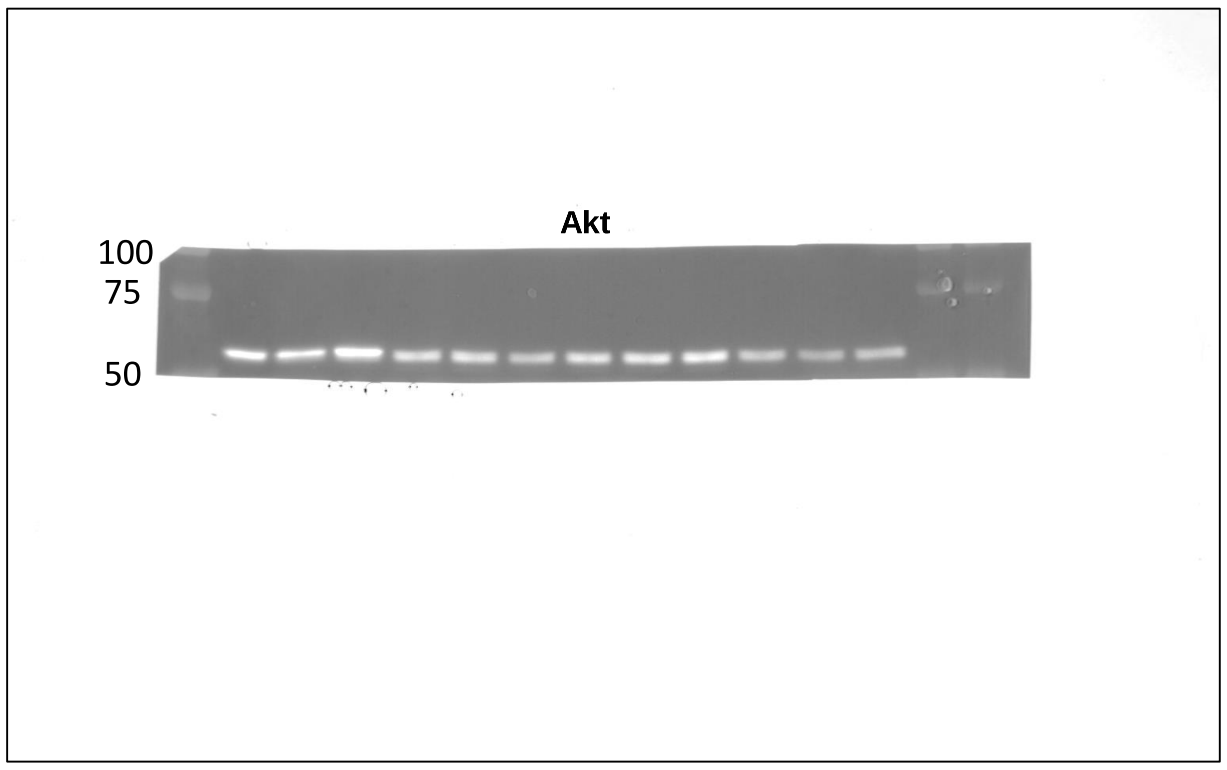

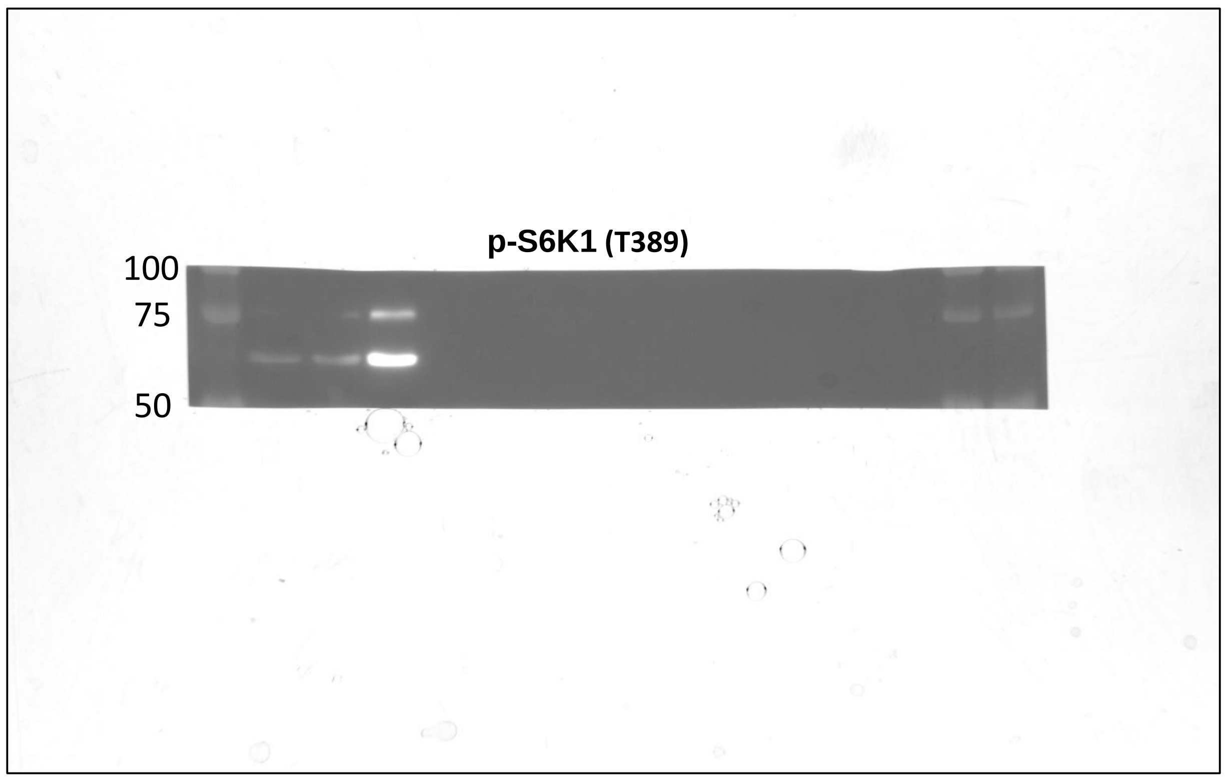

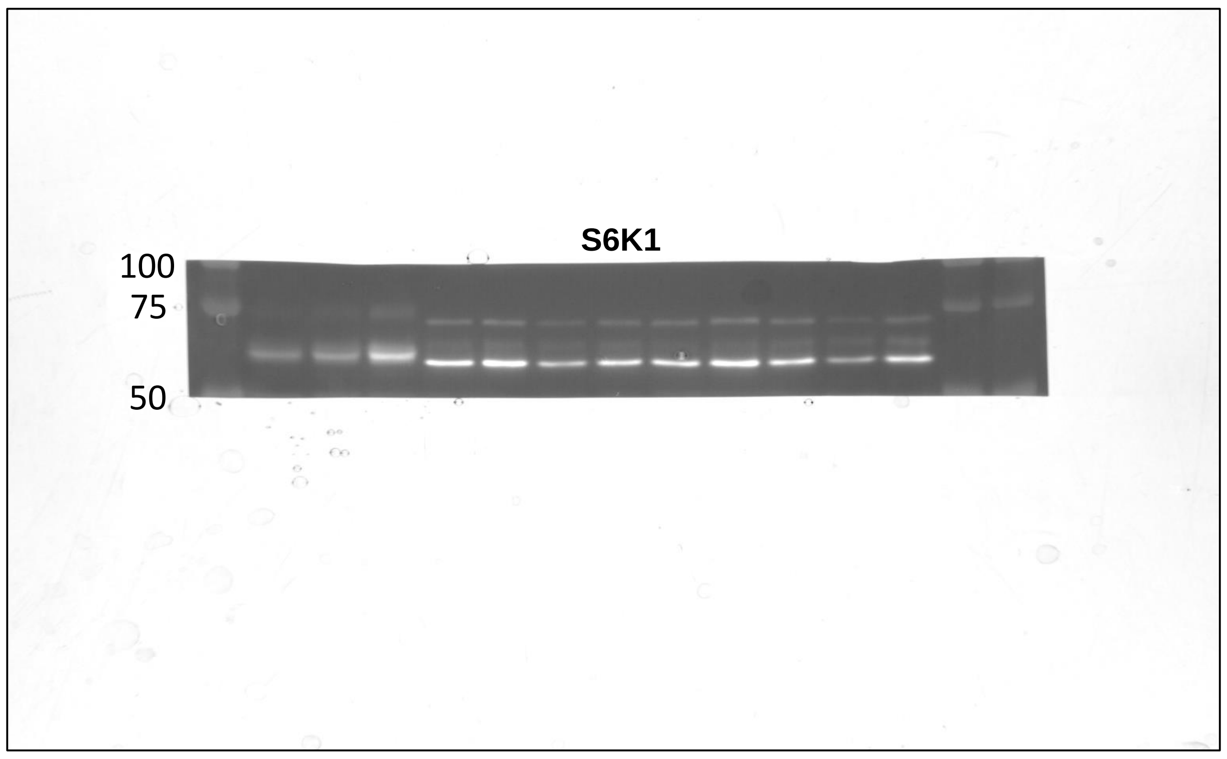

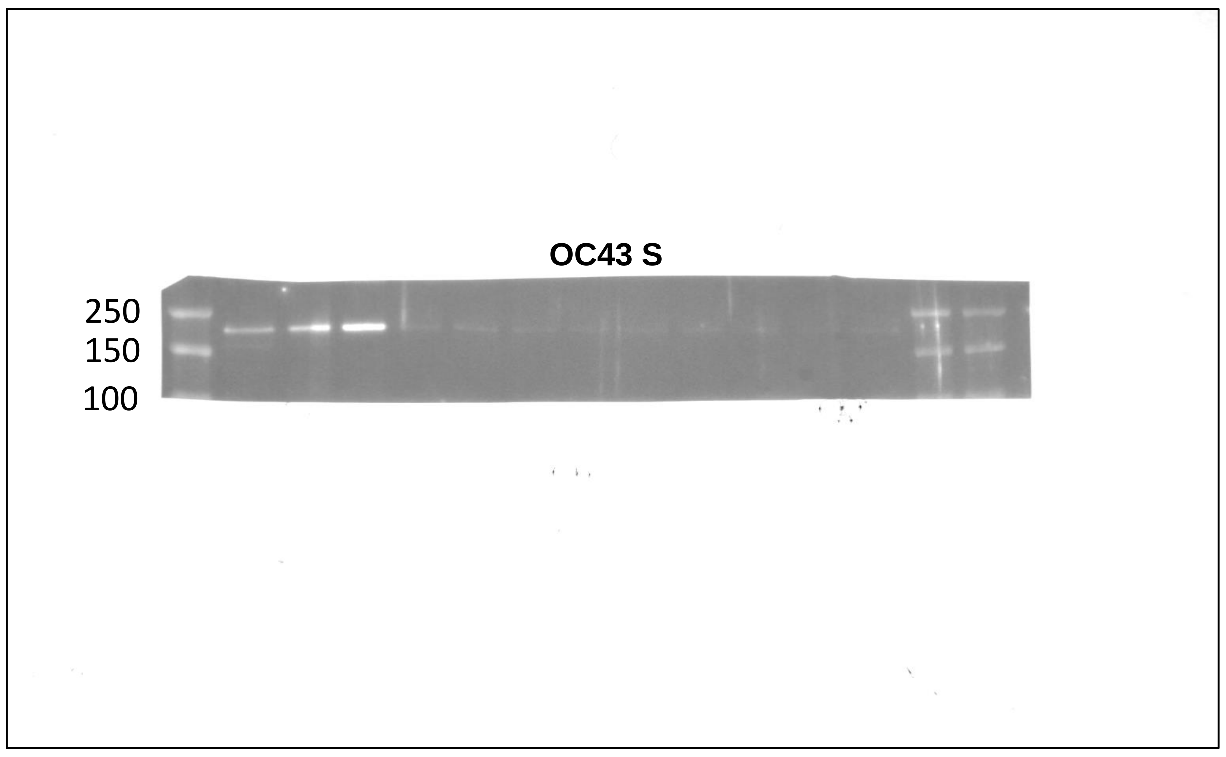

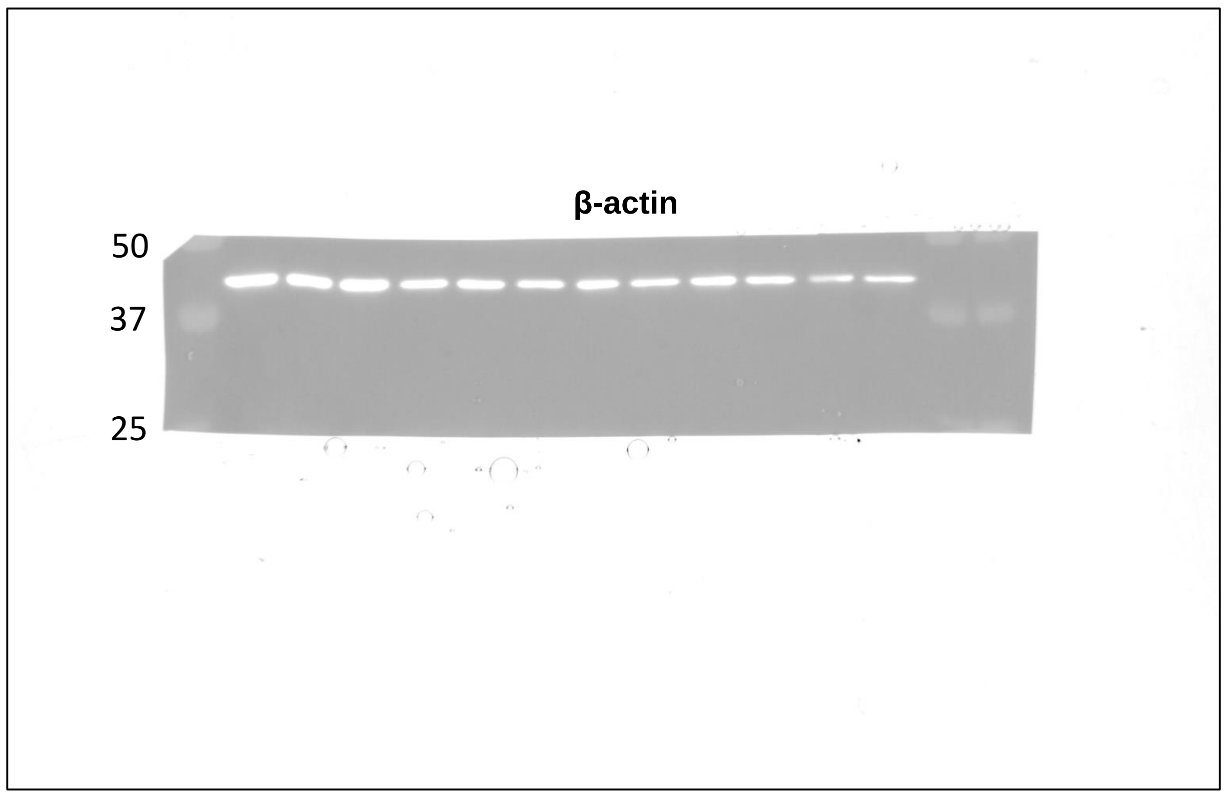

Supplement: Supplementary file 1 — Supplementary Figures. [file 41598_2021_3814_MOESM1_ESM.pdf]
